# Identifying Canadian Freshwater Fishes through DNA Barcodes

**DOI:** 10.1371/journal.pone.0002490

**Published:** 2008-06-18

**Authors:** Nicolas Hubert, Robert Hanner, Erling Holm, Nicholas E. Mandrak, Eric Taylor, Mary Burridge, Douglas Watkinson, Pierre Dumont, Allen Curry, Paul Bentzen, Junbin Zhang, Julien April, Louis Bernatchez

**Affiliations:** 1 Département de biologie, Pavillon Charles-Eugène-Marchand, Université Laval, Sainte-Foy, Québec, Canada; 2 Canadian Barcode of Life Network, Biodiversity Institute of Ontario, University of Guelph, Guelph, Ontario, Canada; 3 Department of Natural History, Royal Ontario Museum, Toronto, Ontario, Canada; 4 Great Lakes Laboratory for Fisheries and Aquatic Sciences, Fisheries and Oceans Canada, Burlington, Ontario, Canada; 5 Department of Zoology, Vancouver, British Columbia, Canada; 6 Fisheries and Oceans Canada, Central & Arctic Region, Freshwater Institute, Winnipeg, Manitoba, Canada; 7 Ministère des Ressources naturelles et de la faune du Québec, Direction de l'aménagement de la faune de Montréal, de Laval et de la Montérégie, Longueuil, Québec, Canada; 8 Fish and Wildlife Research Unit, University of New Brunswick, Fredericton, New Brunswick, Canada; 9 Department of Biology, Dalhousie University, Halifax, Nova Scotia, Canada; University of Uppsala, Sweden

## Abstract

**Background:**

DNA barcoding aims to provide an efficient method for species-level identifications using an array of species specific molecular tags derived from the 5′ region of the mitochondrial cytochrome c oxidase I (COI) gene. The efficiency of the method hinges on the degree of sequence divergence among species and species-level identifications are relatively straightforward when the average genetic distance among individuals within a species does not exceed the average genetic distance between sister species. Fishes constitute a highly diverse group of vertebrates that exhibit deep phenotypic changes during development. In this context, the identification of fish species is challenging and DNA barcoding provide new perspectives in ecology and systematics of fishes. Here we examined the degree to which DNA barcoding discriminate freshwater fish species from the well-known Canadian fauna, which currently encompasses nearly 200 species, some which are of high economic value like salmons and sturgeons.

**Methodology/Principal Findings:**

We bi-directionally sequenced the standard 652 bp “barcode” region of COI for 1360 individuals belonging to 190 of the 203 Canadian freshwater fish species (95%). Most species were represented by multiple individuals (7.6 on average), the majority of which were retained as voucher specimens. The average genetic distance was 27 fold higher between species than within species, as K2P distance estimates averaged 8.3% among congeners and only 0.3% among concpecifics. However, shared polymorphism between sister-species was detected in 15 species (8% of the cases). The distribution of K2P distance between individuals and species overlapped and identifications were only possible to species group using DNA barcodes in these cases. Conversely, deep hidden genetic divergence was revealed within two species, suggesting the presence of cryptic species.

**Conclusions/Significance:**

The present study evidenced that freshwater fish species can be efficiently identified through the use of DNA barcoding, especially the species complex of small-sized species, and that the present COI library can be used for subsequent applications in ecology and systematics.

## Introduction

DNA barcoding is designed to provide accurate, and automated species identifications through the use of molecular species tags based on short, standardised gene regions [1], [2]. While humanity is facing increasing evidence of the erosion of Earth's biodiversity, this approach is proving its effectiveness in characterising the complexity of the biodiversity realm at a pace unequalled by other characters [3]. The primary goals of DNA barcoding focus on the assembly of reference libraries of barcode sequences for known species in order to develop reliable, molecular tools for species identification in nature. Current results suggest that, in a large array of organisms, species are generally well delineated by a particular sequence or by a tight cluster of very similar sequences that allow unambiguous identifications [4], [5], [6], [7], [8], [9], [2], [10], [11], [12].

Despite the great promise of DNA barcoding, it has been controversial in some scientific circles [13], [14]. Yet, recent results illustrated some straightforward benefits from the use of a standardised molecular approach for identification [1], [2]. First, intraspecific phenotypic variation often overlaps that of sister taxa in nature, which can lead to incorrect identifications if based on phenotype only [e.g. 15]. Second, DNA barcodes are effective whatever the life stages under scrutiny [e.g. 16], [17]. Third, cryptic variation and often spectacular levels of undetected taxonomic diversity have been frequently reported [e.g. 18], [19], [20]. Finally, DNA barcode libraries are fully available as they are deposited in a major sequence database, and attached to a voucher specimen whose origin and current location are recorded [2], [3]. Once libraries are available, recent studies illustrate the vast array of applications that can be applied to them such as forensic engineering [21], [22], ecology of cryptic communities [23], the tracking of invasive species [24], [25] and identification of prey from predator stomach samples [e.g. 26].

With the aim of assigning specimens to known species based on molecular tags, a 648-bp segment of the 5′ region of mitochondrial cytochrome c oxidase I (COI) gene forms the library of primary barcodes for the animal kingdom [1]. Mitochondrial DNA (mtDNA) presents several advantages that make it well suited for large scale molecular tagging. First, this genome is present in a large number of copies yielding substantial amounts of genomic DNA from a variety of extraction methods. Second, the high mutation rate and small effective population size make it often an informative genome about evolutionary patterns and processes [27], [28]. For a barcoding approach to species identification to succeed, however, within-species DNA sequences need to be more similar to one another than to sequences in different species. Several processes such as pseudogenes ontogenesis, introgressive hybridisation, and retention of ancestral polymorphism pose potential difficulties in capturing species boundaries using mtDNA sequences [29], [30], [31], [32]. The detection of mixed genealogy between closely related species has been previously estimated to occur in nearly 20 percent of the cases in the wild [30]. Recent barcoding studies emphasised that this percent can vary widely among phyla, yet species assignment failures typically do not exceed 5 to 10 percent in a large array of organisms [2].

The economic importance and identification challenges associated with fishes prompted the launch of an international Fish Barcoding of Life (FISH-BOL) initiative (http://www.fishbol.org/) with the aim of barcoding all fishes. In the context of FISH-BOL and for the first time, we examine whether barcoding captures species boundaries and allows species identification among some of the major orders of primary freshwater fishes. Although COI divergence and species identification success has been previously assessed for some marine fishes [7], the average divergence found among freshwater fish species is unknown. The Canadian freshwater fish fauna has been subject to intensive taxonomic analysis for decades [33], [34], [35], [36], [37]. Thus, this fauna provides an excellent opportunity to test the efficacy of barcoded-based species delimitation and identification of freshwater fishes over a broad geographic range. Moreover, a large number of species from highly endangered and economically important groups such as salmon and sturgeon are found in Canada. Given their high diversity and dramatic phenotypic changes during development, fish species identification is no easy task. Hence, the development of reliable and universal molecular tags constitutes a major requirement for forensic engineering and conservation strategies involving such emblematic species.

## Materials and Methods

### BARCODE data standard and data management on BOLD

DNA Barcoding has greatly influenced the pace of sequence data acquisition. This approach prompted the development of new protocols and databases to manage the constitution of COI libraries for molecular identification. The Barcode of Life Data System (BOLD; see http://www.barcodinglife.org) was developed as a collaborative online workbench that has evolved into a resource for the DNA barcoding community [3]. The BOLD database currently host specimens records for which essentially, seven data elements are listed:

1. Species name
2. Voucher data
3. Collection record
4. Identifier of the specimen
5. COI sequence of at least 500 bp
6. PCR primers used to generate the amplicon
7. Trace files

**Figure 1:**
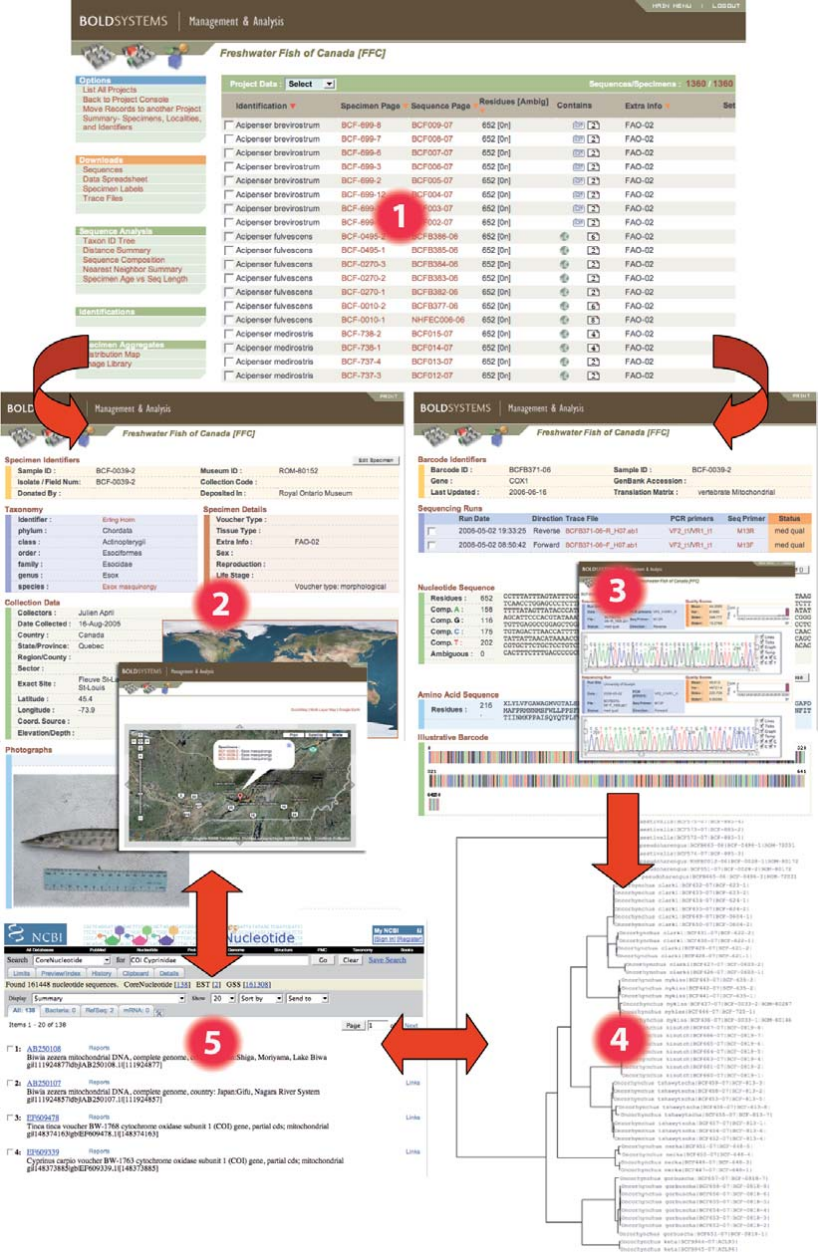
Structure of the Freshwater Fish of Canada (FFC) database in BOLD, functionalities and connections with others public databases. 1, Project page with the list of the specimens analysed including a link to the specimen and sequence page; 2, Specimen page for an individual of *Esox masquinongy* including voucher information, taxonomy, collection location, collection site maps and specimen image; 3, Sequence page for the same individual of *Esox masquinongy* including specimens details, sequencing details including links to trace files, amino acid translation of sequence and trace viewer; 4, Taxon ID tree for the Canadian members of the Salmonid genus *Oncorhynchus*; 5, Connections with the GenBank.

The core data element in BOLD is a biphasic record consisting of both a “specimen page” and a “sequence page” (Figure 1). Access to these pages is possible through direct link in the project console (1 in Figure 1) that includes a comprehensive list of all specimens included in the project. The specimen page (2 in Figure 1) assembles varied data about source of each specimen including the specimen's donor and identifier, taxonomy, collection data (including geospatial coordinates and digital images), the repository and catalog number of the voucher specimen. Each specimen page is coupled to a sequence page (3 in Figure 1) that records the barcode sequence (FASTA format), PCR primers and trace files, amino acid translation, and ultimately the GenBank accession number as well. Information from both the specimen and sequence pages can be incorporated into taxon ID trees that can be used in the identification system, while onboard mapping functions support investigations into spatial molecular ecology.

After preparing the barcode records in BOLD, data were uploaded into GenBank. Appendix S1 provides the voucher specimen ID, BOLD specimen record number, and GenBank accession number for each record. The Consortium for the Barcode of Life, in cooperation with GenBank and the other members of the International Nucleotide Sequence Database Collaboration (INSDC), have created and implemented the BARCODE data standard. “BARCODE” is a reserved keyword for those records in an INSDC database that meet a higher quality standard that makes them more reliable links between a gene sequence and a species name. All of the GenBank records created by this project and listed in Appendix S1 carry the BARCODE keyword because they include the following data:

1. Bi-directional sequences of at least 500 base-pairs from the approved barcode region of COI, containing no ambiguous sites
2. Links to electropherogram trace files available in the NCBI Trace Archive
3. Sequences for the forward and reverse PCR amplification primers
4. Species names that refer to documented names in a taxonomic publication or other documentation of the species concept used
5. Links to voucher specimens using the approved format of institutional acronym:collection code:catalog ID number

Taken together, the data required under the BARCODE data standard give researchers and other users with unprecedented access to data and metadata associated with the DNA sequence in GenBank. In addition, all of the information related to the present project is publicly available in the ‘Freshwater Fish of Canada’ projects (BCF and BCFB) on the Barcode of Life database (see http://www.barcodinglife.org) [3].

### Data acquisition and analytical tools

DNA sources for this study included either frozen or ethanol-fixed tissue samples (muscle, liver, blood, or fin). Samples for barcoding originated from expert-identified specimens based on morphological criteria (meristic, morphometric and colouration) currently recognized in recent monographs [33], [34], [35], [36], [37]. For each specimens, detailed geographic information and where possible, reference specimens were deposited as vouchers in publicly accessible collections. However, some tissues collected before the beginning of FISH-BOL were obtained through the support of fish taxonomists, particularly for species exhibiting remote geographic distribution. In that case, sequences were generated from tissues lacking proper morphological vouchers. In order to allow the repeatability of the sequences generated, the tissues used for extraction and amplifications were given the status of ‘tissue’ voucher and distinguished from traditional ‘morphological’ vouchers. Of the 1360 specimens analysed (190 species), 861 (127 species) sequences were obtained from specimens with vouchers housed in the collection of the Royal Ontario Museum, Toronto (Appendix S1). Hence, samples with specimens housed in museum collections represented 65% of the sequences and 70% of the species analysed in this study.

Previous comparative genetic surveys suggested that freshwater fishes generally exhibit higher levels of inter-population genetic diversity than marine fishes [38]. Hence, we aimed, where possible, to sample three to five individuals per site for at least two sites from different watersheds for widely distributed species to capture a representative part of the molecular diversity. Numbers of specimens per species ranged from one to 17 with a mean of 7.6; nearly twice the number of individuals per species previously analysed for marine fishes [7]. According to the General Status of Wildlife in Canada [39], the Canadian fauna currently includes 203 species of which 194 (96%) have been sampled during the present survey (Appendix S1).

DNA extractions were performed with the NucleoSpin96 (Machery-Nagel) kit according to the specification of the manufacturer under automation with a Biomek NX liquid-handling station (Beckman-Coulter) equipped with a filtration manifold as previously described [40], [41]. A 652-bp segment was amplified from the 5′ region of the mitochondrial COI gene using either the following primers FishF1-5′TCAACCAACCACAAAGACATTGGCAC3′ [7] and FishR1-5′TAGACTTCTGGGTGGCCAAAGAATCA3′ [7] or the primer cocktails (including M13 tails to facilitate sequencing) [42] when amplifications failed using the first set of primers. PCR amplifications were performed in 12.5 µl volume including 6.25 µl of 10% trehalose, 2 µl of ultra pure water, 1.25 µl of 10× PCR buffer (10mM Kcl, 10mM (NH_4_)_2_SO_4_, 20mM Tris-HCl (pH8.8), 2mM Mg SO_4_, 0.1% Triton X-100), 0.625 µl of MgCl_2_ (50mM), 0.125 µl of each primer (0.01mM), 0.0625 µl of each dNTP (10mM), 0.0625 µl of *Taq* DNA polymerase (New England Biolabs), and 2 µl of template DNA. The PCR conditions consisted of 94°C for 2 min, 35 cycles of 94°C for 30 s, 52°C 40 s, and 72°C for 1 min, with a final extension at 72°C for 10 min.

All the sequences have been deposited in GenBank and accession numbers for the barcodes, specimen and collection data, sequences, trace files and primers details are available within the BCF and BCFB project files in BOLD (http://www.barcodinglife.org). Sequence divergence was calculated using the Kimura 2-parameter (K2P) model [43] and the mid-point rooted Neighbour-joining (NJ) tree of K2P distances was created to provide a graphic representation of the species divergence [44] as implemented in the ‘Sequence Analysis’ module of BOLD. We checked for a potential sampling bias in the distribution of genetic diversity by plotting the mean intraspecific genetic distance between haplotypes against the number of individual analysed and tested the significance of the relationship using a covariance analysis as implemented in Statgraphics [45].

## Results

A total of 194 species have been sampled during the present survey and the primers used amplified the target region of all, but four species: *Ctenopharyngodon idella* (*n* = 2), *Lampetra richardsoni* (*n* = 5), *Lampetra camtschaticum* (*n* = 5) and *Catostomus columbianus* (*n* = 5). Thus, a total of 1360 COI barcodes of 652-bp have been obtained for 190 species distributed among 85 genera and 28 families (Appendix S1; BCF abd BCFB projects in BOLD). No insertions/deletions or codon stops were found, supporting the view that all of the amplified sequences constitute functional mitochondrial COI sequences. Moreover, all the amplified sequences were larger than 600-bp, the limit typically observed for nuclear DNA sequences originating from mtDNA (NUMTs) [31]. The entire K2P/NJ tree derived from this study is available in Appendix S2 (or can be generated using BOLD).

Average intraspecific variation was unrelated to the number of individuals analysed (average intraspecific K2P distance = 0.015*N*+0.135; Covariance Analysis; *F* = 2.22; *P* = 0.138), suggesting representative sampling for the different species. The mean K2P distance of individual within species was 0.302 compared with 8.286 for species within genera (Table 1). Hence, overall, there was a 27-fold more pronounced difference among congeneric species than among conspecific individuals. Distributions of mean K2P distances among conspecific individuals and among congeneric species, however, partially overlapped as K2P distances ranged from 0 to 7.416 among conspecifics and 0 to 19.326 among congeneric species (Table 1).

**Table 1:** Summary of genetic divergences (K2P model used for computing distances) for increasing taxonomic levels. Data are from 1360 sequences from 190 species and 85 genera.

Comparisons within	Taxa	Number of comparisons	Min	Mean	Max	SE
Species	190	5865	0	0.27	7.42	0.01
Genus, among Species	85	18933	0	8.37	19.33	0.03
Family, among Genus	28	96992	2.67	15.38	23.22	0.01
Order, among Families	20	76571	14.25	20.06	29.44	0.01
Class, among Orders	2	681968	17.49	24.57	31.20	0.002

**Figure 2:**
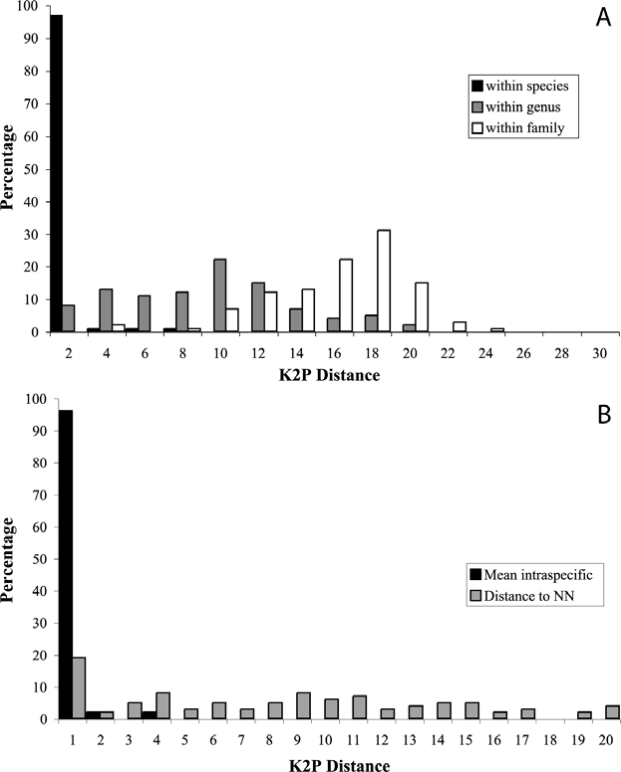
Summary of the distribution of the genetic variability (K2P distances) at COI sequences for the 1360 individuals and 190 species analysed. A. Distribution of the genetic distance within different taxonomic categories. B. Distribution of the genetic distances to the nearest-neighbour and mean intra-specific distance.

A steady increase of genetic variation through increasing taxonomic levels was observed, supporting a marked change of genetic divergence at the species boundaries (Figure 2A). The analysis of the distribution of the nearest-neighbour distance (NND), namely the minimum genetic distance between a species and its closest congeneric relative revealed that only 20% of the NND was lower than 1% (Figure 2B) and only 7% of the NND (14 cases) were lower than 0.1% (Table 2). By contrast, the divergence between conspecific individuals was lower than 1% in 96% of cases. NND averaged 7.5%, which was 30-fold higher than the mean within species distance of around 0.3% and 13-fold higher than the mean maximum intraspecific distance of around 0.6%. Overlap in the distribution of the genetic distances between conspecifics individuals and congeneric species may originate from deep intraspecific divergences and low sister-species divergence.

**Table 2:** Summary of the Canadian freshwater fish diversity and distribution of the genetic distance of each of the 190 species analysed to the nearest-neighbour at COI (K2P model used for computing distances).

		Number of species					
Order	Family	recorded	barcoded	<0.1	0.1–1.0	1.0–2.7	>2.7
Pleuronectiformes	Pleuronectidae	1	1	0	0	0	1
Cypriniformes	Cyprinidae	54	50	3	6	1	40
	Catostomidae	18	17	0	2	3	12
Scorpaeniformes	Cottidae	9	8	0	2	3	3
Salmoniformes	Salmonidae	29	29	7	6	3	13
Esociformes	Umbridae	2	2	0	0	0	2
	Esocidae	4	4	0	2	0	2
Clupeiformes	Clupeidae	4	4	0	2	0	2
Cyprinodontiformes	Fundulidae	3	3	0	0	0	3
Perciformes	Percidae	16	16	2	0	0	14
	Centrarchidae	13	12	0	0	3	9
	Percichthyidae	3	3	0	0	0	3
	Gobiidae	2	2	0	0	0	2
	Sciaenidae	1	1	0	0	0	1
Gasterosteiformes	Gasterosteidae	5	5	0	0	0	5
Siluriformes	Ictaluridae	10	10	0	0	2	8
Osmeriformes	Osmeridae	4	3	0	0	0	3
Semionotiformes	Lepisosteidae	2	2	0	0	0	2
Acipenseriformes	Acipenseridae	5	5	0	0	2	3
Osteoglossiformes	Hiodontidae	2	2	0	0	0	2
Petromyzontiformes	Petromyzontidae	10	5	2	0	0	3
Percopsiformes	Percopsidae	1	1	0	0	0	1
Gadiformes	Lotidae	1	1	0	0	0	1
	Gadidae	1	1	0	0	0	1
Atheriniformes	Atherinopsidae	1	1	0	0	0	1
Anguilliformes	Anguillidae	1	1	0	0	0	1
Amiiformes	Amiidae	1	1	0	0	0	1
	Total	203	190	14	20	17	139

In a few cases, we detected deep divergences among individuals that had been assigned to single species. Two lineages, one in the Laurentian Great Lakes area and another one in the St Lawrence River and diverging from 1% to 2% from each other were observed in five species including the common shiner (*Luxilus cornutus*), fathead minnow (*Pimephales promelas*), finescale dace (*Phoxinus neogaeus*), golden shiner (*Notemigonus crysoleucas*) and fantail darter, *Etheostoma flabellare* (Appendix S2). The same pattern was found among samples from the brook stickleback *Culaea inconstans* and the redfin pickerel, *Esox americanus*, where the divergence was even greater as it reached 7% and 3%, respectively. This result supports a genetic differentiation of the two *Esox americanus* subspecies *E. americanus americanus* from the St Lawrence River and *E. americanus vermiculatus* from the Laurentian Great Lakes area to the west. Although a single haplotype was found for each subspecies, more genetic divergence was observed between these two subspecies than with *Esox niger* since *E. americanus* was paraphyletic with its genealogy encompassing that of *Esox niger*. Likewise, a lineage found in the Pacific coast and diverging by 1.5% from the eastern samples was observed in the mottled sculpin, *Cottus bairdii*. Moreover, the Pacific lineage of *C. bairdii* was more closely related to the slimy sculpin, *Cottus cognatus*, than other conspecific samples. This suggests that a careful reappraisal of the current taxonomy for these groups could prove informative.

Cases of shared barcode haplotypes were detected in 13 (7%) of the species analysed including the following pairs: between the lampreys *Ichthyomyzon fossor* and *I. unicuspis*, between the shiners *Notropis volucellus* and *N. buchanani*, between the shad *Alosa aestivalis* and *A. pseudoharengus*, between the putative species in the cisco species flock, *Coregonus artedi*, *C. hoyi*, *C. kiyi*, *C. nigripinnis* and *C. zenithicus*; and, between the darters *Etheostoma nigrum* and *E. olmstedi*. Nevertheless, we only found evidence of introgressive hybridisation between two diverging species in the case of the darters *Etheostoma nigrum* and *E. olmstedi* with two clades diverging by nearly 6%, each one more closely associated with one of the two species. In all the other cases, COI sequences of the mixed species were tightly clustered and differed by less than 0.1% divergence (Table 2).

## Discussion

This study has shown the efficacy of COI barcodes for diagnosing North American freshwater fishes since most species examined here corresponded to a single, cohesive array of barcode sequences that are distinct from those of any other species. The success of the barcoding approach depends on the distribution of genetic distances between conspecific individuals and heterospecific individuals given that failures in barcode clustering are proportional to the overlap between both distributions [46]. It has been shown that lineages diversify more quickly within species than between species [47]. This is due to the fact that diversification within species is driven by mutation at a rate higher than speciation within lineages. Hence, the branch length between species tends to be much deeper than between conspecific individuals leading to a gap in the distribution of the pairwise distance between conspecific individuals and between species that has been referred to the barcoding gap [46]. The COI locus harbours a high mutational rate even for mtDNA [48]. The present study confirms that, in the vast majority of the taxa examined here (93%), the barcoding gap was observed and the mean genetic distance between conspecifics was generally much smaller than the average distance between individual from distinct species, even if only the sister species were considered.

Although barcode analyses primarily seek to delineate species boundaries at the COI locus for the assignment of unknown individuals to known species, unsuspected diversity and overlooked species are often detected through barcodes analyses, sometimes spectacularly [10], [18], [47]. The average distance between conspecific individuals was around 0.3% while average NND and average distance between congeneric species were 7.5% and 8.3%, respectively. When screening for species splits using a threshold of 1% (3 fold higher than the average intraspecific variability), nine species exhibited lineages falling out of the average divergence between conspecific individuals.

Among the set of 190 species, however, 13 species (7%) exhibited barcode sequences that were shared or overlapped with those of other species. Regarding these cases, at least three factors may be involved [30], [46]. First, the establishment of reciprocal monophyly between two sister species is a function of time given that fixation of a new coalescent follow the line of descent framework from the coalescent theory [49], [50]. Second, the taxa may share polymorphism due to introgressive hybridisation. If hybridisation is due to secondary contact after a stage of isolation and genetic drift, introgressive hybridisation may be detected due to the presence of two divergent clusters, each one being found predominantly in one species or the other. Finally, the barcoding approach first examines species delineation through COI barcodes for species established generally through a traditional approach of taxonomy using phenotypes. Some of the pairs with overlapping barcodes, however, may be a single species. Alternatively, the use of uniform threshold may be a source of error leading to erroneous assignment of individuals to species [51], [52]. In the present case, 34 species would have been undetected by using a 1% threshold. Providing that seven species share polymorphism or harbour mixed genealogy, 24 species with monophyletic COI lineages would have been overlooked with a 1% threshold. Yet, the development of assignment tools based on more realist probabilistic models under a coalescent framework will likely solve this problem and enhanced the statistical power of individual assignment through the use of a single gene [53], [54].

The present study is the first to assess the resolution of barcoding for freshwater fish species from a variety of primary freshwater groups. It is widely appreciated that the fragmentation of the rivers and lakes from continental freshwater network leads to more pronounced genetic structure among populations and deeper divergence among haplotypes than in the marine realm [38]. In the largest barcoding study conducted so far on marine fishes to date [7], the average observed distance between conspecifics was 0.4% while the average divergence reached 9.9% between congeneric species. However, the average distance between conspecifics and congeneric species reached 0.3% and 8.3%, respectively, for freshwater fishes in this study, a pattern strikingly similar to that of marine fishes. Although geographic structure was often detected here among populations, the present survey suggests that the higher geographic structure of freshwater fishes is not necessarily reflected in deeper intraspecific and interspecific divergence than marine species. Although, we failed to capture a substantial amount of population diversity through the present sampling, it remains unlikely that sampling artefacts alone can account for similar intraspecific divergences found among freshwater and marine species. Admittedly however, the Canadian freshwater fish fauna may not be representative of old established population diversity since most of the rivers and lakes of the country have been colonised after the glacial retreat at the end of the Pleistocene [55].

In summary, most of the North American freshwater fish species analysed here exhibit a similar pattern of genetic diversity at COI, each being a single cluster of tightly related mtDNA sequences distinct from all other species. Therefore, the present survey supports the view that the use of COI barcodes is a powerful tool for species identification. Using this method would clearly allow the identification of individually isolated freshwater fish eggs, larvae, fillets and fins, hence providing many news tools useful for the practice of conservation and forensics genetic in these freshwater fishes. From a systematic perspective, COI barcodes provide a new and fast approach for screening the real number of species characterised by private sets of diagnostic characters. The identification of several cases of polyphyletic or paraphyletic COI species genealogy further supports the view that an iterative process of DNA barcoding followed by taxonomic analyses using other characters will be a productive way to catalogue biodiversity [10], [56]. The present data set coupled with the functionality in BOLD provides a tool that is already operational for molecular assisted identification of the Canadian species. The entire cataloguing of the North American freshwater fish fauna, which is currently being undertaken by FISH-BOL, will result in a significant improvement of our knowledge concerning the systematic of the freshwater fishes of the region and also facilitate monitoring changes in the geographic distribution of species that will probably occur in the future.

## Supporting Information

Appendix S1 Details of species and specimens. Barcode of Life Database (BOLD) specimen numbers given, along with GenBank accession numbers, geographic locality and voucher details.
(1.28 MB DOC)

Appendix S2 Neighbour-joining tree of 1360 COI sequences from the 190 freshwater fish species sampled as obtained in BOLD, using K2P distances.
(0.95 MB PDF)
