# Supplementary material for: Identifying Canadian Freshwater Fishes through DNA Barcodes
Source: PLoS One. 2008 Jun 18;3(6):e2490. doi: 10.1371/journal.pone.0002490 (PMC3278308; doi:10.1371/journal.pone.0002490)
Supplement: Appendix S1 — Details of species and specimens. Barcode of Life Database (BOLD) specimen numbers given, along with GenBank accession numbers, geographic locality and voucher details. (1.28 MB DOC) [file pone.0002490.s001.doc]

|  |  |  |  | BOLD | GenBank |
| --- | --- | --- | --- | --- | --- |
| Species name | Geographic locality | Voucher type | Voucher number | Specimen number | Accession number |
| Acipenser brevirostrum | Canada: New Brunswick: Aquaculture | tissue | UOG:Bio:BCF-0699-8 | BCF-0699-8 | EU523870 |
| Acipenser brevirostrum | Canada: New Brunswick: Aquaculture | tissue | UOG:Bio:BCF-0699-7 | BCF-0699-7 | EU523871 |
| Acipenser brevirostrum | Canada: New Brunswick: Aquaculture | tissue | UOG:Bio:BCF-0699-6 | BCF-0699-6 | EU523872 |
| Acipenser brevirostrum | Canada: New Brunswick: Aquaculture | tissue | UOG:Bio:BCF-0699-3 | BCF-0699-3 | EU523873 |
| Acipenser brevirostrum | Canada: New Brunswick: Aquaculture | tissue | UOG:Bio:BCF-0699-2 | BCF-0699-2 | EU523874 |
| Acipenser brevirostrum | Canada: New Brunswick: Aquaculture | tissue | UOG:Bio:BCF-0699-12 | BCF-0699-12 | EU523875 |
| Acipenser brevirostrum | Canada: New Brunswick: Aquaculture | tissue | UOG:Bio:BCF-0699-11 | BCF-0699-11 | EU523876 |
| Acipenser brevirostrum | Canada: New Brunswick: Aquaculture | tissue | UOG:Bio:BCF-0699-10 | BCF-0699-10 | EU523877 |
| Acipenser fulvescens | Canada: Quebec: Fleuve St-Laurent, Lac St-Pierre | tissue | UOG:Bio:BCF-010-1 | BCF-010-1 | EU523878 |
| Acipenser fulvescens | Canada: Ontario: Georgian Bay | morphological | ROM:Ich:BCF-0495-2 | BCF-0495-2 | EU524392 |
| Acipenser fulvescens | Canada: Ontario: Georgian Bay | morphological | ROM:Ich:BCF-0495-1 | BCF-0495-1 | EU524393 |
| Acipenser fulvescens | Canada: Quebec: Fleuve St-Laurent, riviere St-Nicolas | tissue | UOG:Bio:BCF-0270-3 | BCF-0270-3 | EU524394 |
| Acipenser fulvescens | Canada: Quebec: Fleuve St-Laurent, riviere St-Nicolas | tissue | UOG:Bio:BCF-0270-2 | BCF-0270-2 | EU524395 |
| Acipenser fulvescens | Canada: Quebec: Fleuve St-Laurent, riviere St-Nicolas | tissue | UOG:Bio:BCF-0270-1 | BCF-0270-1 | EU524396 |
| Acipenser fulvescens | Canada: Quebec: Fleuve St-Laurent, Lac St-Pierre | tissue | UOG:Bio:BCF-010-2 | BCF-010-2 | EU524397 |
| Acipenser medirostris | United States: Washington: Rogue river | tissue | UOG:Bio:BCF-0738-3 | BCF-0738-3 | EU523879 |
| Acipenser medirostris | United States: Washington: Rogue river | tissue | UOG:Bio:BCF-0738-2 | BCF-0738-2 | EU523880 |
| Acipenser medirostris | United States: Washington: Rogue river | tissue | UOG:Bio:BCF-0738-1 | BCF-0738-1 | EU523881 |
| Acipenser medirostris | United States: Washington: Sacramento river | tissue | UOG:Bio:BCF-0737-4 | BCF-0737-4 | EU523882 |
| Acipenser medirostris | United States: Washington: Sacramento river | tissue | UOG:Bio:BCF-0737-3 | BCF-0737-3 | EU523883 |
| Acipenser medirostris | United States: Washington: Sacramento river | tissue | UOG:Bio:BCF-0737-2 | BCF-0737-2 | EU523884 |
| Acipenser medirostris | United States: Washington: Sacramento river | tissue | UOG:Bio:BCF-0737-1 | BCF-0737-1 | EU523885 |
| Acipenser oxyrynchus | Canada: Quebec: Fleuve Saint-Laurent, Ile Madamme | morphological | ROM:Ich:BCF-009-1 | BCF-009-1 | EU523886 |
| Acipenser oxyrynchus | Canada: Quebec: Fleuve St-Laurent, Ile Madame | morphological | ROM:Ich:BCF-009-5 | BCF-009-5 | EU524398 |
| Acipenser oxyrynchus | Canada: Quebec: Fleuve St-Laurent, Ile Madamme | morphological | ROM:Ich:BCF-009-4 | BCF-009-4 | EU524399 |
| Acipenser oxyrynchus | Canada: Quebec: Fleuve St-Laurent, Ile Madamme | morphological | ROM:Ich:BCF-009-3 | BCF-009-3 | EU524400 |
| Acipenser oxyrynchus | Canada: Quebec: Fleuve St-Laurent, Ile Madamme | morphological | ROM:Ich:BCF-009-2 | BCF-009-2 | EU524401 |
| Acipenser transmontanus | United States: Washington: Nechako reservoir | tissue | UOG:Bio:BCF-0735-3 | BCF-0735-3 | EU523887 |
| Acipenser transmontanus | United States: Washington: Nechako reservoir | tissue | UOG:Bio:BCF-0735-2 | BCF-0735-2 | EU523888 |
| Acipenser transmontanus | United States: Washington: Nechako reservoir | tissue | UOG:Bio:BCF-0735-1 | BCF-0735-1 | EU523889 |
| Acipenser transmontanus | United States: Washington: Fraser river | tissue | UOG:Bio:BCF-0734-3 | BCF-0734-3 | EU523890 |
| Acipenser transmontanus | United States: Washington: Fraser river | tissue | UOG:Bio:BCF-0734-1 | BCF-0734-1 | EU523891 |
| Amia calva | Canada: Quebec: Riviere Richelieu, Saint-Ours | tissue | UOG:Bio:BCF-0014-1 | BCF-0014-1 | EU523910 |
| Amia calva | Canada: Quebec: Fleuve St-Laurent, lac St-Paul | tissue | UOG:Bio:BCF-0015-1 | BCF-0015-1 | EU524434 |
| Amia calva | Canada: Quebec: Fleuve St-Laurent, riviere Richelieu | tissue | UOG:Bio:BCF-0014-2 | BCF-0014-2 | EU524435 |
| Anguilla rostrata | Canada: Quebec: Riviere Laval | morphological | ROM:Ich:BCF-0016-1 | BCF-0016-1 | EU523918 |
| Anguilla rostrata | Canada: New Brunswick: Mc Quarrie Brook | morphological | ROM:Ich:BCF-0582-5 | BCF-0582-5 | EU524436 |
| Anguilla rostrata | Canada: New Brunswick: Mc Quarrie Brook | morphological | ROM:Ich:BCF-0582-4 | BCF-0582-4 | EU524437 |
| Anguilla rostrata | Canada: New Brunswick: Mc Quarrie Brook | morphological | ROM:Ich:BCF-0582-2 | BCF-0582-2 | EU524438 |
| Anguilla rostrata | Canada: New Brunswick: Mc Quarrie Brook | morphological | ROM:Ich:BCF-0582-1 | BCF-0582-1 | EU524439 |
| Anguilla rostrata | Canada: Ontario: Lake Simcoe | morphological | ROM:Ich:BCF-0538-1 | BCF-0538-1 | EU524440 |
| Anguilla rostrata | Canada: Ontario: Lake Joseph | morphological | ROM:Ich:BCF-0425-1 | BCF-0425-1 | EU524441 |
| Anguilla rostrata | Canada: Ontario: Lake Simcoe | morphological | ROM:Ich:BCF-0402-1 | BCF-0402-1 | EU524442 |
| Labidesthes sicculus | Canada: Quebec: Riviere Richelieu | morphological | ROM:Ich:BCF-0130-1 | BCF-0130-1 | EU524108 |
| Labidesthes sicculus | Canada: Ontario: Fleuve St-Laurent | morphological | ROM:Ich:BCF-0350-3 | BCF-0350-3 | EU524689 |
| Labidesthes sicculus | Canada: Ontario: Fleuve St-Laurent | morphological | ROM:Ich:BCF-0350-2 | BCF-0350-2 | EU524690 |
| Labidesthes sicculus | Canada: Ontario: Fleuve St-Laurent | morphological | ROM:Ich:BCF-0350-1 | BCF-0350-1 | EU524691 |
| Labidesthes sicculus | Canada: Quebec: Fleuve St-Laurent, lac St-Louis | morphological | ROM:Ich:BCF-0132-3 | BCF-0132-3 | EU524692 |
| Labidesthes sicculus | Canada: Quebec: Fleuve St-Laurent, lac St-Louis | morphological | ROM:Ich:BCF-0132-2 | BCF-0132-2 | EU524693 |
| Labidesthes sicculus | Canada: Quebec: Fleuve St-Laurent, lac St-Louis | morphological | ROM:Ich:BCF-0132-1 | BCF-0132-1 | EU524694 |
| Labidesthes sicculus | Canada: Quebec: Fleuve St-Laurent, lac St-Paul | morphological | ROM:Ich:BCF-0131-3 | BCF-0131-3 | EU524695 |
| Labidesthes sicculus | Canada: Quebec: Fleuve St-Laurent, lac St-Paul | morphological | ROM:Ich:BCF-0131-2 | BCF-0131-2 | EU524696 |
| Labidesthes sicculus | Canada: Quebec: Fleuve St-Laurent, lac St-Paul | morphological | ROM:Ich:BCF-0131-1 | BCF-0131-1 | EU524697 |
| Labidesthes sicculus | Canada: Quebec: Fleuve St-Laurent, riviere Richelieu | morphological | ROM:Ich:BCF-0130-2 | BCF-0130-2 | EU524698 |
| Carpiodes cyprinus | Canada: Quebec: Lac Saint-Louis (Fleuve Saint-Laurent) | tissue | UOG:Bio:BCF-0102-1 | BCF-0102-1 | EU523924 |
| Carpiodes cyprinus | Canada: Quebec: Fleuve St-Laurent, riviere St-Nicolas | tissue | UOG:Bio:BCF-0033-1 | BCF-0033-1 | EU524451 |
| Carpiodes cyprinus | Canada: Ontario: Lake Erie | morphological | ROM:Ich:BCF-0551-1 | BCF-0551-1 | EU524452 |
| Carpiodes cyprinus | Canada: Quebec: Fleuve St-Laurent, lac St-Pierre | morphological | ROM:Ich:BCF-0103-3 | BCF-0103-3 | EU524453 |
| Carpiodes cyprinus | Canada: Quebec: Fleuve St-Laurent, lac St-Pierre | morphological | ROM:Ich:BCF-0103-2 | BCF-0103-2 | EU524454 |
| Carpiodes cyprinus | Canada: Quebec: Fleuve St-Laurent, lac St-Pierre | morphological | ROM:Ich:BCF-0103-1 | BCF-0103-1 | EU524455 |
| Carpiodes cyprinus | Canada: Quebec: Baie Missisquoi | tissue | UOG:Bio:BCF-0101-3 | BCF-0101-3 | EU524456 |
| Carpiodes cyprinus | Canada: Quebec: Baie Missisquoi | tissue | UOG:Bio:BCF-0101-2 | BCF-0101-2 | EU524457 |
| Carpiodes cyprinus | Canada: Quebec: Baie Missisquoi | tissue | UOG:Bio:BCF-0101-1 | BCF-0101-1 | EU524458 |
| Carpiodes cyprinus | Canada: Quebec: Fleuve St-Laurent, riviere Richelieu | tissue | UOG:Bio:BCF-0100-3 | BCF-0100-3 | EU524459 |
| Carpiodes cyprinus | Canada: Quebec: Fleuve St-Laurent, riviere Richelieu | tissue | UOG:Bio:BCF-0100-2 | BCF-0100-2 | EU524460 |
| Carpiodes cyprinus | Canada: Quebec: Fleuve St-Laurent, riviere Richelieu | tissue | UOG:Bio:BCF-0100-1 | BCF-0100-1 | EU524461 |
| Catostomus catostomus | Canada: British Columbia: Fraser river | tissue | UOG:Bio:BCF-0670-5 | BCF-0670-5 | EU523925 |
| Catostomus catostomus | Canada: British Columbia: Fraser river | tissue | UOG:Bio:BCF-0670-4 | BCF-0670-4 | EU523926 |
| Catostomus catostomus | Canada: British Columbia: Fraser river | tissue | UOG:Bio:BCF-0670-3 | BCF-0670-3 | EU523927 |
| Catostomus catostomus | Canada: British Columbia: Fraser river | tissue | UOG:Bio:BCF-0670-2 | BCF-0670-2 | EU523928 |
| Catostomus catostomus | Canada: British Columbia: Fraser river | tissue | UOG:Bio:BCF-0670-1 | BCF-0670-1 | EU523929 |
| Catostomus catostomus | Canada: Quebec: Saint Lawrence River St-Nicolas | tissue | UOG:Bio:BCF-0112-1 | BCF-0112-1 | EU523930 |
| Catostomus catostomus | Canada: Ontario: Lake Ontario | morphological | ROM:Ich:BCF-0523-1 | BCF-0523-1 | EU524462 |
| Catostomus catostomus | Canada: Ontario: Pine River | morphological | ROM:Ich:BCF-0434-4 | BCF-0434-4 | EU524463 |
| Catostomus catostomus | Canada: Ontario: Pine River | morphological | ROM:Ich:BCF-0434-3 | BCF-0434-3 | EU524464 |
| Catostomus catostomus | Canada: Ontario: Pine River | morphological | ROM:Ich:BCF-0434-2 | BCF-0434-2 | EU524465 |
| Catostomus catostomus | Canada: Ontario: Pine River | morphological | ROM:Ich:BCF-0434-1 | BCF-0434-1 | EU524466 |
| Catostomus catostomus | Canada: Quebec: Fleuve St-Laurent, riviere Ste-Marguerite | tissue | UOG:Bio:BCF-0266-2 | BCF-0266-2 | EU524467 |
| Catostomus catostomus | Canada: Quebec: Fleuve St-Laurent, riviere St-Nicolas | tissue | UOG:Bio:BCF-0112-2 | BCF-0112-2 | EU524468 |
| Catostomus catostomus | Canada: Quebec: Fleuve St-Laurent, riviere Richelieu | tissue | UOG:Bio:BCF-0111-3 | BCF-0111-3 | EU524469 |
| Catostomus catostomus | Canada: Quebec: Fleuve St-Laurent, riviere Richelieu | tissue | UOG:Bio:BCF-0111-2 | BCF-0111-2 | EU524470 |
| Catostomus catostomus | Canada: Quebec: Fleuve St-Laurent, riviere Richelieu | tissue | UOG:Bio:BCF-0111-1 | BCF-0111-1 | EU524471 |
| Catostomus commersonii | Canada: Quebec: Lac Saint-Louis (Fleuve Saint-Laurent) | morphological | ROM:Ich:BCF-0104-1 | BCF-0104-1 | EU523931 |
| Catostomus commersonii | Canada: New Brunswick: Gapetown | tissue | UOG:Bio:BCF-0579-4 | BCF-0579-4 | EU524472 |
| Catostomus commersonii | Canada: New Brunswick: Gapetown | tissue | UOG:Bio:BCF-0579-3 | BCF-0579-3 | EU524473 |
| Catostomus commersonii | Canada: New Brunswick: Gapetown | tissue | UOG:Bio:BCF-0579-2 | BCF-0579-2 | EU524474 |
| Catostomus commersonii | Canada: New Brunswick: Gapetown | tissue | UOG:Bio:BCF-0579-1 | BCF-0579-1 | EU524475 |
| Catostomus commersonii | Canada: Ontario: Credit River | morphological | ROM:Ich:BCF-0435-2 | BCF-0435-2 | EU524476 |
| Catostomus commersonii | Canada: Ontario: Credit River | morphological | ROM:Ich:BCF-0435-1 | BCF-0435-1 | EU524477 |
| Catostomus commersonii | Canada: Ontario: Lake Ontario | morphological | ROM:Ich:BCF-0426-1 | BCF-0426-1 | EU524478 |
| Catostomus commersonii | Canada: Ontario: Sydenham River | morphological | ROM:Ich:BCF-0403-2 | BCF-0403-2 | EU524479 |
| Catostomus commersonii | Canada: Ontario: Sydenham River | morphological | ROM:Ich:BCF-0403-1 | BCF-0403-1 | EU524480 |
| Catostomus commersonii | Canada: Quebec: Fleuve St-Laurent, riviere Richelieu | tissue | UOG:Bio:BCF-0107-3 | BCF-0107-3 | EU524481 |
| Catostomus commersonii | Canada: Quebec: Fleuve St-Laurent, lac St-Louis | morphological | ROM:Ich:BCF-0104-4 | BCF-0104-4 | EU524482 |
| Catostomus commersonii | Canada: Quebec: Fleuve St-Laurent, lac St-Louis | morphological | ROM:Ich:BCF-0104-3 | BCF-0104-3 | EU524483 |
| Catostomus commersonii | Canada: Quebec: Fleuve St-Laurent, lac St-Louis | morphological | ROM:Ich:BCF-0104-2 | BCF-0104-2 | EU524484 |
| Catostomus macrocheilus | Canada: British Columbia: Mission creek | tissue | UOG:Bio:BCF-0658-2 | BCF-0658-2 | EU523932 |
| Catostomus macrocheilus | Canada: British Columbia: Salwein creek | tissue | UOG:Bio:BCF-0658-1 | BCF-0658-1 | EU523933 |
| Catostomus platyrhynchus | Canada: Saskatchewan: Belly River, Saskatchewan River | tissue | UOG:Bio:BCF-0874-1 | BCF-0874-1 | EU522454 |
| Catostomus platyrhynchus | Canada: Saskatchewan: Saskatchewan River, Lee creek | tissue | UOG:Bio:BCF-0779-5 | BCF-0779-5 | EU523934 |
| Catostomus platyrhynchus | Canada: Saskatchewan: Saskatchewan River, Lee creek | tissue | UOG:Bio:BCF-0779-4 | BCF-0779-4 | EU523935 |
| Catostomus platyrhynchus | Canada: Saskatchewan: Saskatchewan River, Lee creek | tissue | UOG:Bio:BCF-0779-3 | BCF-0779-3 | EU523936 |
| Catostomus platyrhynchus | Canada: Saskatchewan: Saskatchewan River, Lee creek | tissue | UOG:Bio:BCF-0779-2 | BCF-0779-2 | EU523937 |
| Catostomus platyrhynchus | Canada: Saskatchewan: Saskatchewan River, Lee creek | tissue | UOG:Bio:BCF-0779-1 | BCF-0779-1 | EU523938 |
| Erimyzon sucetta | Canada: Ontario: Long Point Bay | morphological | ROM:Ich:BCF-0514-1 | BCF-0514-1 | EU524567 |
| Hypentelium nigricans | Canada: Ontario: Big Otter Creek | morphological | ROM:Ich:BCF-0413-1 | BCF-0413-1 | EU524667 |
| Hypentelium nigricans | Canada: Ontario: Grand River | morphological | ROM:Ich:BCF-0393-4 | BCF-0393-4 | EU524668 |
| Hypentelium nigricans | Canada: Ontario: Grand River | morphological | ROM:Ich:BCF-0393-3 | BCF-0393-3 | EU524669 |
| Hypentelium nigricans | Canada: Ontario: Grand River | morphological | ROM:Ich:BCF-0393-2 | BCF-0393-2 | EU524670 |
| Hypentelium nigricans | Canada: Ontario: Grand River | morphological | ROM:Ich:BCF-0393-1 | BCF-0393-1 | EU524671 |
| Hypentelium nigricans | Canada: Ontario: Thames River | morphological | ROM:Ich:BCF-0376-5 | BCF-0376-5 | EU524672 |
| Hypentelium nigricans | Canada: Ontario: Thames River | morphological | ROM:Ich:BCF-0376-4 | BCF-0376-4 | EU524673 |
| Hypentelium nigricans | Canada: Ontario: Thames River | morphological | ROM:Ich:BCF-0376-3 | BCF-0376-3 | EU524674 |
| Hypentelium nigricans | Canada: Ontario: Thames River | morphological | ROM:Ich:BCF-0376-1 | BCF-0376-1 | EU524675 |
| Ictiobus cyprinellus | Canada: Ontario: Lake Ontario | morphological | ROM:Ich:BCF-0488-1 | BCF-0488-1 | EU524687 |
| Ictiobus cyprinellus | Canada: Ontario: Welland River | morphological | ROM:Ich:BCF-0502-1 | BCF-0502-1 | EU524688 |
| Ictiobus niger | United States: Kansas | tissue | UOG:Bio:BCF-0707-1 | BCF-0707-1 | EU524107 |
| Minytrema melanops | Canada: Ontario: East shore, Down Island | tissue | UOG:Bio:BCF-0566-5 | BCF-0566-5 | EU524839 |
| Minytrema melanops | Canada: Ontario: Detroit River | tissue | UOG:Bio:BCF-0566-23 | BCF-0566-23 | EU524840 |
| Minytrema melanops | Canada: Ontario: Detroit River | tissue | UOG:Bio:BCF-0566-21 | BCF-0566-21 | EU524841 |
| Minytrema melanops | Canada: Ontario: St Clair river | tissue | UOG:Bio:BCF-0566-17 | BCF-0566-17 | EU524842 |
| Minytrema melanops | Canada: Ontario: St Clair river | tissue | UOG:Bio:BCF-0566-16 | BCF-0566-16 | EU524843 |
| Minytrema melanops | Canada: Ontario: St Clair river | tissue | UOG:Bio:BCF-0566-15 | BCF-0566-15 | EU524844 |
| Minytrema melanops | Canada: Ontario: St Clair river | tissue | UOG:Bio:BCF-0566-12 | BCF-0566-12 | EU524845 |
| Moxostoma anisurum | Canada: Quebec: Lac Saint-Louis (Fleuve Saint-Laurent) | morphological | ROM:Ich:BCF-0095-1 | BCF-0095-1 | EU524146 |
| Moxostoma anisurum | Canada: Ontario: Sydenham River | morphological | ROM:Ich:BCF-0415-3 | BCF-0415-3 | EU524846 |
| Moxostoma anisurum | Canada: Ontario: Sydenham River | morphological | ROM:Ich:BCF-0415-1 | BCF-0415-1 | EU524847 |
| Moxostoma anisurum | Canada: Ontario: Thames River | morphological | ROM:Ich:BCF-0368-5 | BCF-0368-5 | EU524848 |
| Moxostoma anisurum | Canada: Ontario: Thames River | morphological | ROM:Ich:BCF-0368-4 | BCF-0368-4 | EU524849 |
| Moxostoma anisurum | Canada: Ontario: Thames River | morphological | ROM:Ich:BCF-0368-1 | BCF-0368-1 | EU524850 |
| Moxostoma anisurum | Canada: Quebec: Fleuve St-Laurent, riviere Richelieu | tissue | UOG:Bio:BCF-0096-2 | BCF-0096-2 | EU524851 |
| Moxostoma anisurum | Canada: Quebec: Fleuve St-Laurent, riviere Richelieu | tissue | UOG:Bio:BCF-0096-1 | BCF-0096-1 | EU524852 |
| Moxostoma anisurum | Canada: Quebec: Baie Missisquoi | tissue | UOG:Bio:BCF-0094-3 | BCF-0094-3 | EU524853 |
| Moxostoma anisurum | Canada: Quebec: Baie Missisquoi | tissue | UOG:Bio:BCF-0094-2 | BCF-0094-2 | EU524854 |
| Moxostoma anisurum | Canada: Quebec: Baie Missisquoi | tissue | UOG:Bio:BCF-0094-1 | BCF-0094-1 | EU524855 |
| Moxostoma carinatum | Canada: Quebec: Riviere Richelieu, Saint-Ours | tissue | UOG:Bio:BCF-0098-1 | BCF-0098-1 | EU524147 |
| Moxostoma carinatum | Canada: Quebec: Riviere Richelieu, Saint-Ours | tissue | UOG:Bio:BCF-0099-1 | BCF-0099-1 | EU524148 |
| Moxostoma carinatum | Canada: Ontario: Trent River | morphological | ROM:Ich:BCF-0531-1 | BCF-0531-1 | EU524856 |
| Moxostoma carinatum | Canada: Ontario: Trent River | morphological | ROM:Ich:BCF-0516-2 | BCF-0516-2 | EU524857 |
| Moxostoma carinatum | Canada: Ontario: Madawaska River | morphological | ROM:Ich:BCF-0445-1 | BCF-0445-1 | EU524858 |
| Moxostoma carinatum | Canada: Quebec: Fleuve St-Laurent, riviere Richelieu | tissue | UOG:Bio:BCF-0099-4 | BCF-0099-4 | EU524859 |
| Moxostoma carinatum | Canada: Quebec: Fleuve St-Laurent, riviere Richelieu | tissue | UOG:Bio:BCF-0099-2 | BCF-0099-2 | EU524860 |
| Moxostoma duquesnii | Canada: Ontario: Maitland River | morphological | ROM:Ich:BCF-0517-6 | BCF-0517-6 | EU524861 |
| Moxostoma duquesnii | Canada: Ontario: Maitland River | morphological | ROM:Ich:BCF-0517-5 | BCF-0517-5 | EU524862 |
| Moxostoma duquesnii | Canada: Ontario: Maitland River | morphological | ROM:Ich:BCF-0517-4 | BCF-0517-4 | EU524863 |
| Moxostoma duquesnii | Canada: Ontario: Fanshawe Lake | morphological | ROM:Ich:BCF-0517-3 | BCF-0517-3 | EU524864 |
| Moxostoma duquesnii | Canada: Ontario: Fanshawe Lake | morphological | ROM:Ich:BCF-0517-2 | BCF-0517-2 | EU524865 |
| Moxostoma duquesnii | Canada: Ontario: Fanshawe Lake | morphological | ROM:Ich:BCF-0517-1 | BCF-0517-1 | EU524866 |
| Moxostoma erythrurum | Canada: Ontario: Sydenham River | morphological | ROM:Ich:BCF-0416-6 | BCF-0416-6 | EU524867 |
| Moxostoma erythrurum | Canada: Ontario: Sydenham River | morphological | ROM:Ich:BCF-0416-5 | BCF-0416-5 | EU524868 |
| Moxostoma erythrurum | Canada: Ontario: Sydenham River | morphological | ROM:Ich:BCF-0416-4 | BCF-0416-4 | EU524869 |
| Moxostoma erythrurum | Canada: Ontario: Sydenham River | morphological | ROM:Ich:BCF-0416-3 | BCF-0416-3 | EU524870 |
| Moxostoma erythrurum | Canada: Ontario: Sydenham River | morphological | ROM:Ich:BCF-0416-2 | BCF-0416-2 | EU524871 |
| Moxostoma erythrurum | Canada: Ontario: Sydenham River | morphological | ROM:Ich:BCF-0416-1 | BCF-0416-1 | EU524872 |
| Moxostoma erythrurum | Canada: Ontario: Thames River | morphological | ROM:Ich:BCF-0397-4 | BCF-0397-4 | EU524873 |
| Moxostoma erythrurum | Canada: Ontario: Thames River | morphological | ROM:Ich:BCF-0397-3 | BCF-0397-3 | EU524874 |
| Moxostoma erythrurum | Canada: Ontario: Thames River | morphological | ROM:Ich:BCF-0397-2 | BCF-0397-2 | EU524875 |
| Moxostoma erythrurum | Canada: Ontario: Thames River | morphological | ROM:Ich:BCF-0397-1 | BCF-0397-1 | EU524876 |
| Moxostoma hubbsi | Canada: Quebec: Fleuve St-Laurent, riviere Richelieu | tissue | UOG:Bio:BCF-0617-15 | BCF-0617-15 | EU524877 |
| Moxostoma hubbsi | Canada: Quebec: Fleuve St-Laurent, riviere Richelieu | tissue | UOG:Bio:BCF-0617-14 | BCF-0617-14 | EU524878 |
| Moxostoma hubbsi | Canada: Quebec: Fleuve St-Laurent, riviere Richelieu | tissue | UOG:Bio:BCF-0617-13 | BCF-0617-13 | EU524879 |
| Moxostoma hubbsi | Canada: Quebec: Fleuve St-Laurent | tissue | UOG:Bio:BCF-0617-9 | BCF-0617-9 | EU524880 |
| Moxostoma hubbsi | Canada: Quebec: Fleuve St-Laurent | tissue | UOG:Bio:BCF-0617-8 | BCF-0617-8 | EU524881 |
| Moxostoma hubbsi | Canada: Quebec: Fleuve St-Laurent | tissue | UOG:Bio:BCF-0617-7 | BCF-0617-7 | EU524882 |
| Moxostoma hubbsi | Canada: Quebec: Fleuve St-Laurent | tissue | UOG:Bio:BCF-0617-6 | BCF-0617-6 | EU524883 |
| Moxostoma hubbsi | Canada: Quebec: Tadoussac | tissue | UOG:Bio:BCF-0617-5 | BCF-0617-5 | EU524884 |
| Moxostoma hubbsi | Canada: Quebec: Tadoussac | tissue | UOG:Bio:BCF-0617-4 | BCF-0617-4 | EU524885 |
| Moxostoma hubbsi | Canada: Quebec: Tadoussac | tissue | UOG:Bio:BCF-0617-3 | BCF-0617-3 | EU524886 |
| Moxostoma hubbsi | Canada: Quebec: Tadoussac | tissue | UOG:Bio:BCF-0617-2 | BCF-0617-2 | EU524887 |
| Moxostoma hubbsi | Canada: Quebec: Tadoussac | tissue | UOG:Bio:BCF-0617-1 | BCF-0617-1 | EU524888 |
| Moxostoma macrolepidotum | Canada: Quebec: Saint Lawrence River St-Nicolas | tissue | UOG:Bio:BCF-0091-2 | BCF-0091-2 | EU524149 |
| Moxostoma macrolepidotum | Canada: Ontario: Lake Erie | morphological | ROM:Ich:BCF-0532-5 | BCF-0532-5 | EU524889 |
| Moxostoma macrolepidotum | Canada: Ontario: Lake Erie | morphological | ROM:Ich:BCF-0532-4 | BCF-0532-4 | EU524890 |
| Moxostoma macrolepidotum | Canada: Ontario: Lake Erie | morphological | ROM:Ich:BCF-0532-3 | BCF-0532-3 | EU524891 |
| Moxostoma macrolepidotum | Canada: Ontario: Grand River | morphological | ROM:Ich:BCF-0490-1 | BCF-0490-1 | EU524892 |
| Moxostoma macrolepidotum | Canada: Quebec: Fleuve St-Laurent, riviere Richelieu | tissue | UOG:Bio:BCF-0093-3 | BCF-0093-3 | EU524893 |
| Moxostoma macrolepidotum | Canada: Quebec: Fleuve St-Laurent, riviere Richelieu | tissue | UOG:Bio:BCF-0093-2 | BCF-0093-2 | EU524894 |
| Moxostoma macrolepidotum | Canada: Quebec: Fleuve St-Laurent, riviere Richelieu | tissue | UOG:Bio:BCF-0093-1 | BCF-0093-1 | EU524895 |
| Moxostoma macrolepidotum | Canada: Quebec: Fleuve St-Laurent, lac St-Louis | morphological | ROM:Ich:BCF-0092-3 | BCF-0092-3 | EU524896 |
| Moxostoma macrolepidotum | Canada: Quebec: Fleuve St-Laurent, lac St-Louis | morphological | ROM:Ich:BCF-0092-2 | BCF-0092-2 | EU524897 |
| Moxostoma macrolepidotum | Canada: Quebec: Fleuve St-Laurent, lac St-Louis | morphological | ROM:Ich:BCF-0092-1 | BCF-0092-1 | EU524898 |
| Moxostoma macrolepidotum | Canada: Quebec: Fleuve St-Laurent, riviere St-Nicolas | tissue | UOG:Bio:BCF-0091-4 | BCF-0091-4 | EU524899 |
| Moxostoma macrolepidotum | Canada: Quebec: Fleuve St-Laurent, riviere St-Nicolas | tissue | UOG:Bio:BCF-0091-3 | BCF-0091-3 | EU524900 |
| Moxostoma macrolepidotum | Canada: Ontario: Thames River | morphological | ROM:Ich:BCF-0377-2 | BCF-0377-2 | EU524901 |
| Moxostoma macrolepidotum | Canada: Ontario: Thames River | morphological | ROM:Ich:BCF-0377-1 | BCF-0377-1 | EU524902 |
| Moxostoma macrolepidotum | Canada: Ontario: Trent River | morphological | ROM:Ich:BCF-0516-1 | BCF-0516-1 | EU524903 |
| Moxostoma valenciennesi | Canada: Quebec: Fleuve St-Laurent, riviere Richelieu | tissue | UOG:Bio:BCF-0097-1 | BCF-0097-1 | EU524150 |
| Moxostoma valenciennesi | Canada: Ontario: Lake Simcoe | morphological | ROM:Ich:BCF-0533-3 | BCF-0533-3 | EU524904 |
| Moxostoma valenciennesi | Canada: Ontario: Lake Simcoe | morphological | ROM:Ich:BCF-0533-1 | BCF-0533-1 | EU524905 |
| Moxostoma valenciennesi | Canada: Ontario: Crowe Lake | morphological | ROM:Ich:BCF-0525-4 | BCF-0525-4 | EU524906 |
| Moxostoma valenciennesi | Canada: Ontario: Crowe Lake | morphological | ROM:Ich:BCF-0525-3 | BCF-0525-3 | EU524907 |
| Moxostoma valenciennesi | Canada: Ontario: Crowe Lake | morphological | ROM:Ich:BCF-0525-2 | BCF-0525-2 | EU524908 |
| Moxostoma valenciennesi | Canada: Ontario: Crowe Lake | morphological | ROM:Ich:BCF-0525-1 | BCF-0525-1 | EU524909 |
| Moxostoma valenciennesi | Canada: Ontario: Fanshawe Lake | morphological | ROM:Ich:BCF-0520-2 | BCF-0520-2 | EU524910 |
| Moxostoma valenciennesi | Canada: Ontario: Fanshawe Lake | morphological | ROM:Ich:BCF-0520-1 | BCF-0520-1 | EU524911 |
| Moxostoma valenciennesi | Canada: Ontario: Grand River | morphological | ROM:Ich:BCF-0491-1 | BCF-0491-1 | EU524912 |
| Lepomis auritus | Canada: New Brunswick: Yoho Lake | tissue | UOG:Bio:BCF-0576-5 | BCF-0576-5 | EU524700 |
| Lepomis auritus | Canada: New Brunswick: Yoho Lake | tissue | UOG:Bio:BCF-0576-4 | BCF-0576-4 | EU524701 |
| Lepomis auritus | Canada: New Brunswick: Yoho Lake | tissue | UOG:Bio:BCF-0576-3 | BCF-0576-3 | EU524702 |
| Lepomis auritus | Canada: New Brunswick: Yoho Lake | tissue | UOG:Bio:BCF-0576-2 | BCF-0576-2 | EU524703 |
| Lepomis auritus | Canada: New Brunswick: Yoho Lake | tissue | UOG:Bio:BCF-0576-1 | BCF-0576-1 | EU524704 |
| Lepomis cyanellus | Canada: Ontario: Humber River | morphological | ROM:Ich:BCF-0477-1 | BCF-0477-1 | EU524705 |
| Lepomis cyanellus | Canada: Ontario: Fansher Creek | morphological | ROM:Ich:BCF-0414-8 | BCF-0414-8 | EU524706 |
| Lepomis cyanellus | Canada: Ontario: Fansher Creek | morphological | ROM:Ich:BCF-0414-7 | BCF-0414-7 | EU524707 |
| Lepomis cyanellus | Canada: Ontario: Fansher Creek | morphological | ROM:Ich:BCF-0414-6 | BCF-0414-6 | EU524708 |
| Lepomis cyanellus | Canada: Ontario: Fansher Creek | morphological | ROM:Ich:BCF-0414-5 | BCF-0414-5 | EU524709 |
| Lepomis cyanellus | Canada: Ontario: Fansher Creek | morphological | ROM:Ich:BCF-0414-4 | BCF-0414-4 | EU524710 |
| Lepomis cyanellus | Canada: Ontario: Fansher Creek | morphological | ROM:Ich:BCF-0414-3 | BCF-0414-3 | EU524711 |
| Lepomis cyanellus | Canada: Ontario: Fansher Creek | morphological | ROM:Ich:BCF-0414-2 | BCF-0414-2 | EU524712 |
| Lepomis cyanellus | Canada: Ontario: Sydenham River | morphological | ROM:Ich:BCF-0414-1 | BCF-0414-1 | EU524713 |
| Lepomis gibbosus | Canada: Quebec: Marais St-Eugene | morphological | ROM:Ich:BCF-0151-1 | BCF-0151-1 | EU524123 |
| Lepomis gibbosus | Canada: Ontario: Fleuve St-Laurent | tissue | UOG:Bio:BCF-0346-2 | BCF-0346-2 | EU524714 |
| Lepomis gibbosus | Canada: Ontario: Credit River | morphological | ROM:Ich:BCF-0442-3 | BCF-0442-3 | EU524715 |
| Lepomis gibbosus | Canada: Ontario: Credit River | morphological | ROM:Ich:BCF-0442-2 | BCF-0442-2 | EU524716 |
| Lepomis gibbosus | Canada: Ontario: Credit River | morphological | ROM:Ich:BCF-0442-1 | BCF-0442-1 | EU524717 |
| Lepomis gibbosus | Canada: Ontario: Lac Opinicon | morphological | ROM:Ich:BCF-0217-2 | BCF-0217-2 | EU524718 |
| Lepomis gibbosus | Canada: Ontario: Lac Opinicon | morphological | ROM:Ich:BCF-0217-1 | BCF-0217-1 | EU524719 |
| Lepomis gibbosus | Canada: Quebec: Fleuve St-Laurent, lac St-Pierre | tissue | UOG:Bio:BCF-0154-3 | BCF-0154-3 | EU524720 |
| Lepomis gibbosus | Canada: Quebec: Fleuve St-Laurent, lac St-Pierre | tissue | UOG:Bio:BCF-0154-2 | BCF-0154-2 | EU524721 |
| Lepomis gibbosus | Canada: Quebec: Fleuve St-Laurent, lac St-Pierre | tissue | UOG:Bio:BCF-0154-1 | BCF-0154-1 | EU524722 |
| Lepomis gibbosus | Canada: Quebec: Fleuve St-Laurent, lac St-Louis | morphological | ROM:Ich:BCF-0152-3 | BCF-0152-3 | EU524723 |
| Lepomis gibbosus | Canada: Quebec: Fleuve St-Laurent, lac St-Louis | morphological | ROM:Ich:BCF-0152-2 | BCF-0152-2 | EU524724 |
| Lepomis gibbosus | Canada: Quebec: Fleuve St-Laurent, lac St-Louis | morphological | ROM:Ich:BCF-0152-1 | BCF-0152-1 | EU524725 |
| Lepomis humilis | Canada: Ontario: Big Creek | morphological | ROM:Ich:BCF-0556-2 | BCF-0556-2 | EU524726 |
| Lepomis humilis | Canada: Ontario: Big Creek | morphological | ROM:Ich:BCF-0556-1 | BCF-0556-1 | EU524727 |
| Lepomis humilis | Canada: Ontario: Big Creek | morphological | ROM:Ich:BCF-0552-4 | BCF-0552-4 | EU524728 |
| Lepomis humilis | Canada: Ontario: Big Creek | morphological | ROM:Ich:BCF-0552-3 | BCF-0552-3 | EU524729 |
| Lepomis humilis | Canada: Ontario: Big Creek | morphological | ROM:Ich:BCF-0552-2 | BCF-0552-2 | EU524730 |
| Lepomis humilis | Canada: Ontario: Big Creek | morphological | ROM:Ich:BCF-0552-1 | BCF-0552-1 | EU524731 |
| Lepomis macrochirus | Canada: Ontario: Buckhorn Lake | morphological | ROM:Ich:BCF-0432-3 | BCF-0432-3 | EU524732 |
| Lepomis macrochirus | Canada: Ontario: Buckhorn Lake | morphological | ROM:Ich:BCF-0432-2 | BCF-0432-2 | EU524733 |
| Lepomis macrochirus | Canada: Ontario: Buckhorn Lake | morphological | ROM:Ich:BCF-0432-1 | BCF-0432-1 | EU524734 |
| Lepomis macrochirus | Canada: Ontario: Fleuve St-Laurent | morphological | ROM:Ich:BCF-0346-4 | BCF-0346-4 | EU524735 |
| Lepomis macrochirus | Canada: Ontario: Fleuve St-Laurent | morphological | ROM:Ich:BCF-0346-3 | BCF-0346-3 | EU524736 |
| Lepomis macrochirus | Canada: Ontario: Fleuve St-Laurent | morphological | ROM:Ich:BCF-0346-1 | BCF-0346-1 | EU524737 |
| Lepomis macrochirus | Canada: Ontario: Lac Opinicon | morphological | ROM:Ich:BCF-0213-4 | BCF-0213-4 | EU524738 |
| Lepomis macrochirus | Canada: Ontario: Lac Opinicon | morphological | ROM:Ich:BCF-0213-3 | BCF-0213-3 | EU524739 |
| Lepomis macrochirus | Canada: Ontario: Lac Opinicon | morphological | ROM:Ich:BCF-0213-2 | BCF-0213-2 | EU524740 |
| Lepomis macrochirus | Canada: Ontario: Buckhorn Lake | morphological | ROM:Ich:BCF-0432-4 | BCF-0432-4 | EU524741 |
| Lepomis megalotis | Canada: Quebec: Riviere Chateaugay | morphological | ROM:Ich:BCF-0150-1 | BCF-0150-1 | EU524124 |
| Lepomis megalotis | Canada: Quebec: Fleuve St-Laurent, riviere Chateaugay | morphological | ROM:Ich:BCF-0150-5 | BCF-0150-5 | EU524742 |
| Lepomis megalotis | Canada: Quebec: Fleuve St-Laurent, riviere Chateaugay | morphological | ROM:Ich:BCF-0150-4 | BCF-0150-4 | EU524743 |
| Lepomis megalotis | Canada: Quebec: Fleuve St-Laurent, riviere Chateaugay | morphological | ROM:Ich:BCF-0150-3 | BCF-0150-3 | EU524744 |
| Lepomis megalotis | Canada: Quebec: Fleuve St-Laurent, riviere Chateaugay | morphological | ROM:Ich:BCF-0150-2 | BCF-0150-2 | EU524745 |
| Ambloplites rupestris | Canada: Quebec: Fleuve St-Laurent, lac St-Paul | morphological | ROM:Ich:BCF-0159-1 | BCF-0159-1 | EU523904 |
| Ambloplites rupestris | Canada: Ontario: Grand River | morphological | ROM:Ich:BCF-0390-4 | BCF-0390-4 | EU524407 |
| Ambloplites rupestris | Canada: Ontario: Grand River | morphological | ROM:Ich:BCF-0390-3 | BCF-0390-3 | EU524408 |
| Ambloplites rupestris | Canada: Ontario: Grand River | morphological | ROM:Ich:BCF-0390-2 | BCF-0390-2 | EU524409 |
| Ambloplites rupestris | Canada: Ontario: Lk. Huron-Waubuno Ch. | morphological | ROM:Ich:BCF-0344-3 | BCF-0344-3 | EU524410 |
| Ambloplites rupestris | Canada: Ontario: Lac Opinicon | tissue | UOG:Bio:BCF-0220-2 | BCF-0220-2 | EU524411 |
| Ambloplites rupestris | Canada: Quebec: Fleuve St-Laurent, riviere Richelieu | tissue | UOG:Bio:BCF-0162-3 | BCF-0162-3 | EU524412 |
| Ambloplites rupestris | Canada: Quebec: Fleuve St-Laurent, riviere Richelieu | tissue | UOG:Bio:BCF-0162-2 | BCF-0162-2 | EU524413 |
| Ambloplites rupestris | Canada: Quebec: Fleuve St-Laurent, riviere Richelieu | tissue | UOG:Bio:BCF-0162-1 | BCF-0162-1 | EU524414 |
| Micropterus dolomieu | Canada: Quebec: Lac Saint-Louis (Fleuve Saint-Laurent) | morphological | ROM:Ich:BCF-0165-1 | BCF-0165-1 | EU524131 |
| Micropterus dolomieu | Canada: Ontario: Tea Creek | morphological | ROM:Ich:BCF-0444-2 | BCF-0444-2 | EU524810 |
| Micropterus dolomieu | Canada: Ontario: Tea Creek | morphological | ROM:Ich:BCF-0444-1 | BCF-0444-1 | EU524811 |
| Micropterus dolomieu | Canada: Ontario: Lac Opinicon | morphological | ROM:Ich:BCF-0215-3 | BCF-0215-3 | EU524812 |
| Micropterus dolomieu | Canada: Ontario: Lac Opinicon | morphological | ROM:Ich:BCF-0215-2 | BCF-0215-2 | EU524813 |
| Micropterus dolomieu | Canada: New Brunswick: Nosbonsing | morphological | ROM:Ich:BCF-0175-2 | BCF-0175-2 | EU524814 |
| Micropterus dolomieu | Canada: Quebec: Fleuve St-Laurent, Lac St-Pierre | morphological | ROM:Ich:BCF-0173-3 | BCF-0173-3 | EU524815 |
| Micropterus dolomieu | Canada: Ontario: Wanapitei River | morphological | ROM:Ich:BCF-0443-3 | BCF-0443-3 | EU524816 |
| Micropterus dolomieu | Canada: Ontario: Wanapitei River | morphological | ROM:Ich:BCF-0443-2 | BCF-0443-2 | EU524817 |
| Micropterus dolomieu | Canada: Ontario: Wanapitei River | morphological | ROM:Ich:BCF-0443-1 | BCF-0443-1 | EU524818 |
| Micropterus dolomieu | Canada: Ontario: Grand River | morphological | ROM:Ich:BCF-0395-2 | BCF-0395-2 | EU524819 |
| Micropterus dolomieu | Canada: Ontario: Grand River | morphological | ROM:Ich:BCF-0395-1 | BCF-0395-1 | EU524820 |
| Micropterus dolomieu | Canada: Quebec: lac Opasatica | morphological | ROM:Ich:BCF-0169-3 | BCF-0169-3 | EU524821 |
| Micropterus dolomieu | Canada: Quebec: lac Opasatica | morphological | ROM:Ich:BCF-0169-2 | BCF-0169-2 | EU524822 |
| Micropterus dolomieu | Canada: Quebec: lac Opasatica | morphological | ROM:Ich:BCF-0169-1 | BCF-0169-1 | EU524823 |
| Micropterus dolomieu | Canada: Quebec: Fleuve St-Laurent, riviere St-Nicolas | morphological | ROM:Ich:BCF-0168-3 | BCF-0168-3 | EU524824 |
| Micropterus dolomieu | Canada: Quebec: Fleuve St-Laurent, riviere St-Nicolas | morphological | ROM:Ich:BCF-0168-2 | BCF-0168-2 | EU524825 |
| Micropterus dolomieu | Canada: Quebec: Fleuve St-Laurent, riviere St-Nicolas | morphological | ROM:Ich:BCF-0168-1 | BCF-0168-1 | EU524826 |
| Micropterus dolomieu | Canada: Quebec: Fleuve St-Laurent, lac St-Louis | morphological | ROM:Ich:BCF-0165-3 | BCF-0165-3 | EU524827 |
| Micropterus dolomieu | Canada: Quebec: Fleuve St-Laurent, lac St-Louis | morphological | ROM:Ich:BCF-0165-2 | BCF-0165-2 | EU524828 |
| Micropterus salmoides | Canada: Ontario: Lac Opinicon | morphological | ROM:Ich:BCF-0215-1 | BCF-0215-1 | EU524132 |
| Micropterus salmoides | Canada: Ontario: Lake Simcoe | tissue | UOG:Bio:BCF-0546-1 | BCF-0546-1 | EU524829 |
| Micropterus salmoides | Canada: Ontario: Cedar Creek | tissue | UOG:Bio:BCF-0511-1 | BCF-0511-1 | EU524830 |
| Micropterus salmoides | Canada: Ontario: Lac Opinicon | tissue | UOG:Bio:BCF-0219-1 | BCF-0219-1 | EU524831 |
| Micropterus salmoides | Canada: Quebec: Fleuve St-Laurent, lac St-Paul | tissue | UOG:Bio:BCF-0177-2 | BCF-0177-2 | EU524832 |
| Micropterus salmoides | Canada: Quebec: Fleuve St-Laurent, lac St-Paul | tissue | UOG:Bio:BCF-0177-1 | BCF-0177-1 | EU524833 |
| Micropterus salmoides | Canada: Ontario: Tea Creek | morphological | ROM:Ich:BCF-0444-3 | BCF-0444-3 | EU524834 |
| Micropterus salmoides | Canada: Quebec: Fleuve St-Laurent, Lac St-Pierre | morphological | ROM:Ich:BCF-0173-2 | BCF-0173-2 | EU524835 |
| Micropterus salmoides | Canada: Quebec: Fleuve St-Laurent, Lac St-Pierre | morphological | ROM:Ich:BCF-0173-1 | BCF-0173-1 | EU524836 |
| Micropterus salmoides | Canada: Quebec: Fleuve St-Laurent, lac St-Louis | morphological | ROM:Ich:BCF-0171-3 | BCF-0171-3 | EU524837 |
| Micropterus salmoides | Canada: Quebec: Fleuve St-Laurent, lac St-Louis | morphological | ROM:Ich:BCF-0171-2 | BCF-0171-2 | EU524838 |
| Pomoxis annularis | Canada: Ontario: Sydenham River | morphological | ROM:Ich:BCF-0778-1 | BCF-0778-1 | EU524280 |
| Pomoxis annularis | Canada: Ontario: Welland river, city of welland | tissue | UOG:Bio:BCF-0721-5 | BCF-0721-5 | EU524281 |
| Pomoxis annularis | Canada: Ontario: Welland river, city of welland | tissue | UOG:Bio:BCF-0721-4 | BCF-0721-4 | EU524282 |
| Pomoxis annularis | Canada: Ontario: Welland river, city of welland | tissue | UOG:Bio:BCF-0721-2 | BCF-0721-2 | EU524283 |
| Pomoxis annularis | Canada: Ontario: Welland river, city of welland | tissue | UOG:Bio:BCF-0721-1 | BCF-0721-1 | EU524284 |
| Pomoxis annularis | Canada: Ontario: Lake Simcoe | morphological | ROM:Ich:BCF-0546-3 | BCF-0546-3 | EU525096 |
| Pomoxis annularis | Canada: Ontario: Sydenham River | morphological | ROM:Ich:BCF-0423-1 | BCF-0423-1 | EU525097 |
| Pomoxis nigromaculatus | Canada: Ontario: Welland river, city of welland | tissue | UOG:Bio:BCF-0720-3 | BCF-0720-3 | EU524285 |
| Pomoxis nigromaculatus | Canada: Ontario: Welland river, city of welland | tissue | UOG:Bio:BCF-0720-2 | BCF-0720-2 | EU524286 |
| Pomoxis nigromaculatus | Canada: Quebec: Lac Saint-Louis (Fleuve Saint-Laurent) | morphological | ROM:Ich:BCF-0176-1 | BCF-0176-1 | EU524287 |
| Pomoxis nigromaculatus | Canada: Quebec: Fleuve St-Laurent, lac St-Pierre | morphological | ROM:Ich:BCF-0546-3 | BCF-0546-3 | EU525098 |
| Pomoxis nigromaculatus | Canada: Ontario: Lake Simcoe | morphological | ROM:Ich:BCF-0546-2 | BCF-0546-2 | EU525099 |
| Pomoxis nigromaculatus | Canada: Ontario: Cedar Creek | morphological | ROM:Ich:BCF-0511-2 | BCF-0511-2 | EU525100 |
| Pomoxis nigromaculatus | Canada: Quebec: Fleuve St-Laurent, lac St-Louis | morphological | ROM:Ich:BCF-0176-3 | BCF-0176-3 | EU525101 |
| Pomoxis nigromaculatus | Canada: Quebec: Fleuve St-Laurent, lac St-Louis | morphological | ROM:Ich:BCF-0176-2 | BCF-0176-2 | EU525102 |
| Alosa aestivalis | Canada: Nova Scotia: Grand Lake | tissue | UOG:Bio:BCF-0885-5 | BCF-0885-5 | EU523894 |
| Alosa aestivalis | Canada: Nova Scotia: Grand Lake | tissue | UOG:Bio:BCF-0885-4 | BCF-0885-4 | EU523895 |
| Alosa aestivalis | Canada: Nova Scotia: Grand Lake | tissue | UOG:Bio:BCF-0885-3 | BCF-0885-3 | EU523896 |
| Alosa aestivalis | Canada: Nova Scotia: Grand Lake | tissue | UOG:Bio:BCF-0885-2 | BCF-0885-2 | EU523897 |
| Alosa aestivalis | Canada: Nova Scotia: Grand Lake | tissue | UOG:Bio:BCF-0885-1 | BCF-0885-1 | EU523898 |
| Alosa pseudoharengus | Canada: Quebec: Fleuve St-Laurent, lac St-Louis | morphological | ROM:Ich:BCF-0028-2 | BCF-0028-2 | EU523899 |
| Alosa pseudoharengus | Canada: Quebec: Fleuve St-Laurent, lac St-Louis | morphological | ROM:Ich:BCF-0028-1 | BCF-0028-1 | EU523900 |
| Alosa pseudoharengus | Canada: Ontario: St Clair River | morphological | ROM:Ich:BCF-0496-3 | BCF-0496-3 | EU524402 |
| Alosa pseudoharengus | Canada: Ontario: St Clair River | morphological | ROM:Ich:BCF-0496-1 | BCF-0496-1 | EU524403 |
| Alosa sapidissima | Canada: Quebec: Fleuve St-Laurent, lac St-Pierre | morphological | ROM:Ich:BCF-0200-3 | BCF-0200-3 | EU523901 |
| Alosa sapidissima | Canada: Quebec: Fleuve St-Laurent, lac St-Pierre | morphological | ROM:Ich:BCF-0200-2 | BCF-0200-2 | EU523902 |
| Alosa sapidissima | Canada: Quebec: Fleuve St-Laurent, lac St-Pierre | morphological | ROM:Ich:BCF-0200-1 | BCF-0200-1 | EU523903 |
| Alosa sapidissima | Canada: Quebec: Fleuve St-Laurent, riviere Richelieu | tissue | UOG:Bio:BCF-0026-2 | BCF-0026-2 | EU524404 |
| Alosa sapidissima | Canada: Quebec: Fleuve St-Laurent, riviere Richelieu | tissue | UOG:Bio:BCF-0026-1 | BCF-0026-1 | EU524405 |
| Alosa sapidissima | Canada: Quebec: Fleuve St-Laurent, riviere St-Nicolas | morphological | ROM:Ich:BCF-0023-3 | BCF-0023-3 | EU524406 |
| Dorosoma cepedianum | Canada: Ontario: Lake St Clair | morphological | ROM:Ich:BCF-0499-3 | BCF-0499-3 | EU524557 |
| Dorosoma cepedianum | Canada: Ontario: Lake St Clair | morphological | ROM:Ich:BCF-0499-2 | BCF-0499-2 | EU524558 |
| Dorosoma cepedianum | Canada: Ontario: Sydenham River | morphological | ROM:Ich:BCF-0404-4 | BCF-0404-4 | EU524559 |
| Dorosoma cepedianum | Canada: Ontario: Sydenham River | morphological | ROM:Ich:BCF-0404-2 | BCF-0404-2 | EU524560 |
| Dorosoma cepedianum | Canada: Ontario: Sydenham River | morphological | ROM:Ich:BCF-0404-1 | BCF-0404-1 | EU524561 |
| Dorosoma cepedianum | Canada: Ontario: Thames River | morphological | ROM:Ich:BCF-0380-5 | BCF-0380-5 | EU524562 |
| Dorosoma cepedianum | Canada: Ontario: Thames River | morphological | ROM:Ich:BCF-0380-4 | BCF-0380-4 | EU524563 |
| Dorosoma cepedianum | Canada: Ontario: Thames River | morphological | ROM:Ich:BCF-0380-3 | BCF-0380-3 | EU524564 |
| Dorosoma cepedianum | Canada: Ontario: Thames River | morphological | ROM:Ich:BCF-0380-2 | BCF-0380-2 | EU524565 |
| Dorosoma cepedianum | Canada: Ontario: Thames River | morphological | ROM:Ich:BCF-0380-1 | BCF-0380-1 | EU524566 |
| Cottus aleuticus | United States: Alaska: Bery bay, N Cr | tissue | UOG:Bio:BCF-0647-5 | BCF-0647-5 | EU523991 |
| Cottus aleuticus | United States: Alaska: Bery bay, N Cr | tissue | UOG:Bio:BCF-0647-4 | BCF-0647-4 | EU523992 |
| Cottus aleuticus | United States: Alaska: Bery bay, N Cr | tissue | UOG:Bio:BCF-0647-1 | BCF-0647-1 | EU523993 |
| Cottus asper | Canada: British Columbia: Chonat lake | tissue | UOG:Bio:BCF-0676-2 | BCF-0676-2 | EU523994 |
| Cottus asper | Canada: British Columbia: Chonat lake | tissue | UOG:Bio:BCF-0676-1 | BCF-0676-1 | EU523995 |
| Cottus asper | Canada: British Columbia: Sarita lake | tissue | UOG:Bio:BCF-0675-2 | BCF-0675-2 | EU523996 |
| Cottus asper | Canada: British Columbia: Sarita lake | tissue | UOG:Bio:BCF-0675-1 | BCF-0675-1 | EU523997 |
| Cottus bairdii | Canada: Manitoba: Birch River, Winnipeg River | tissue | UOG:Bio:BCF-0834-3 | BCF-0834-3 | EU522459 |
| Cottus bairdii | Canada: Manitoba: Birch River, Winnipeg River | tissue | UOG:Bio:BCF-0834-2 | BCF-0834-2 | EU522460 |
| Cottus bairdii | Canada: Manitoba: Birch River, Winnipeg River | tissue | UOG:Bio:BCF-0834-1 | BCF-0834-1 | EU522461 |
| Cottus bairdii | Canada: British Columbia: Otter creek | tissue | UOG:Bio:BCF-0672-4 | BCF-0672-4 | EU522455 |
| Cottus bairdii | Canada: British Columbia: Otter creek | tissue | UOG:Bio:BCF-0672-3 | BCF-0672-3 | EU522456 |
| Cottus bairdii | Canada: British Columbia: Otter creek | tissue | UOG:Bio:BCF-0672-2 | BCF-0672-2 | EU522457 |
| Cottus bairdii | Canada: British Columbia: Otter creek | tissue | UOG:Bio:BCF-0672-1 | BCF-0672-1 | EU522458 |
| Cottus bairdii | Canada: Quebec: Lac Saint-Louis (Fleuve Saint-Laurent) | morphological | ROM:Ich:BCF-0143-1 | BCF-0143-1 | EU523998 |
| Cottus bairdii | Canada: Ontario: Lk. Huron-Frazer Bay | morphological | ROM:Ich:BCF-0342-7 | BCF-0342-7 | EU524490 |
| Cottus bairdii | Canada: Ontario: Lk. Huron-Frazer Bay | morphological | ROM:Ich:BCF-0342-6 | BCF-0342-6 | EU524491 |
| Cottus bairdii | Canada: Ontario: Lk. Huron-Frazer Bay | morphological | ROM:Ich:BCF-0342-5 | BCF-0342-5 | EU524492 |
| Cottus bairdii | Canada: Ontario: Lk. Huron-Frazer Bay | morphological | ROM:Ich:BCF-0342-3 | BCF-0342-3 | EU524493 |
| Cottus bairdii | Canada: Ontario: Lk. Huron-Frazer Bay | morphological | ROM:Ich:BCF-0342-2 | BCF-0342-2 | EU524494 |
| Cottus bairdii | Canada: Ontario: Lk. Huron-Frazer Bay | morphological | ROM:Ich:BCF-0342-1 | BCF-0342-1 | EU524495 |
| Cottus bairdii | Canada: Ontario: Marden Creek | morphological | ROM:Ich:BCF-0427-4 | BCF-0427-4 | EU524496 |
| Cottus bairdii | Canada: Ontario: Marden Creek | morphological | ROM:Ich:BCF-0427-3 | BCF-0427-3 | EU524497 |
| Cottus bairdii | Canada: Ontario: Marden Creek | morphological | ROM:Ich:BCF-0427-2 | BCF-0427-2 | EU524498 |
| Cottus bairdii | Canada: Ontario: Marden Creek | morphological | ROM:Ich:BCF-0427-1 | BCF-0427-1 | EU524499 |
| Cottus bairdii | Canada: Quebec: Fleuve St-Laurent, riviere Cap-Rouge | morphological | ROM:Ich:BCF-0145-4 | BCF-0145-4 | EU524500 |
| Cottus bairdii | Canada: Quebec: Fleuve St-Laurent, riviere Cap-Rouge | morphological | ROM:Ich:BCF-0145-3 | BCF-0145-3 | EU524501 |
| Cottus bairdii | Canada: Quebec: Fleuve St-Laurent, riviere Cap-Rouge | morphological | ROM:Ich:BCF-0145-2 | BCF-0145-2 | EU524502 |
| Cottus bairdii | Canada: Quebec: Fleuve St-Laurent, riviere Cap-Rouge | morphological | ROM:Ich:BCF-0145-1 | BCF-0145-1 | EU524503 |
| Cottus bairdii | Canada: Quebec: Fleuve St-Laurent, lac St-Louis | morphological | ROM:Ich:BCF-0143-4 | BCF-0143-4 | EU524504 |
| Cottus bairdii | Canada: Quebec: Fleuve St-Laurent, lac St-Louis | morphological | ROM:Ich:BCF-0143-3 | BCF-0143-3 | EU524505 |
| Cottus bairdii | Canada: Quebec: Fleuve St-Laurent, lac St-Louis | morphological | ROM:Ich:BCF-0143-2 | BCF-0143-2 | EU524506 |
| Cottus cognatus | Canada: Quebec: Riviere Matane | morphological | ROM:Ich:BCF-0140-1 | BCF-0140-1 | EU523999 |
| Cottus cognatus | Canada: New Brunswick: Mc Quarrie Brook | tissue | UOG:Bio:BCF-0577-5 | BCF-0577-5 | EU524507 |
| Cottus cognatus | Canada: New Brunswick: Mc Quarrie Brook | tissue | UOG:Bio:BCF-0577-3 | BCF-0577-3 | EU524508 |
| Cottus cognatus | Canada: New Brunswick: Mc Quarrie Brook | tissue | UOG:Bio:BCF-0577-2 | BCF-0577-2 | EU524509 |
| Cottus cognatus | Canada: New Brunswick: Mc Quarrie Brook | tissue | UOG:Bio:BCF-0577-1 | BCF-0577-1 | EU524510 |
| Cottus cognatus | Canada: Ontario: Wilmot Creek | morphological | ROM:Ich:BCF-0456-5 | BCF-0456-5 | EU524511 |
| Cottus cognatus | Canada: Ontario: Wilmot Creek | morphological | ROM:Ich:BCF-0456-4 | BCF-0456-4 | EU524512 |
| Cottus cognatus | Canada: Ontario: Wilmot Creek | morphological | ROM:Ich:BCF-0456-3 | BCF-0456-3 | EU524513 |
| Cottus cognatus | Canada: Ontario: Wilmot Creek | morphological | ROM:Ich:BCF-0456-2 | BCF-0456-2 | EU524514 |
| Cottus cognatus | Canada: Quebec: Fleuve St-Laurent, riviere Matane | morphological | ROM:Ich:BCF-0140-5 | BCF-0140-5 | EU524515 |
| Cottus cognatus | Canada: Quebec: Fleuve St-Laurent, riviere Matane | morphological | ROM:Ich:BCF-0140-4 | BCF-0140-4 | EU524516 |
| Cottus cognatus | Canada: Quebec: Fleuve St-Laurent, riviere Matane | morphological | ROM:Ich:BCF-0140-3 | BCF-0140-3 | EU524517 |
| Cottus cognatus | Canada: Quebec: Fleuve St-Laurent, riviere Matane | morphological | ROM:Ich:BCF-0140-2 | BCF-0140-2 | EU524518 |
| Cottus cognatus | Canada: Quebec: Fleuve St-Laurent, riviere Malbaie | morphological | ROM:Ich:BCF-0144-2 | BCF-0144-2 | EU524519 |
| Cottus cognatus | Canada: Quebec: Fleuve St-Laurent, riviere Malbaie | morphological | ROM:Ich:BCF-0144-1 | BCF-0144-1 | EU524520 |
| Cottus rhotheus | Canada: British Columbia: Pass creek | tissue | UOG:Bio:BCF-0674-1 | BCF-0674-1 | EU524000 |
| Cottus ricei | Canada: Quebec: Saint Laurent River | morphological | ROM:Ich:BCF-0884-4 | BCF-0884-4 | EU522462 |
| Cottus ricei | Canada: Quebec: Saint Laurent River | morphological | ROM:Ich:BCF-0884-3 | BCF-0884-3 | EU522463 |
| Cottus ricei | Canada: Quebec: Saint Laurent River | morphological | ROM:Ich:BCF-0884-1 | BCF-0884-1 | EU524001 |
| Cottus ricei | Canada: Ontario: Lk. Superior-Whitefish Bay | morphological | ROM:Ich:BCF-0336-1 | BCF-0336-1 | EU524521 |
| Myoxocephalus quadricornis | Canada: Ontario: James Bay | morphological | ROM:Ich:BCF-0398-1 | BCF-0398-1 | EU524913 |
| Myoxocephalus thompsonii | Canada: Ontario: Lk. Superior-Whitefish Bay | morphological | ROM:Ich:BCF-0337-2 | BCF-0337-2 | EU524914 |
| Myoxocephalus thompsonii | Canada: Ontario: Lk. Superior-Whitefish Bay | morphological | ROM:Ich:BCF-0337-1 | BCF-0337-1 | EU524915 |
| Myoxocephalus thompsonii | Canada: Ontario: Lk. Huron-Meldrum Bay | morphological | ROM:Ich:BCF-0333-3 | BCF-0333-3 | EU524916 |
| Myoxocephalus thompsonii | Canada: Ontario: Lk. Huron-Meldrum Bay | morphological | ROM:Ich:BCF-0333-2 | BCF-0333-2 | EU524917 |
| Myoxocephalus thompsonii | Canada: Ontario: Lk. Huron-Meldrum Bay | morphological | ROM:Ich:BCF-0333-1 | BCF-0333-1 | EU524918 |
| Acrocheilus alutaceus | Canada: British Columbia: Kettle river | tissue | UOG:Bio:BCF-0667-3 | BCF-0667-3 | EU523892 |
| Acrocheilus alutaceus | Canada: British Columbia: Kettle river | tissue | UOG:Bio:BCF-0667-2 | BCF-0667-2 | EU523893 |
| Campostoma anomalum | Canada: Ontario: Cedar Creek | morphological | ROM:Ich:BCF-0549-1 | BCF-0549-1 | EU524447 |
| Carassius auratus | Canada: Ontario: Big Creek | morphological | ROM:Ich:BCF-0550-3 | BCF-0550-3 | EU524448 |
| Carassius auratus | Canada: Ontario: Big Creek | morphological | ROM:Ich:BCF-0550-2 | BCF-0550-2 | EU524449 |
| Carassius auratus | Canada: Ontario: Big Creek | morphological | ROM:Ich:BCF-0550-1 | BCF-0550-1 | EU524450 |
| Clinostomus elongatus | Canada: Ontario: Silver Creek | morphological | ROM:Ich:BCF-0529-1 | BCF-0529-1 | EU524485 |
| Clinostomus elongatus | Canada: Ontario: Fourteen Mile Creek | tissue | UOG:Bio:BCF-0524-5 | BCF-0524-5 | EU524486 |
| Clinostomus elongatus | Canada: : | tissue | UOG:Bio:BCF-0524-2 | BCF-0524-2 | EU524487 |
| Clinostomus elongatus | Canada: : | tissue | UOG:Bio:BCF-0524-1 | BCF-0524-1 | EU524488 |
| Couesius plumbeus | Canada: Quebec: Reserve Rouge-Matawin, lac Dalpec | morphological | ROM:Ich:BCF-0051-1 | BCF-0051-1 | EU524002 |
| Couesius plumbeus | Canada: New Brunswick: Gapetown | morphological | ROM:Ich:BCF-0586-1 | BCF-0586-1 | EU524522 |
| Couesius plumbeus | Canada: Ontario: Sheridan Creek | morphological | ROM:Ich:BCF-0429-1 | BCF-0429-1 | EU524523 |
| Couesius plumbeus | Canada: Ontario: Lk. Huron-McGregor Bay | morphological | ROM:Ich:BCF-0343-1 | BCF-0343-1 | EU524524 |
| Couesius plumbeus | Canada: Quebec: Reserve Rouge-Matawin, lac Dalpec | tissue | UOG:Bio:BCF-0051-8 | BCF-0051-8 | EU524525 |
| Couesius plumbeus | Canada: Quebec: Reserve Rouge-Matawin, lac Dalpec | morphological | ROM:Ich:BCF-0051-7 | BCF-0051-7 | EU524526 |
| Couesius plumbeus | Canada: Quebec: Reserve Rouge-Matawin, lac Dalpec | morphological | ROM:Ich:BCF-0051-6 | BCF-0051-6 | EU524527 |
| Couesius plumbeus | Canada: Quebec: Reserve Rouge-Matawin, lac Dalpec | morphological | ROM:Ich:BCF-0051-5 | BCF-0051-5 | EU524528 |
| Couesius plumbeus | Canada: Quebec: Reserve Rouge-Matawin, lac Dalpec | morphological | ROM:Ich:BCF-0051-4 | BCF-0051-4 | EU524529 |
| Couesius plumbeus | Canada: Quebec: Reserve Rouge-Matawin, lac Dalpec | morphological | ROM:Ich:BCF-0051-3 | BCF-0051-3 | EU524530 |
| Couesius plumbeus | Canada: Quebec: Reserve Rouge-Matawin, lac Dalpec | morphological | ROM:Ich:BCF-0051-2 | BCF-0051-2 | EU524531 |
| Cyprinella spiloptera | Canada: Quebec: Fleuve Saint-Laurent, Lac St-Pierre | morphological | ROM:Ich:BCF-0070-1 | BCF-0070-1 | EU524004 |
| Cyprinella spiloptera | Canada: Quebec: Fleuve Saint-Laurent, Lac St-Pierre | morphological | ROM:Ich:BCF-0070-2 | BCF-0070-2 | EU524005 |
| Cyprinella spiloptera | Canada: Ontario: Grand River | morphological | ROM:Ich:BCF-0379-3 | BCF-0379-3 | EU524539 |
| Cyprinella spiloptera | Canada: Ontario: Grand River | morphological | ROM:Ich:BCF-0379-2 | BCF-0379-2 | EU524540 |
| Cyprinella spiloptera | Canada: Ontario: Grand River | morphological | ROM:Ich:BCF-0379-1 | BCF-0379-1 | EU524541 |
| Cyprinella spiloptera | Canada: Ontario: Thames River | morphological | ROM:Ich:BCF-0369-4 | BCF-0369-4 | EU524542 |
| Cyprinella spiloptera | Canada: Ontario: Thames River | morphological | ROM:Ich:BCF-0369-3 | BCF-0369-3 | EU524543 |
| Cyprinella spiloptera | Canada: Ontario: Thames River | morphological | ROM:Ich:BCF-0369-2 | BCF-0369-2 | EU524544 |
| Cyprinella spiloptera | Canada: Ontario: Thames River | morphological | ROM:Ich:BCF-0369-1 | BCF-0369-1 | EU524545 |
| Cyprinella spiloptera | Canada: Quebec: Fleuve St-Laurent, riviere St-Jean | morphological | ROM:Ich:BCF-0289-1 | BCF-0289-1 | EU524546 |
| Cyprinella spiloptera | Canada: Quebec: Fleuve St-Laurent, Lac St-Pierre | morphological | ROM:Ich:BCF-0070-4 | BCF-0070-4 | EU524547 |
| Cyprinella spiloptera | Canada: Quebec: Fleuve St-Laurent, Lac St-Pierre | morphological | ROM:Ich:BCF-0070-3 | BCF-0070-3 | EU524548 |
| Cyprinus carpio | Canada: Quebec: Lac Saint-Pierre, Pointe Yamachiche | tissue | UOG:Bio:BCF-0048-1 | BCF-0048-1 | EU524006 |
| Cyprinus carpio | Canada: Quebec: Fleuve St-Laurent, lac St-Paul | morphological | ROM:Ich:BCF-0049-4 | BCF-0049-4 | EU524549 |
| Cyprinus carpio | Canada: Quebec: Fleuve St-Laurent, lac St-Paul | morphological | ROM:Ich:BCF-0049-3 | BCF-0049-3 | EU524550 |
| Cyprinus carpio | Canada: Quebec: Fleuve St-Laurent, lac St-Paul | morphological | ROM:Ich:BCF-0049-2 | BCF-0049-2 | EU524551 |
| Cyprinus carpio | Canada: Quebec: Fleuve St-Laurent, lac St-Paul | morphological | ROM:Ich:BCF-0049-1 | BCF-0049-1 | EU524552 |
| Cyprinus carpio | Canada: Quebec: Fleuve St-Laurent, lac St-Pierre | tissue | UOG:Bio:BCF-0048-3 | BCF-0048-3 | EU524553 |
| Cyprinus carpio | Canada: Quebec: Baie Missisquoi | tissue | UOG:Bio:BCF-0047-3 | BCF-0047-3 | EU524554 |
| Cyprinus carpio | Canada: Quebec: Baie Missisquoi | tissue | UOG:Bio:BCF-0047-2 | BCF-0047-2 | EU524555 |
| Cyprinus carpio | Canada: Quebec: Baie Missisquoi | tissue | UOG:Bio:BCF-0047-1 | BCF-0047-1 | EU524556 |
| Exoglossum maxillingua | Canada: Quebec: Riviere Becancour | morphological | ROM:Ich:BCF-0052-1 | BCF-0052-1 | EU524057 |
| Exoglossum maxillingua | Canada: Quebec: Fleuve St-Laurent, riviere Becancour | morphological | ROM:Ich:BCF-0052-5 | BCF-0052-5 | EU524613 |
| Exoglossum maxillingua | Canada: Quebec: Fleuve St-Laurent, riviere Becancour | morphological | ROM:Ich:BCF-0052-4 | BCF-0052-4 | EU524614 |
| Exoglossum maxillingua | Canada: Quebec: Fleuve St-Laurent, riviere Becancour | morphological | ROM:Ich:BCF-0052-3 | BCF-0052-3 | EU524615 |
| Exoglossum maxillingua | Canada: Quebec: Fleuve St-Laurent, riviere Becancour | morphological | ROM:Ich:BCF-0052-2 | BCF-0052-2 | EU524616 |
| Hybognathus argyritis | United States: Missouri: Milk River | tissue | UOG:Bio:BCF-0841-7 | BCF-0841-7 | EU524069 |
| Hybognathus argyritis | United States: Missouri: Milk River | tissue | UOG:Bio:BCF-0841-6 | BCF-0841-6 | EU524070 |
| Hybognathus argyritis | United States: Missouri: Milk River | tissue | UOG:Bio:BCF-0841-5 | BCF-0841-5 | EU524071 |
| Hybognathus argyritis | United States: Missouri: Milk River | tissue | UOG:Bio:BCF-0841-4 | BCF-0841-4 | EU524072 |
| Hybognathus argyritis | United States: Missouri: Milk River | tissue | UOG:Bio:BCF-0841-3 | BCF-0841-3 | EU524073 |
| Hybognathus argyritis | United States: Missouri: Milk River | tissue | UOG:Bio:BCF-0841-2 | BCF-0841-2 | EU524074 |
| Hybognathus argyritis | United States: Missouri: Milk River | tissue | UOG:Bio:BCF-0841-1 | BCF-0841-1 | EU522464 |
| Hybognathus hankinsoni | Canada: British Columbia: Bog pond | morphological | ROM:Ich:BCF-0698-5 | BCF-0698-5 | EU524075 |
| Hybognathus hankinsoni | Canada: British Columbia: Bog pond | morphological | ROM:Ich:BCF-0698-4 | BCF-0698-4 | EU524076 |
| Hybognathus hankinsoni | Canada: British Columbia: Bog pond | morphological | ROM:Ich:BCF-0698-3 | BCF-0698-3 | EU524077 |
| Hybognathus hankinsoni | Canada: British Columbia: Bog pond | morphological | ROM:Ich:BCF-0698-2 | BCF-0698-2 | EU524078 |
| Hybognathus hankinsoni | United States: Missouri: Rock creek | tissue | UOG:Bio:BCF-0842-2 | BCF-0842-2 | EU524079 |
| Hybognathus hankinsoni | United States: Missouri: Rock creek | tissue | UOG:Bio:BCF-0842-1 | BCF-0842-1 | EU524080 |
| Hybognathus hankinsoni | Canada: Quebec: Ruisseau Charette | morphological | ROM:Ich:BCF-0053-2 | BCF-0053-2 | EU524081 |
| Hybognathus placitus | United States: Missouri: Rock creek | tissue | UOG:Bio:BCF-0844-3 | BCF-0844-3 | EU524082 |
| Hybognathus placitus | United States: Missouri: Rock creek | tissue | UOG:Bio:BCF-0844-2 | BCF-0844-2 | EU524083 |
| Hybognathus placitus | United States: Missouri: Rock creek | tissue | UOG:Bio:BCF-0844-1 | BCF-0844-1 | EU524084 |
| Hybognathus regius | Canada: Quebec: Lac Saint-Pierre, Pointe Yamachiche | morphological | ROM:Ich:BCF-0054-1 | BCF-0054-1 | EU524085 |
| Hybognathus regius | Canada: Quebec: Fleuve Saint-Laurent, Lac St-Pierre | morphological | ROM:Ich:BCF-0055-1 | BCF-0055-1 | EU524086 |
| Hybognathus regius | Canada: Quebec: Fleuve St-Laurent, lac St-Pierre | morphological | ROM:Ich:BCF-0054-7 | BCF-0054-7 | EU524662 |
| Hybognathus regius | Canada: Quebec: Fleuve St-Laurent, lac St-Pierre | morphological | ROM:Ich:BCF-0054-6 | BCF-0054-6 | EU524663 |
| Hybognathus regius | Canada: Quebec: Fleuve St-Laurent, lac St-Pierre | morphological | ROM:Ich:BCF-0054-4 | BCF-0054-4 | EU524664 |
| Hybognathus regius | Canada: Quebec: Fleuve St-Laurent, lac St-Pierre | morphological | ROM:Ich:BCF-0054-3 | BCF-0054-3 | EU524665 |
| Hybognathus regius | Canada: Quebec: Fleuve St-Laurent, lac St-Pierre | morphological | ROM:Ich:BCF-0054-2 | BCF-0054-2 | EU524666 |
| Luxilus chrysocephalus | Canada: Ontario: Grand River | morphological | ROM:Ich:BCF-0489-1 | BCF-0489-1 | EU524758 |
| Luxilus chrysocephalus | Canada: Ontario: Sydenham River | morphological | ROM:Ich:BCF-0406-5 | BCF-0406-5 | EU524759 |
| Luxilus chrysocephalus | Canada: Ontario: Sydenham River | morphological | ROM:Ich:BCF-0406-4 | BCF-0406-4 | EU524760 |
| Luxilus chrysocephalus | Canada: Ontario: Sydenham River | morphological | ROM:Ich:BCF-0406-3 | BCF-0406-3 | EU524761 |
| Luxilus chrysocephalus | Canada: Ontario: Grand River | morphological | ROM:Ich:BCF-0382-4 | BCF-0382-4 | EU524762 |
| Luxilus chrysocephalus | Canada: Ontario: Grand River | morphological | ROM:Ich:BCF-0382-2 | BCF-0382-2 | EU524763 |
| Luxilus chrysocephalus | Canada: Ontario: Grand River | morphological | ROM:Ich:BCF-0382-1 | BCF-0382-1 | EU524764 |
| Luxilus cornutus | Canada: Quebec: Ruisseau Charette | morphological | ROM:Ich:BCF-0053-1 | BCF-0053-1 | EU524126 |
| Luxilus cornutus | Canada: Quebec: Lac aux Sables | morphological | ROM:Ich:BCF-0064-1 | BCF-0064-1 | EU524127 |
| Luxilus cornutus | Canada: Quebec: Fleuve St-Laurent, ruisseau Charette | morphological | ROM:Ich:BCF-0053-3 | BCF-0053-3 | EU524765 |
| Luxilus cornutus | Canada: New Brunswick: Mc Quarrie Brook | tissue | UOG:Bio:BCF-0584-3 | BCF-0584-3 | EU524766 |
| Luxilus cornutus | Canada: New Brunswick: Mc Quarrie Brook | tissue | UOG:Bio:BCF-0584-2 | BCF-0584-2 | EU524767 |
| Luxilus cornutus | Canada: Ontario: Sydenham River | morphological | ROM:Ich:BCF-0407-3 | BCF-0407-3 | EU524768 |
| Luxilus cornutus | Canada: Ontario: Sydenham River | morphological | ROM:Ich:BCF-0407-2 | BCF-0407-2 | EU524769 |
| Luxilus cornutus | Canada: Ontario: Sydenham River | morphological | ROM:Ich:BCF-0407-1 | BCF-0407-1 | EU524770 |
| Luxilus cornutus | Canada: Quebec: Fleuve St-Laurent, riviere St-Charles | morphological | ROM:Ich:BCF-0233-3 | BCF-0233-3 | EU524771 |
| Luxilus cornutus | Canada: Quebec: Fleuve St-Laurent, riviere St-Charles | morphological | ROM:Ich:BCF-0233-2 | BCF-0233-2 | EU524772 |
| Luxilus cornutus | Canada: Quebec: Fleuve St-Laurent, riviere St-Charles | morphological | ROM:Ich:BCF-0233-1 | BCF-0233-1 | EU524773 |
| Luxilus cornutus | Canada: Quebec: Fleuve St-Laurent, riviere Cap-Rouge | tissue | UOG:Bio:BCF-0230-4 | BCF-0230-4 | EU524774 |
| Luxilus cornutus | Canada: Quebec: Fleuve St-Laurent, riviere Cap-Rouge | morphological | ROM:Ich:BCF-0230-2 | BCF-0230-2 | EU524775 |
| Luxilus cornutus | Canada: Quebec: lac aux Sables | morphological | ROM:Ich:BCF-0064-2 | BCF-0064-2 | EU524776 |
| Luxilus cornutus | Canada: Ontario: Sydenham River | morphological | ROM:Ich:BCF-0406-6 | BCF-0406-6 | EU524777 |
| Luxilus cornutus | Canada: Ontario: Sydenham River | morphological | ROM:Ich:BCF-0406-2 | BCF-0406-2 | EU524778 |
| Luxilus cornutus | Canada: Ontario: Sydenham River | morphological | ROM:Ich:BCF-0406-1 | BCF-0406-1 | EU524779 |
| Luxilus cornutus | Canada: Ontario: Grand River | morphological | ROM:Ich:BCF-0382-6 | BCF-0382-6 | EU524780 |
| Luxilus cornutus | Canada: Ontario: Grand River | morphological | ROM:Ich:BCF-0382-5 | BCF-0382-5 | EU524781 |
| Luxilus cornutus | Canada: Ontario: Grand River | morphological | ROM:Ich:BCF-0382-3 | BCF-0382-3 | EU524782 |
| Luxilus cornutus | Canada: Quebec: Fleuve St-Laurent, ruisseau Charette | morphological | ROM:Ich:BCF-0053-7 | BCF-0053-7 | EU524783 |
| Luxilus cornutus | Canada: Quebec: Fleuve St-Laurent, ruisseau Charette | morphological | ROM:Ich:BCF-0053-6 | BCF-0053-6 | EU524784 |
| Luxilus cornutus | Canada: Quebec: Fleuve St-Laurent, ruisseau Charette | morphological | ROM:Ich:BCF-0053-5 | BCF-0053-5 | EU524785 |
| Luxilus cornutus | Canada: Quebec: Fleuve St-Laurent, ruisseau Charette | morphological | ROM:Ich:BCF-0053-4 | BCF-0053-4 | EU524786 |
| Lythrurus umbratilis | Canada: Ontario: Sydenham River | morphological | ROM:Ich:BCF-0408-9 | BCF-0408-9 | EU524787 |
| Lythrurus umbratilis | Canada: Ontario: Sydenham River | morphological | ROM:Ich:BCF-0408-8 | BCF-0408-8 | EU524788 |
| Lythrurus umbratilis | Canada: Ontario: Sydenham River | morphological | ROM:Ich:BCF-0408-7 | BCF-0408-7 | EU524789 |
| Lythrurus umbratilis | Canada: Ontario: Sydenham River | morphological | ROM:Ich:BCF-0408-5 | BCF-0408-5 | EU524790 |
| Lythrurus umbratilis | Canada: Ontario: Sydenham River | morphological | ROM:Ich:BCF-0408-4 | BCF-0408-4 | EU524791 |
| Lythrurus umbratilis | Canada: Ontario: Sydenham River | morphological | ROM:Ich:BCF-0408-10 | BCF-0408-10 | EU524792 |
| Lythrurus umbratilis | Canada: Ontario: Sydenham River | morphological | ROM:Ich:BCF-0408-1 | BCF-0408-1 | EU524793 |
| Macrhybopsis storeriana | Canada: Ontario: Lake Erie | morphological | ROM:Ich:BCF-0553-8 | BCF-0553-8 | EU524794 |
| Macrhybopsis storeriana | Canada: Ontario: Lake Erie | morphological | ROM:Ich:BCF-0553-7 | BCF-0553-7 | EU524795 |
| Macrhybopsis storeriana | Canada: Ontario: Lake Erie | morphological | ROM:Ich:BCF-0553-6 | BCF-0553-6 | EU524796 |
| Macrhybopsis storeriana | Canada: Ontario: Lake Erie | morphological | ROM:Ich:BCF-0553-5 | BCF-0553-5 | EU524797 |
| Macrhybopsis storeriana | Canada: Ontario: Lake Erie | morphological | ROM:Ich:BCF-0553-4 | BCF-0553-4 | EU524798 |
| Macrhybopsis storeriana | Canada: Ontario: Lake Erie | morphological | ROM:Ich:BCF-0553-3 | BCF-0553-3 | EU524799 |
| Macrhybopsis storeriana | Canada: Ontario: Lake Erie | morphological | ROM:Ich:BCF-0553-2 | BCF-0553-2 | EU524800 |
| Macrhybopsis storeriana | Canada: Ontario: Lake Erie | morphological | ROM:Ich:BCF-0553-1 | BCF-0553-1 | EU524801 |
| Margariscus margarita | Canada: Quebec: Lac Ecarte | morphological | ROM:Ich:BCF-0089-1 | BCF-0089-1 | EU524128 |
| Margariscus margarita | Canada: Ontario: Wetland 14 | tissue | UOG:Bio:BCF-0458-2 | BCF-0458-2 | EU524802 |
| Margariscus margarita | Canada: Quebec: Fleuve St-Laurent, riviere Richelieu | morphological | ROM:Ich:BCF-0090-4 | BCF-0090-4 | EU524803 |
| Margariscus margarita | Canada: Quebec: Fleuve St-Laurent, riviere Richelieu | morphological | ROM:Ich:BCF-0090-2 | BCF-0090-2 | EU524804 |
| Margariscus margarita | Canada: Quebec: Fleuve St-Laurent, riviere Richelieu | morphological | ROM:Ich:BCF-0090-1 | BCF-0090-1 | EU524805 |
| Margariscus margarita | Canada: Quebec: lac Ecarte | morphological | ROM:Ich:BCF-0089-13 | BCF-0089-13 | EU524806 |
| Margariscus margarita | Canada: Quebec: lac Ecarte | morphological | ROM:Ich:BCF-0089-12 | BCF-0089-12 | EU524807 |
| Margariscus margarita | Canada: Quebec: lac Ecarte | morphological | ROM:Ich:BCF-0089-11 | BCF-0089-11 | EU524808 |
| Margariscus margarita | Canada: Quebec: lac Ecarte | morphological | ROM:Ich:BCF-0089-10 | BCF-0089-10 | EU524809 |
| Mylocheilus caurinus | Canada: British Columbia: North Thompson | tissue | UOG:Bio:BCF-0684-2 | BCF-0684-2 | EU524151 |
| Mylocheilus caurinus | Canada: British Columbia: Chebalis river | tissue | UOG:Bio:BCF-0660-3 | BCF-0660-3 | EU524152 |
| Mylocheilus caurinus | Canada: British Columbia: Chebalis river | tissue | UOG:Bio:BCF-0660-2 | BCF-0660-2 | EU524153 |
| Nocomis biguttatus | Canada: Manitoba: Birch River, Winnipeg River | tissue | UOG:Bio:BCF-0833-4 | BCF-0833-4 | EU524157 |
| Nocomis biguttatus | Canada: Manitoba: Birch River, Winnipeg River | tissue | UOG:Bio:BCF-0833-3 | BCF-0833-3 | EU524158 |
| Nocomis biguttatus | Canada: Manitoba: Birch River, Winnipeg River | tissue | UOG:Bio:BCF-0833-2 | BCF-0833-2 | EU524159 |
| Nocomis biguttatus | Canada: Manitoba: Birch River, Winnipeg River | tissue | UOG:Bio:BCF-0833-1 | BCF-0833-1 | EU524160 |
| Nocomis biguttatus | Canada: Ontario: St Clair River | morphological | ROM:Ich:BCF-0563-2 | BCF-0563-2 | EU524921 |
| Nocomis biguttatus | Canada: Ontario: St Clair River | morphological | ROM:Ich:BCF-0563-1 | BCF-0563-1 | EU524922 |
| Nocomis biguttatus | Canada: Ontario: Fairchild Creek Tributary | morphological | ROM:Ich:BCF-0506-1 | BCF-0506-1 | EU524923 |
| Nocomis micropogon | Canada: Ontario: Humber River | morphological | ROM:Ich:BCF-0479-1 | BCF-0479-1 | EU524924 |
| Nocomis micropogon | Canada: Ontario: Grand River | morphological | ROM:Ich:BCF-0383-1 | BCF-0383-1 | EU524925 |
| Notemigonus crysoleucas | Canada: Quebec: Marais St-Eugene | morphological | ROM:Ich:BCF-0058-1 | BCF-0058-1 | EU524161 |
| Notemigonus crysoleucas | Canada: New Brunswick: Mc Quarrie Brook | morphological | ROM:Ich:BCF-0583-4 | BCF-0583-4 | EU524926 |
| Notemigonus crysoleucas | Canada: New Brunswick: Mc Quarrie Brook | morphological | ROM:Ich:BCF-0583-3 | BCF-0583-3 | EU524927 |
| Notemigonus crysoleucas | Canada: New Brunswick: Mc Quarrie Brook | morphological | ROM:Ich:BCF-0583-2 | BCF-0583-2 | EU524928 |
| Notemigonus crysoleucas | Canada: New Brunswick: Mc Quarrie Brook | morphological | ROM:Ich:BCF-0583-1 | BCF-0583-1 | EU524929 |
| Notemigonus crysoleucas | Canada: Ontario: Big Creek | morphological | ROM:Ich:BCF-0367-2 | BCF-0367-2 | EU524930 |
| Notemigonus crysoleucas | Canada: Ontario: Big Creek | morphological | ROM:Ich:BCF-0367-1 | BCF-0367-1 | EU524931 |
| Notemigonus crysoleucas | Canada: Ontario: Fleuve St-Laurent | morphological | ROM:Ich:BCF-0354-1 | BCF-0354-1 | EU524932 |
| Notemigonus crysoleucas | Canada: Quebec: Fleuve St-Laurent, lac St-Louis | morphological | ROM:Ich:BCF-0059-3 | BCF-0059-3 | EU524933 |
| Notemigonus crysoleucas | Canada: Quebec: Fleuve St-Laurent, lac St-Louis | morphological | ROM:Ich:BCF-0059-2 | BCF-0059-2 | EU524934 |
| Notemigonus crysoleucas | Canada: Quebec: Fleuve St-Laurent, lac St-Louis | morphological | ROM:Ich:BCF-0059-1 | BCF-0059-1 | EU524935 |
| Notemigonus crysoleucas | Canada: Quebec: Marais St-Eugene | morphological | ROM:Ich:BCF-0058-2 | BCF-0058-2 | EU524936 |
| Notemigonus crysoleucas | Canada: Quebec: Marais St-Eugene | morphological | ROM:Ich:BCF-005810 | BCF-005810 | EU524937 |
| Notemigonus crysoleucas | Canada: Quebec: Fleuve St-Laurent, lac St-Pierre | tissue | UOG:Bio:BCF-0056-3 | BCF-0056-3 | EU524938 |
| Notemigonus crysoleucas | Canada: Quebec: Fleuve St-Laurent, lac St-Pierre | tissue | UOG:Bio:BCF-0056-2 | BCF-0056-2 | EU524939 |
| Notemigonus crysoleucas | Canada: Quebec: Fleuve St-Laurent, lac St-Pierre | tissue | UOG:Bio:BCF-0056-1 | BCF-0056-1 | EU524940 |
| Notropis anogenus | Canada: Ontario: Saint Clair River, McLeod creek | morphological | ROM:Ich:BCF-0760-8 | BCF-0760-8 | EU524162 |
| Notropis anogenus | Canada: Ontario: Saint Clair River, McLeod creek | morphological | ROM:Ich:BCF-0760-7 | BCF-0760-7 | EU524163 |
| Notropis anogenus | Canada: Ontario: Saint Clair River, McLeod creek | morphological | ROM:Ich:BCF-0760-6 | BCF-0760-6 | EU524164 |
| Notropis anogenus | Canada: Ontario: Saint Clair River, McLeod creek | morphological | ROM:Ich:BCF-0760-5 | BCF-0760-5 | EU524165 |
| Notropis anogenus | Canada: Ontario: Saint Clair River, McLeod creek | morphological | ROM:Ich:BCF-0760-3 | BCF-0760-3 | EU524166 |
| Notropis anogenus | Canada: Ontario: Saint Clair River, McLeod creek | morphological | ROM:Ich:BCF-0760-2 | BCF-0760-2 | EU524167 |
| Notropis anogenus | Canada: Ontario: Saint Clair River, McLeod creek | morphological | ROM:Ich:BCF-0760-1 | BCF-0760-1 | EU524168 |
| Notropis anogenus | Canada: Ontario: Lake Huron, Au sable Channel | morphological | ROM:Ich:BCF-0572-4 | BCF-0572-4 | EU524941 |
| Notropis anogenus | Canada: Ontario: Lake Huron, Au sable Channel | morphological | ROM:Ich:BCF-0572-3 | BCF-0572-3 | EU524942 |
| Notropis anogenus | Canada: Ontario: Lake Huron, Au sable Channel | morphological | ROM:Ich:BCF-0572-2 | BCF-0572-2 | EU524943 |
| Notropis anogenus | Canada: Ontario: Lake St Clair | morphological | ROM:Ich:BCF-0506-4 | BCF-0506-4 | EU524944 |
| Notropis anogenus | Canada: Ontario: Lake St Clair | morphological | ROM:Ich:BCF-0506-2 | BCF-0506-2 | EU524945 |
| Notropis anogenus | Canada: Ontario: Fleuve St-Laurent | morphological | ROM:Ich:BCF-0353-4 | BCF-0353-4 | EU524946 |
| Notropis anogenus | Canada: Ontario: Fleuve St-Laurent | morphological | ROM:Ich:BCF-0353-3 | BCF-0353-3 | EU524947 |
| Notropis anogenus | Canada: Ontario: Fleuve St-Laurent | morphological | ROM:Ich:BCF-0353-2 | BCF-0353-2 | EU524948 |
| Notropis anogenus | Canada: Ontario: Fleuve St-Laurent | morphological | ROM:Ich:BCF-0353-1 | BCF-0353-1 | EU524949 |
| Notropis atherinoides | Canada: Quebec: Archipelles du Lac Saint-Pierre | morphological | ROM:Ich:BCF-0256-1 | BCF-0256-1 | EU524169 |
| Notropis atherinoides | Canada: Quebec: Lac Saint-Pierre, Pointe Yamachiche | morphological | ROM:Ich:BCF-0061-1 | BCF-0061-1 | EU524170 |
| Notropis atherinoides | Canada: Quebec: Lac Joannes | morphological | ROM:Ich:BCF-0257-1 | BCF-0257-1 | EU524171 |
| Notropis atherinoides | Canada: Ontario: Thames River | morphological | ROM:Ich:BCF-0366-4 | BCF-0366-4 | EU524950 |
| Notropis atherinoides | Canada: Ontario: Thames River | morphological | ROM:Ich:BCF-0366-3 | BCF-0366-3 | EU524951 |
| Notropis atherinoides | Canada: Ontario: Thames River | morphological | ROM:Ich:BCF-0366-2 | BCF-0366-2 | EU524952 |
| Notropis atherinoides | Canada: Ontario: Thames River | morphological | ROM:Ich:BCF-0366-1 | BCF-0366-1 | EU524953 |
| Notropis atherinoides | Canada: Manitoba: Lac Winnipeg | tissue | UOG:Bio:BCF-0322-4 | BCF-0322-4 | EU524954 |
| Notropis atherinoides | Canada: Manitoba: Lac Winnipeg | tissue | UOG:Bio:BCF-0322-3 | BCF-0322-3 | EU524955 |
| Notropis atherinoides | Canada: Manitoba: Lac Winnipeg | tissue | UOG:Bio:BCF-0322-2 | BCF-0322-2 | EU524956 |
| Notropis atherinoides | Canada: Manitoba: Lac Winnipeg | tissue | UOG:Bio:BCF-0322-1 | BCF-0322-1 | EU524957 |
| Notropis atherinoides | Canada: Quebec: Fleuve St-Laurent, Lac St-Pierre | morphological | ROM:Ich:BCF-0256-4 | BCF-0256-4 | EU524958 |
| Notropis atherinoides | Canada: Quebec: Fleuve St-Laurent, Lac St-Pierre | morphological | ROM:Ich:BCF-0256-3 | BCF-0256-3 | EU524959 |
| Notropis atherinoides | Canada: Quebec: Fleuve St-Laurent, lac St-Louis | morphological | ROM:Ich:BCF-0062-3 | BCF-0062-3 | EU524960 |
| Notropis atherinoides | Canada: Quebec: Fleuve St-Laurent, lac St-Louis | morphological | ROM:Ich:BCF-0062-2 | BCF-0062-2 | EU524961 |
| Notropis atherinoides | Canada: Quebec: Fleuve St-Laurent, lac St-Louis | morphological | ROM:Ich:BCF-0062-1 | BCF-0062-1 | EU524962 |
| Notropis bifrenatus | Canada: Quebec: Lac Saint-Paul | morphological | ROM:Ich:BCF-0201-1 | BCF-0201-1 | EU524172 |
| Notropis bifrenatus | Canada: Quebec: Marais Saint-Eugene | morphological | ROM:Ich:BCF-0255-1 | BCF-0255-1 | EU524173 |
| Notropis bifrenatus | Canada: Quebec: Marais Saint-Eugene | morphological | ROM:Ich:BCF-0255-2 | BCF-0255-2 | EU524174 |
| Notropis bifrenatus | Canada: Quebec: Marais St-Eugene | morphological | ROM:Ich:BCF-0255-6 | BCF-0255-6 | EU524963 |
| Notropis bifrenatus | Canada: Quebec: Marais St-Eugene | morphological | ROM:Ich:BCF-0255-5 | BCF-0255-5 | EU524964 |
| Notropis bifrenatus | Canada: Quebec: Marais St-Eugene | morphological | ROM:Ich:BCF-0255-4 | BCF-0255-4 | EU524965 |
| Notropis bifrenatus | Canada: Quebec: Marais St-Eugene | morphological | ROM:Ich:BCF-0255-3 | BCF-0255-3 | EU524966 |
| Notropis bifrenatus | Canada: Quebec: Fleuve St-Laurent, lac St-Paul | morphological | ROM:Ich:BCF-0201-4 | BCF-0201-4 | EU524967 |
| Notropis bifrenatus | Canada: Quebec: Fleuve St-Laurent, lac St-Paul | morphological | ROM:Ich:BCF-0201-3 | BCF-0201-3 | EU524968 |
| Notropis bifrenatus | Canada: Quebec: Fleuve St-Laurent, lac St-Paul | morphological | ROM:Ich:BCF-0201-2 | BCF-0201-2 | EU524969 |
| Notropis buchanani | Canada: Ontario: Sydenham River | morphological | ROM:Ich:BCF-0409-6 | BCF-0409-6 | EU524970 |
| Notropis buchanani | Canada: Ontario: Sydenham River | morphological | ROM:Ich:BCF-0409-5 | BCF-0409-5 | EU524971 |
| Notropis buchanani | Canada: Ontario: Sydenham River | morphological | ROM:Ich:BCF-0409-4 | BCF-0409-4 | EU524972 |
| Notropis buchanani | Canada: Ontario: Sydenham River | morphological | ROM:Ich:BCF-0409-3 | BCF-0409-3 | EU524973 |
| Notropis buchanani | Canada: Ontario: Sydenham River | morphological | ROM:Ich:BCF-0409-2 | BCF-0409-2 | EU524974 |
| Notropis buchanani | Canada: Ontario: Sydenham River | morphological | ROM:Ich:BCF-0409-1 | BCF-0409-1 | EU524975 |
| Notropis buchanani | Canada: Ontario: Thames River | morphological | ROM:Ich:BCF-0385-6 | BCF-0385-6 | EU524976 |
| Notropis buchanani | Canada: Ontario: Thames River | morphological | ROM:Ich:BCF-0385-5 | BCF-0385-5 | EU524977 |
| Notropis buchanani | Canada: Ontario: Thames River | morphological | ROM:Ich:BCF-0385-4 | BCF-0385-4 | EU524978 |
| Notropis buchanani | Canada: Ontario: Thames River | morphological | ROM:Ich:BCF-0385-3 | BCF-0385-3 | EU524979 |
| Notropis buchanani | Canada: Ontario: Thames River | morphological | ROM:Ich:BCF-0385-2 | BCF-0385-2 | EU524980 |
| Notropis heterodon | Canada: Quebec: Lac Saint-Louis (Fleuve Saint-Laurent) | morphological | ROM:Ich:BCF-0066-1 | BCF-0066-1 | EU524175 |
| Notropis heterodon | Canada: Ontario: Fleuve St-Laurent | morphological | ROM:Ich:BCF-0352-2 | BCF-0352-2 | EU524981 |
| Notropis heterodon | Canada: Ontario: Fleuve St-Laurent | morphological | ROM:Ich:BCF-0352-1 | BCF-0352-1 | EU524982 |
| Notropis heterodon | Canada: Quebec: Fleuve St-Laurent, lac St-Louis | morphological | ROM:Ich:BCF-0066-3 | BCF-0066-3 | EU524983 |
| Notropis heterodon | Canada: Quebec: Fleuve St-Laurent, lac St-Louis | morphological | ROM:Ich:BCF-0066-2 | BCF-0066-2 | EU524984 |
| Notropis heterodon | Canada: Ontario: Fairchild Creek Tributary | morphological | ROM:Ich:BCF-0507-4 | BCF-0507-4 | EU524985 |
| Notropis heterodon | Canada: Ontario: Lake St Clair | morphological | ROM:Ich:BCF-0507-3 | BCF-0507-3 | EU524986 |
| Notropis heterodon | Canada: Ontario: Lake St Clair | morphological | ROM:Ich:BCF-0507-2 | BCF-0507-2 | EU524987 |
| Notropis heterodon | Canada: Ontario: Lake St Clair | morphological | ROM:Ich:BCF-0507-1 | BCF-0507-1 | EU524988 |
| Notropis heterodon | Canada: Ontario: Fleuve St-Laurent | morphological | ROM:Ich:BCF-0352-5 | BCF-0352-5 | EU524989 |
| Notropis heterodon | Canada: Ontario: Fleuve St-Laurent | morphological | ROM:Ich:BCF-0352-4 | BCF-0352-4 | EU524990 |
| Notropis heterodon | Canada: Ontario: Fleuve St-Laurent | morphological | ROM:Ich:BCF-0352-3 | BCF-0352-3 | EU524991 |
| Notropis heterolepis | Canada: New Brunswick: Yoho Lake | morphological | ROM:Ich:BCF-0587-3 | BCF-0587-3 | EU524992 |
| Notropis heterolepis | Canada: New Brunswick: Yoho Lake | morphological | ROM:Ich:BCF-0587-2 | BCF-0587-2 | EU524993 |
| Notropis heterolepis | Canada: New Brunswick: Yoho Lake | morphological | ROM:Ich:BCF-0587-1 | BCF-0587-1 | EU524994 |
| Notropis heterolepis | Canada: Ontario: Westward Lake | morphological | ROM:Ich:BCF-0535-2 | BCF-0535-2 | EU524995 |
| Notropis heterolepis | Canada: Ontario: Westward Lake | morphological | ROM:Ich:BCF-0535-1 | BCF-0535-1 | EU524996 |
| Notropis heterolepis | Canada: Ontario: Wanapitei River | morphological | ROM:Ich:BCF-0438-3 | BCF-0438-3 | EU524997 |
| Notropis heterolepis | Canada: Ontario: Wanapitei River | morphological | ROM:Ich:BCF-0438-2 | BCF-0438-2 | EU524998 |
| Notropis heterolepis | Canada: Ontario: Wanapitei River | morphological | ROM:Ich:BCF-0438-1 | BCF-0438-1 | EU524999 |
| Notropis hudsonius | Canada: Quebec: Lac Saint-Pierre, Pointe Yamachiche | morphological | ROM:Ich:BCF-0050-1 | BCF-0050-1 | EU524176 |
| Notropis hudsonius | Canada: Ontario: St Clair River | morphological | ROM:Ich:BCF-0526-2 | BCF-0526-2 | EU525000 |
| Notropis hudsonius | Canada: Ontario: St Clair River | morphological | ROM:Ich:BCF-0526-1 | BCF-0526-1 | EU525001 |
| Notropis hudsonius | Canada: Ontario: Lake Erie | morphological | ROM:Ich:BCF-0362-3 | BCF-0362-3 | EU525002 |
| Notropis hudsonius | Canada: Ontario: Lake Erie | morphological | ROM:Ich:BCF-0362-2 | BCF-0362-2 | EU525003 |
| Notropis hudsonius | Canada: Ontario: Lake Erie | morphological | ROM:Ich:BCF-0362-1 | BCF-0362-1 | EU525004 |
| Notropis hudsonius | Canada: Quebec: Fleuve St-Laurent, lac St-Pierre | morphological | ROM:Ich:BCF-0050-3 | BCF-0050-3 | EU525005 |
| Notropis hudsonius | Canada: Quebec: Fleuve St-Laurent, lac St-Pierre | morphological | ROM:Ich:BCF-0050-2 | BCF-0050-2 | EU525006 |
| Notropis percobromus | Canada: Manitoba: Winnipeg River | tissue | UOG:Bio:BCF-0840-1 | BCF-0840-1 | EU524177 |
| Notropis photogenis | Canada: Ontario: Bronte Creek | morphological | ROM:Ich:BCF-0521-9 | BCF-0521-9 | EU525007 |
| Notropis photogenis | Canada: Ontario: Bronte Creek | morphological | ROM:Ich:BCF-0521-7 | BCF-0521-7 | EU525008 |
| Notropis photogenis | Canada: Ontario: Bronte Creek | morphological | ROM:Ich:BCF-0521-6 | BCF-0521-6 | EU525009 |
| Notropis photogenis | Canada: Ontario: Bronte Creek | morphological | ROM:Ich:BCF-0521-5 | BCF-0521-5 | EU525010 |
| Notropis photogenis | Canada: Ontario: Bronte Creek | morphological | ROM:Ich:BCF-0521-4 | BCF-0521-4 | EU525011 |
| Notropis photogenis | Canada: Ontario: Bronte Creek | morphological | ROM:Ich:BCF-0521-3 | BCF-0521-3 | EU525012 |
| Notropis photogenis | Canada: Ontario: Bronte Creek | morphological | ROM:Ich:BCF-0521-2 | BCF-0521-2 | EU525013 |
| Notropis photogenis | Canada: Ontario: Bronte Creek | morphological | ROM:Ich:BCF-0521-1 | BCF-0521-1 | EU525014 |
| Notropis photogenis | Canada: Ontario: Grand River | morphological | ROM:Ich:BCF-0386-1 | BCF-0386-1 | EU525015 |
| Notropis rubellus | Canada: Quebec: Batiscan River | morphological | ROM:Ich:BCF-0069-1 | BCF-0069-1 | EU524178 |
| Notropis rubellus | Canada: Ontario: Grand River | morphological | ROM:Ich:BCF-0387-4 | BCF-0387-4 | EU525016 |
| Notropis rubellus | Canada: Ontario: Grand River | morphological | ROM:Ich:BCF-0387-3 | BCF-0387-3 | EU525017 |
| Notropis rubellus | Canada: Ontario: Grand River | morphological | ROM:Ich:BCF-0387-2 | BCF-0387-2 | EU525018 |
| Notropis rubellus | Canada: Ontario: Grand River | morphological | ROM:Ich:BCF-0387-1 | BCF-0387-1 | EU525019 |
| Notropis rubellus | Canada: Quebec: Batiscan River | morphological | ROM:Ich:BCF-0069-2 | BCF-0069-2 | EU525020 |
| Notropis rubellus | Canada: Quebec: Batiscan River | morphological | ROM:Ich:BCF-0069-13 | BCF-0069-13 | EU525021 |
| Notropis rubellus | Canada: Quebec: Batiscan River | morphological | ROM:Ich:BCF-0069-10 | BCF-0069-10 | EU525022 |
| Notropis stramineus | Canada: Quebec: Fleuve St-Laurent, lac St-Louis | morphological | ROM:Ich:BCF-0072-1 | BCF-0072-1 | EU524179 |
| Notropis stramineus | Canada: Quebec: Lac Saint-Pierre , pointe Yamachiche | morphological | ROM:Ich:BCF-0252-1 | BCF-0252-1 | EU524180 |
| Notropis stramineus | Canada: Quebec: Fleuve St-Laurent, lac St-Louis | morphological | ROM:Ich:BCF-0071-1 | BCF-0071-1 | EU524181 |
| Notropis stramineus | Canada: Quebec: Fleuve St-Laurent, lac St-Louis | morphological | ROM:Ich:BCF-0071-2 | BCF-0071-2 | EU525023 |
| Notropis stramineus | Canada: Ontario: Lake Erie | morphological | ROM:Ich:BCF-0361-7 | BCF-0361-7 | EU525024 |
| Notropis stramineus | Canada: Ontario: Lake Erie | morphological | ROM:Ich:BCF-0361-6 | BCF-0361-6 | EU525025 |
| Notropis stramineus | Canada: Ontario: Lake Erie | morphological | ROM:Ich:BCF-0361-5 | BCF-0361-5 | EU525026 |
| Notropis stramineus | Canada: Ontario: Lake Erie | morphological | ROM:Ich:BCF-0361-4 | BCF-0361-4 | EU525027 |
| Notropis stramineus | Canada: Ontario: Lake Erie | morphological | ROM:Ich:BCF-0361-3 | BCF-0361-3 | EU525028 |
| Notropis stramineus | Canada: Ontario: Lake Erie | morphological | ROM:Ich:BCF-0361-2 | BCF-0361-2 | EU525029 |
| Notropis stramineus | Canada: Ontario: Lake Erie | morphological | ROM:Ich:BCF-0361-10 | BCF-0361-10 | EU525030 |
| Notropis stramineus | Canada: Ontario: Lake Erie | morphological | ROM:Ich:BCF-0361-1 | BCF-0361-1 | EU525031 |
| Notropis texanus | Canada: Manitoba: Winnipeg River, Seven sisters | tissue | UOG:Bio:BCF-0857-1 | BCF-0857-1 | EU524182 |
| Notropis volucellus | Canada: Ontario: Sydenham River | morphological | ROM:Ich:BCF-0417-4 | BCF-0417-4 | EU524183 |
| Notropis volucellus | Canada: Ontario: Grand River | morphological | ROM:Ich:BCF-0388-4 | BCF-0388-4 | EU524184 |
| Notropis volucellus | Canada: Ontario: Sydenham River | morphological | ROM:Ich:BCF-0417-3 | BCF-0417-3 | EU525032 |
| Notropis volucellus | Canada: Ontario: Sydenham River | morphological | ROM:Ich:BCF-0417-2 | BCF-0417-2 | EU525033 |
| Notropis volucellus | Canada: Ontario: Sydenham River | morphological | ROM:Ich:BCF-0417-1 | BCF-0417-1 | EU525034 |
| Notropis volucellus | Canada: Ontario: Thames River | morphological | ROM:Ich:BCF-0388-6 | BCF-0388-6 | EU525035 |
| Notropis volucellus | Canada: Ontario: Grand River | morphological | ROM:Ich:BCF-0388-3 | BCF-0388-3 | EU525036 |
| Notropis volucellus | Canada: Ontario: Grand River | morphological | ROM:Ich:BCF-0388-2 | BCF-0388-2 | EU525037 |
| Notropis volucellus | Canada: Ontario: Grand River | morphological | ROM:Ich:BCF-0388-1 | BCF-0388-1 | EU525038 |
| Phoxinus eos | Canada: Ontario: Wetland 1 | morphological | ROM:Ich:BCF-0465-1 | BCF-0465-1 | EU525058 |
| Phoxinus eos | Canada: Ontario: Wetland 6 | morphological | ROM:Ich:BCF-0459-1 | BCF-0459-1 | EU525059 |
| Phoxinus eos | Canada: Quebec: Reserve Rouge-Matawin, lac Dalpec | morphological | ROM:Ich:BCF-0073-13 | BCF-0073-13 | EU525060 |
| Phoxinus eos | Canada: Quebec: Reserve Rouge-Matawin, lac Dalpec | morphological | ROM:Ich:BCF-0073-12 | BCF-0073-12 | EU525061 |
| Phoxinus eos | Canada: Quebec: Reserve Rouge-Matawin, lac Dalpec | morphological | ROM:Ich:BCF-0073-11 | BCF-0073-11 | EU525062 |
| Phoxinus eos | Canada: Quebec: Reserve Rouge-Matawin, lac Dalpec | morphological | ROM:Ich:BCF-0073-10 | BCF-0073-10 | EU525063 |
| Phoxinus neogaeus | Canada: Quebec: Reserve Rouge-Matawin, lac Dalpec | morphological | ROM:Ich:BCF-0074-1 | BCF-0074-1 | EU524274 |
| Phoxinus neogaeus | Canada: Quebec: Reserve Rouge-Matawin, lac Dalpec | morphological | ROM:Ich:BCF-0073-1 | BCF-0073-1 | EU524275 |
| Phoxinus neogaeus | Canada: Ontario: Wetland 1 | morphological | ROM:Ich:BCF-0562-1 | BCF-0562-1 | EU525064 |
| Phoxinus neogaeus | Canada: Ontario: Wetland A | morphological | ROM:Ich:BCF-0461-3 | BCF-0461-3 | EU525065 |
| Phoxinus neogaeus | Canada: Ontario: Wetland A | morphological | ROM:Ich:BCF-0461-2 | BCF-0461-2 | EU525066 |
| Phoxinus neogaeus | Canada: Ontario: Wetland A | morphological | ROM:Ich:BCF-0461-1 | BCF-0461-1 | EU525067 |
| Phoxinus neogaeus | Canada: Quebec: Aylmer, Compte de Pontiac | morphological | ROM:Ich:BCF-0277-3 | BCF-0277-3 | EU525068 |
| Phoxinus neogaeus | Canada: Quebec: Aylmer, Compte de Pontiac | morphological | ROM:Ich:BCF-0277-2 | BCF-0277-2 | EU525069 |
| Phoxinus neogaeus | Canada: Quebec: Aylmer, Compte de Pontiac | morphological | ROM:Ich:BCF-0277-1 | BCF-0277-1 | EU525070 |
| Phoxinus neogaeus | Canada: Quebec: Reserve Rouge-Matawin, lac Dalpec | morphological | ROM:Ich:BCF-0074-3 | BCF-0074-3 | EU525071 |
| Phoxinus neogaeus | : : | morphological | ROM:Ich:BCF-0074-2 | BCF-0074-2 | EU525072 |
| Phoxinus neogaeus | : : | morphological | ROM:Ich:BCF-0073-2 | BCF-0073-2 | EU525073 |
| Pimephales notatus | Canada: Quebec: Fleuve St-Laurent, lac St-Louis | morphological | ROM:Ich:BCF-0075-1 | BCF-0075-1 | EU524276 |
| Pimephales notatus | Canada: Ontario: Grand River | morphological | ROM:Ich:BCF-0401-3 | BCF-0401-3 | EU525074 |
| Pimephales notatus | Canada: Ontario: Grand River | morphological | ROM:Ich:BCF-0401-2 | BCF-0401-2 | EU525075 |
| Pimephales notatus | Canada: Ontario: Grand River | morphological | ROM:Ich:BCF-0401-1 | BCF-0401-1 | EU525076 |
| Pimephales notatus | Canada: Ontario: Lk. Huron-McGregor Bay | morphological | ROM:Ich:BCF-0341-3 | BCF-0341-3 | EU525077 |
| Pimephales notatus | Canada: Ontario: Lac Opinicon | tissue | UOG:Bio:BCF-0216-3 | BCF-0216-3 | EU525078 |
| Pimephales notatus | Canada: Ontario: Lac Opinicon | tissue | UOG:Bio:BCF-0216-2 | BCF-0216-2 | EU525079 |
| Pimephales notatus | Canada: Quebec: Fleuve St-Laurent, Lac St-Pierre | morphological | ROM:Ich:BCF-0202-3 | BCF-0202-3 | EU525080 |
| Pimephales notatus | Canada: Quebec: Fleuve St-Laurent, Lac St-Pierre | morphological | ROM:Ich:BCF-0202-2 | BCF-0202-2 | EU525081 |
| Pimephales notatus | Canada: Quebec: Fleuve St-Laurent, Lac St-Pierre | morphological | ROM:Ich:BCF-0202-1 | BCF-0202-1 | EU525082 |
| Pimephales notatus | Canada: Quebec: Fleuve St-Laurent, lac St-Louis | morphological | ROM:Ich:BCF-0075-3 | BCF-0075-3 | EU525083 |
| Pimephales notatus | Canada: Quebec: Fleuve St-Laurent, lac St-Louis | morphological | ROM:Ich:BCF-0075-2 | BCF-0075-2 | EU525084 |
| Pimephales promelas | Canada: Quebec: Lac Wapizagonke | morphological | ROM:Ich:BCF-0078-1 | BCF-0078-1 | EU524277 |
| Pimephales promelas | Canada: Ontario: Wetland 1 | morphological | ROM:Ich:BCF-0472-3 | BCF-0472-3 | EU525085 |
| Pimephales promelas | Canada: Ontario: Wetland 1 | morphological | ROM:Ich:BCF-0472-1 | BCF-0472-1 | EU525086 |
| Pimephales promelas | Canada: Ontario: Wetland E | morphological | ROM:Ich:BCF-0462-3 | BCF-0462-3 | EU525087 |
| Pimephales promelas | Canada: Ontario: Wetland E | morphological | ROM:Ich:BCF-0462-2 | BCF-0462-2 | EU525088 |
| Pimephales promelas | Canada: Ontario: Wetland E | morphological | ROM:Ich:BCF-0462-1 | BCF-0462-1 | EU525089 |
| Pimephales promelas | Canada: Quebec: Fleuve St-Laurent, riviere St-Jean | morphological | ROM:Ich:BCF-0295-3 | BCF-0295-3 | EU525090 |
| Pimephales promelas | Canada: Quebec: Fleuve St-Laurent, riviere St-Jean | morphological | ROM:Ich:BCF-0295-2 | BCF-0295-2 | EU525091 |
| Pimephales promelas | Canada: Quebec: Etang de Graviere | morphological | ROM:Ich:BCF-0265-3 | BCF-0265-3 | EU525092 |
| Pimephales promelas | Canada: Quebec: Etang de Graviere | morphological | ROM:Ich:BCF-0265-1 | BCF-0265-1 | EU525093 |
| Pimephales promelas | Canada: Quebec: lac Wapizagonke | morphological | ROM:Ich:BCF-0078-3 | BCF-0078-3 | EU525094 |
| Pimephales promelas | Canada: Quebec: lac Wapizagonke | morphological | ROM:Ich:BCF-0078-2 | BCF-0078-2 | EU525095 |
| Ptychocheilus oregonensis | Canada: British Columbia: Summit lake | morphological | ROM:Ich:BCF-0704-4 | BCF-0704-4 | EU524311 |
| Ptychocheilus oregonensis | Canada: British Columbia: Summit lake | morphological | ROM:Ich:BCF-0704-3 | BCF-0704-3 | EU524312 |
| Ptychocheilus oregonensis | Canada: British Columbia: Summit lake | morphological | ROM:Ich:BCF-0704-2 | BCF-0704-2 | EU524313 |
| Ptychocheilus oregonensis | Canada: British Columbia: Summit lake | morphological | ROM:Ich:BCF-0704-1 | BCF-0704-1 | EU524314 |
| Ptychocheilus oregonensis | Canada: British Columbia: Crooked river | morphological | ROM:Ich:BCF-0703-4 | BCF-0703-4 | EU524315 |
| Ptychocheilus oregonensis | Canada: British Columbia: Crooked river | morphological | ROM:Ich:BCF-0703-3 | BCF-0703-3 | EU524316 |
| Ptychocheilus oregonensis | Canada: British Columbia: Crooked river | morphological | ROM:Ich:BCF-0703-2 | BCF-0703-2 | EU524317 |
| Ptychocheilus oregonensis | Canada: British Columbia: Crooked river | morphological | ROM:Ich:BCF-0703-1 | BCF-0703-1 | EU524318 |
| Rhinichthys atratulus | Canada: Quebec: Riviere Becancour | tissue | UOG:Bio:BCF-0079-1 | BCF-0079-1 | EU524322 |
| Rhinichthys atratulus | Canada: New Brunswick: Mc Quarrie Brook | morphological | ROM:Ich:BCF-0585-4 | BCF-0585-4 | EU525115 |
| Rhinichthys atratulus | Canada: New Brunswick: Mc Quarrie Brook | morphological | ROM:Ich:BCF-0585-3 | BCF-0585-3 | EU525116 |
| Rhinichthys atratulus | Canada: New Brunswick: Mc Quarrie Brook | morphological | ROM:Ich:BCF-0585-2 | BCF-0585-2 | EU525117 |
| Rhinichthys atratulus | Canada: New Brunswick: Mc Quarrie Brook | morphological | ROM:Ich:BCF-0585-1 | BCF-0585-1 | EU525118 |
| Rhinichthys atratulus | Canada: Quebec: Fleuve St-Laurent, riviere Becancour | tissue | UOG:Bio:BCF-0079-4 | BCF-0079-4 | EU525119 |
| Rhinichthys atratulus | Canada: Quebec: Fleuve St-Laurent, riviere Becancour | tissue | UOG:Bio:BCF-0079-2 | BCF-0079-2 | EU525120 |
| Rhinichthys cataractae | Canada: Ontario: Maitland River | morphological | ROM:Ich:BCF-0773-2 | BCF-0773-2 | EU524323 |
| Rhinichthys cataractae | Canada: Ontario: Maitland River | morphological | ROM:Ich:BCF-0773-1 | BCF-0773-1 | EU524324 |
| Rhinichthys cataractae | Canada: Ontario: Thames river | morphological | ROM:Ich:BCF-0740-2 | BCF-0740-2 | EU524325 |
| Rhinichthys cataractae | Canada: Ontario: Thames river | morphological | ROM:Ich:BCF-0740-1 | BCF-0740-1 | EU524326 |
| Rhinichthys cataractae | Canada: Quebec: Riviere Sainte-Marguerite | morphological | ROM:Ich:BCF-0081-1 | BCF-0081-1 | EU524327 |
| Rhinichthys cataractae | Canada: Quebec: Fleuve St-Laurent, riviere Ste-Mars | morphological | ROM:Ich:BCF-0263-3 | BCF-0263-3 | EU525121 |
| Rhinichthys cataractae | Canada: Quebec: Fleuve St-Laurent, riviere Ste-Mars | morphological | ROM:Ich:BCF-0263-2 | BCF-0263-2 | EU525122 |
| Rhinichthys cataractae | Canada: Quebec: Fleuve St-Laurent, riviere Ste-Mars | morphological | ROM:Ich:BCF-0263-1 | BCF-0263-1 | EU525123 |
| Rhinichthys cataractae | Canada: Quebec: Fleuve St-Laurent, riviere Cap-Rouge | morphological | ROM:Ich:BCF-0082-4 | BCF-0082-4 | EU525124 |
| Rhinichthys cataractae | Canada: Quebec: Fleuve St-Laurent, riviere Cap-Rouge | morphological | ROM:Ich:BCF-0082-3 | BCF-0082-3 | EU525125 |
| Rhinichthys cataractae | Canada: Quebec: Fleuve St-Laurent, riviere Cap-Rouge | morphological | ROM:Ich:BCF-0082-1 | BCF-0082-1 | EU525126 |
| Rhinichthys cataractae | Canada: Quebec: Fleuve St-Laurent, riviere Ste-Marguerite | morphological | ROM:Ich:BCF-0081-12 | BCF-0081-12 | EU525127 |
| Rhinichthys cataractae | Canada: Quebec: Fleuve St-Laurent, riviere Ste-Marguerite | morphological | ROM:Ich:BCF-0081-11 | BCF-0081-11 | EU525128 |
| Rhinichthys cataractae | Canada: Quebec: Fleuve St-Laurent, riviere Ste-Marguerite | morphological | ROM:Ich:BCF-0081-10 | BCF-0081-10 | EU525129 |
| Rhinichthys cataractae | Canada: Quebec: Fleuve St-Laurent, riviere Becancour | tissue | UOG:Bio:BCF-0079-3 | BCF-0079-3 | EU525130 |
| Rhinichthys falcatus | Canada: British Columbia: Fraser river | tissue | UOG:Bio:BCF-0661-5 | BCF-0661-5 | EU524328 |
| Rhinichthys falcatus | Canada: British Columbia: Fraser river | tissue | UOG:Bio:BCF-0661-4 | BCF-0661-4 | EU524329 |
| Rhinichthys falcatus | Canada: British Columbia: Fraser river | tissue | UOG:Bio:BCF-0661-3 | BCF-0661-3 | EU524330 |
| Rhinichthys falcatus | Canada: British Columbia: Fraser river | tissue | UOG:Bio:BCF-0661-2 | BCF-0661-2 | EU524331 |
| Rhinichthys falcatus | Canada: British Columbia: Fraser river | tissue | UOG:Bio:BCF-0661-1 | BCF-0661-1 | EU524332 |
| Rhinichthys obtusus | Canada: Ontario: Sydenham River | morphological | ROM:Ich:BCF-0770-1 | BCF-0770-1 | EU524333 |
| Rhinichthys obtusus | Canada: Ontario: Credit River | morphological | ROM:Ich:BCF-0619-3 | BCF-0619-3 | EU524334 |
| Rhinichthys obtusus | Canada: Ontario: Marden Creek | morphological | ROM:Ich:BCF-0619-2 | BCF-0619-2 | EU524335 |
| Rhinichthys obtusus | Canada: Ontario: Humber River | morphological | ROM:Ich:BCF-0619-1 | BCF-0619-1 | EU524336 |
| Rhinichthys obtusus | Canada: Ontario: Humber River | morphological | ROM:Ich:BCF-0483-1 | BCF-0483-1 | EU525131 |
| Rhinichthys obtusus | Canada: Ontario: Credit River | morphological | ROM:Ich:BCF-0439-3 | BCF-0439-3 | EU525132 |
| Rhinichthys obtusus | Canada: Ontario: Marden Creek | morphological | ROM:Ich:BCF-0439-2 | BCF-0439-2 | EU525133 |
| Rhinichthys obtusus | Canada: Ontario: Credit River | morphological | ROM:Ich:BCF-0439-1 | BCF-0439-1 | EU525134 |
| Rhinichthys osculus | Canada: British Columbia: Kettle river | tissue | UOG:Bio:BCF-0666-5 | BCF-0666-5 | EU524337 |
| Rhinichthys osculus | Canada: British Columbia: Kettle river | tissue | UOG:Bio:BCF-0666-4 | BCF-0666-4 | EU524338 |
| Rhinichthys osculus | Canada: British Columbia: Kettle river | tissue | UOG:Bio:BCF-0666-3 | BCF-0666-3 | EU524339 |
| Rhinichthys osculus | Canada: British Columbia: Kettle river | tissue | UOG:Bio:BCF-0666-2 | BCF-0666-2 | EU524340 |
| Rhinichthys osculus | Canada: British Columbia: Kettle river | tissue | UOG:Bio:BCF-0666-1 | BCF-0666-1 | EU524341 |
| Rhinichthys umatilla | Canada: British Columbia: Similkanmeen river | tissue | UOG:Bio:BCF-0662-5 | BCF-0662-5 | EU524342 |
| Rhinichthys umatilla | Canada: British Columbia: Similkanmeen river | tissue | UOG:Bio:BCF-0662-4 | BCF-0662-4 | EU524343 |
| Rhinichthys umatilla | Canada: British Columbia: Similkanmeen river | tissue | UOG:Bio:BCF-0662-3 | BCF-0662-3 | EU524344 |
| Rhinichthys umatilla | Canada: British Columbia: Similkanmeen river | tissue | UOG:Bio:BCF-0662-2 | BCF-0662-2 | EU524345 |
| Rhinichthys umatilla | Canada: British Columbia: Similkanmeen river | tissue | UOG:Bio:BCF-0662-1 | BCF-0662-1 | EU524346 |
| Richardsonius balteatus | Canada: British Columbia: Fraser river | tissue | UOG:Bio:BCF-0685-5 | BCF-0685-5 | EU524347 |
| Richardsonius balteatus | Canada: British Columbia: Fraser river | tissue | UOG:Bio:BCF-0685-3 | BCF-0685-3 | EU524348 |
| Scardinius erythrophthalmus | Canada: Ontario: Welland river, city of welland | tissue | UOG:Bio:BCF-0726-1 | BCF-0726-1 | EU524381 |
| Scardinius erythrophthalmus | Canada: Quebec: Lac St-Pierre | tissue | UOG:Bio:BCF-0494-1 | BCF-0494-1 | EU525135 |
| Semotilus atromaculatus | Canada: Ontario: Wetland 8 | morphological | ROM:Ich:BCF-0474-3 | BCF-0474-3 | EU525136 |
| Semotilus atromaculatus | Canada: Ontario: Wetland 8 | morphological | ROM:Ich:BCF-0474-2 | BCF-0474-2 | EU525137 |
| Semotilus atromaculatus | Canada: Ontario: Wetland 8 | morphological | ROM:Ich:BCF-0474-1 | BCF-0474-1 | EU525138 |
| Semotilus atromaculatus | Canada: Ontario: Sydenham River | morphological | ROM:Ich:BCF-0412-1 | BCF-0412-1 | EU525139 |
| Semotilus atromaculatus | Canada: Quebec: Fleuve St-Laurent, riviere St-Jean | morphological | ROM:Ich:BCF-0290-3 | BCF-0290-3 | EU525140 |
| Semotilus atromaculatus | Canada: Quebec: Fleuve St-Laurent, riviere St-Jean | morphological | ROM:Ich:BCF-0290-2 | BCF-0290-2 | EU525141 |
| Semotilus atromaculatus | Canada: Quebec: Fleuve St-Laurent, riviere St-Charles | morphological | ROM:Ich:BCF-0229-3 | BCF-0229-3 | EU525142 |
| Semotilus atromaculatus | Canada: Quebec: Fleuve St-Laurent, riviere St-Charles | morphological | ROM:Ich:BCF-0229-2 | BCF-0229-2 | EU525143 |
| Semotilus atromaculatus | Canada: Quebec: Fleuve St-Laurent, riviere St-Charles | morphological | ROM:Ich:BCF-0229-1 | BCF-0229-1 | EU525144 |
| Semotilus corporalis | Canada: Quebec: Riviere Sainte-Marguerite | morphological | ROM:Ich:BCF-0254-1 | BCF-0254-1 | EU524382 |
| Semotilus corporalis | Canada: Quebec: Riviere Becancour | morphological | ROM:Ich:BCF-0086-1 | BCF-0086-1 | EU524383 |
| Semotilus corporalis | Canada: Ontario: York River | morphological | ROM:Ich:BCF-0528-3 | BCF-0528-3 | EU525145 |
| Semotilus corporalis | Canada: Ontario: York River | morphological | ROM:Ich:BCF-0528-2 | BCF-0528-2 | EU525146 |
| Semotilus corporalis | Canada: Ontario: York River | morphological | ROM:Ich:BCF-0528-1 | BCF-0528-1 | EU525147 |
| Semotilus corporalis | Canada: Quebec: Fleuve St-Laurent, riviere Cap-Rouge | morphological | ROM:Ich:BCF-0088-2 | BCF-0088-2 | EU525148 |
| Semotilus corporalis | Canada: Quebec: Fleuve St-Laurent, riviere Cap-Rouge | morphological | ROM:Ich:BCF-0088-1 | BCF-0088-1 | EU525149 |
| Semotilus corporalis | Canada: Quebec: Fleuve St-Laurent, riviere Becancour | morphological | ROM:Ich:BCF-0086-4 | BCF-0086-4 | EU525150 |
| Semotilus corporalis | Canada: Quebec: Fleuve St-Laurent, riviere Becancour | morphological | ROM:Ich:BCF-0086-3 | BCF-0086-3 | EU525151 |
| Semotilus corporalis | Canada: Quebec: Fleuve St-Laurent, riviere Becancour | morphological | ROM:Ich:BCF-0086-2 | BCF-0086-2 | EU525152 |
| Tinca tinca | Canada: Quebec: Riviere Richelieu | tissue | UOG:Bio:BCF-0238-1 | BCF-0238-1 | EU524390 |
| Tinca tinca | Canada: Quebec: Fleuve St-Laurent, riviere Richelieu | tissue | UOG:Bio:BCF-0238-19 | BCF-0238-19 | EU525153 |
| Tinca tinca | Canada: Quebec: Fleuve St-Laurent, riviere Richelieu | tissue | UOG:Bio:BCF-0238-18 | BCF-0238-18 | EU525154 |
| Tinca tinca | Canada: Quebec: Fleuve St-Laurent, riviere Richelieu | tissue | UOG:Bio:BCF-0238-17 | BCF-0238-17 | EU525155 |
| Tinca tinca | Canada: Quebec: Fleuve St-Laurent, riviere Richelieu | tissue | UOG:Bio:BCF-0238-16 | BCF-0238-16 | EU525156 |
| Tinca tinca | Canada: Quebec: Fleuve St-Laurent, riviere Richelieu | tissue | UOG:Bio:BCF-0238-15 | BCF-0238-15 | EU525157 |
| Tinca tinca | Canada: Quebec: Fleuve St-Laurent, riviere Richelieu | tissue | UOG:Bio:BCF-0238-14 | BCF-0238-14 | EU525158 |
| Tinca tinca | Canada: Quebec: Fleuve St-Laurent, riviere Richelieu | tissue | UOG:Bio:BCF-0238-13 | BCF-0238-13 | EU525159 |
| Tinca tinca | Canada: Quebec: Fleuve St-Laurent, riviere Richelieu | tissue | UOG:Bio:BCF-0238-11 | BCF-0238-11 | EU525160 |
| Tinca tinca | Canada: Quebec: Fleuve St-Laurent, riviere Richelieu | tissue | UOG:Bio:BCF-0238-10 | BCF-0238-10 | EU525161 |
| Tinca tinca | Canada: Quebec: Fleuve St-Laurent, riviere Richelieu | tissue | UOG:Bio:BCF-0238-12 | BCF-0238-12 | EU525162 |
| Esox americanus | Canada: Quebec: Marais St-Eugene | morphological | ROM:Ich:BCF-0045-1 | BCF-0045-1 | EU524009 |
| Esox americanus | Canada: Ontario: Twenty Mile Creek | morphological | ROM:Ich:BCF-0452-1 | BCF-0452-1 | EU524568 |
| Esox americanus | Canada: Quebec: Richelieu River | morphological | ROM:Ich:BCF-0449-3 | BCF-0449-3 | EU524569 |
| Esox americanus | Canada: Quebec: Richelieu River | morphological | ROM:Ich:BCF-0449-2 | BCF-0449-2 | EU524570 |
| Esox americanus | Canada: Quebec: Richelieu River | morphological | ROM:Ich:BCF-0449-1 | BCF-0449-1 | EU524571 |
| Esox americanus | Canada: Ontario: Tea Creek | morphological | ROM:Ich:BCF-0430-2 | BCF-0430-2 | EU524572 |
| Esox americanus | Canada: Ontario: Tea Creek | morphological | ROM:Ich:BCF-0430-1 | BCF-0430-1 | EU524573 |
| Esox americanus | Canada: Quebec: Fleuve St-Laurent, lac St-Pierre | morphological | ROM:Ich:BCF-0046-3 | BCF-0046-3 | EU524574 |
| Esox americanus | Canada: Quebec: Fleuve St-Laurent, lac St-Pierre | morphological | ROM:Ich:BCF-0046-2 | BCF-0046-2 | EU524575 |
| Esox americanus | Canada: Quebec: Fleuve St-Laurent, lac St-Pierre | morphological | ROM:Ich:BCF-0046-1 | BCF-0046-1 | EU524576 |
| Esox americanus | Canada: Quebec: Marais St-Eugene | morphological | ROM:Ich:BCF-0045-2 | BCF-0045-2 | EU524577 |
| Esox lucius | Canada: Quebec: Marais St-Eugene | morphological | ROM:Ich:BCF-0041-1 | BCF-0041-1 | EU524010 |
| Esox lucius | Canada: Ontario: Lake Ontario | morphological | ROM:Ich:BCF-0457-2 | BCF-0457-2 | EU524578 |
| Esox lucius | Canada: Ontario: Lake Ontario | morphological | ROM:Ich:BCF-0457-1 | BCF-0457-1 | EU524579 |
| Esox lucius | Canada: Ontario: Lake Ontario | morphological | ROM:Ich:BCF-0455-2 | BCF-0455-2 | EU524580 |
| Esox lucius | Canada: Ontario: Lake Ontario | morphological | ROM:Ich:BCF-0455-1 | BCF-0455-1 | EU524581 |
| Esox lucius | Canada: Quebec: Fleuve St-Laurent, riviere St-Jean | morphological | ROM:Ich:BCF-0294-3 | BCF-0294-3 | EU524582 |
| Esox lucius | Canada: Quebec: Fleuve St-Laurent, riviere St-Jean | morphological | ROM:Ich:BCF-0294-2 | BCF-0294-2 | EU524583 |
| Esox lucius | Canada: Quebec: Riviere Gatineau | morphological | ROM:Ich:BCF-0280-3 | BCF-0280-3 | EU524584 |
| Esox lucius | Canada: Quebec: Riviere Gatineau | morphological | ROM:Ich:BCF-0280-2 | BCF-0280-2 | EU524585 |
| Esox lucius | Canada: Quebec: Riviere Gatineau | morphological | ROM:Ich:BCF-0280-1 | BCF-0280-1 | EU524586 |
| Esox lucius | Canada: Ontario: Lac Opinicon | tissue | UOG:Bio:BCF-0218-3 | BCF-0218-3 | EU524587 |
| Esox lucius | Canada: Ontario: Lac Opinicon | tissue | UOG:Bio:BCF-0218-2 | BCF-0218-2 | EU524588 |
| Esox lucius | Canada: Ontario: Lac Opinicon | tissue | UOG:Bio:BCF-0218-1 | BCF-0218-1 | EU524589 |
| Esox lucius | Canada: Quebec: Fleuve St-Laurent, Lac St-Pierre | tissue | UOG:Bio:BCF-0040-3 | BCF-0040-3 | EU524590 |
| Esox lucius | Canada: Quebec: Fleuve St-Laurent, Lac St-Pierre | tissue | UOG:Bio:BCF-0040-2 | BCF-0040-2 | EU524591 |
| Esox lucius | Canada: Quebec: Fleuve St-Laurent, Lac St-Pierre | tissue | UOG:Bio:BCF-0040-1 | BCF-0040-1 | EU524592 |
| Esox masquinongy | Canada: Quebec: Riviere a la truite | morphological | ROM:Ich:BCF-0038-1 | BCF-0038-1 | EU524011 |
| Esox masquinongy | Canada: Ontario: Muskie Lake | morphological | ROM:Ich:BCF-0448-2 | BCF-0448-2 | EU524593 |
| Esox masquinongy | Canada: Ontario: Stony Lake | morphological | ROM:Ich:BCF-0448-10 | BCF-0448-10 | EU524594 |
| Esox masquinongy | Canada: Ontario: Muskie Lake | morphological | ROM:Ich:BCF-0448-1 | BCF-0448-1 | EU524595 |
| Esox masquinongy | Canada: Quebec: Fleuve St-Laurent, lac St-Louis | morphological | ROM:Ich:BCF-0329-1 | BCF-0329-1 | EU524596 |
| Esox masquinongy | Canada: Quebec: Fleuve St-Laurent, lac St-Louis | morphological | ROM:Ich:BCF-0039-2 | BCF-0039-2 | EU524597 |
| Esox masquinongy | Canada: Quebec: Fleuve St-Laurent, lac St-Louis | morphological | ROM:Ich:BCF-0039-1 | BCF-0039-1 | EU524598 |
| Esox masquinongy | Canada: Ontario: Georgian Bay | morphological | ROM:Ich:BCF-0500-1 | BCF-0500-1 | EU524599 |
| Esox masquinongy | Canada: Ontario: Stony Lake | morphological | ROM:Ich:BCF-0448-9 | BCF-0448-9 | EU524600 |
| Esox masquinongy | Canada: Ontario: Pigeon Lake | morphological | ROM:Ich:BCF-0448-7 | BCF-0448-7 | EU524601 |
| Esox masquinongy | Canada: Ontario: Chemong Lake | morphological | ROM:Ich:BCF-0448-6 | BCF-0448-6 | EU524602 |
| Esox niger | Canada: Quebec: Lac Stoke | morphological | ROM:Ich:BCF-0199-1 | BCF-0199-1 | EU524012 |
| Esox niger | Canada: New Brunswick: Belleisle Bay | tissue | UOG:Bio:BCF-0580-4 | BCF-0580-4 | EU524603 |
| Esox niger | Canada: New Brunswick: Belleisle Bay | tissue | UOG:Bio:BCF-0580-3 | BCF-0580-3 | EU524604 |
| Esox niger | Canada: New Brunswick: Belleisle Bay | tissue | UOG:Bio:BCF-0580-2 | BCF-0580-2 | EU524605 |
| Esox niger | Canada: New Brunswick: Belleisle Bay | tissue | UOG:Bio:BCF-0580-1 | BCF-0580-1 | EU524606 |
| Esox niger | Canada: Quebec: Ruisseau Noir | morphological | ROM:Ich:BCF-0485-13 | BCF-0485-13 | EU524607 |
| Esox niger | Canada: Quebec: Ruisseau Noir | morphological | ROM:Ich:BCF-0485-12 | BCF-0485-12 | EU524608 |
| Esox niger | Canada: Quebec: Ruisseau Noir | morphological | ROM:Ich:BCF-0485-11 | BCF-0485-11 | EU524609 |
| Esox niger | Canada: Quebec: Ruisseau Noir | morphological | ROM:Ich:BCF-0485-10 | BCF-0485-10 | EU524610 |
| Esox niger | Canada: Quebec: Ruisseau Noir | morphological | ROM:Ich:BCF-0485-1 | BCF-0485-1 | EU524611 |
| Esox niger | Canada: Quebec: lac Stoke | morphological | ROM:Ich:BCF-0199-2 | BCF-0199-2 | EU524612 |
| Fundulus diaphanus | Canada: Ontario: Lac Opinicon | morphological | ROM:Ich:BCF-0221-1 | BCF-0221-1 | EU524058 |
| Fundulus diaphanus | Canada: Nova Scotia: Little Mushamush lake | morphological | ROM:Ich:BCF-0589-3 | BCF-0589-3 | EU524617 |
| Fundulus diaphanus | Canada: Nova Scotia: Little Mushamush lake | morphological | ROM:Ich:BCF-0589-2 | BCF-0589-2 | EU524618 |
| Fundulus diaphanus | Canada: Nova Scotia: Little Mushamush lake | morphological | ROM:Ich:BCF-0589-1 | BCF-0589-1 | EU524619 |
| Fundulus diaphanus | Canada: Ontario: Fleuve St-Laurent | morphological | ROM:Ich:BCF-0349-3 | BCF-0349-3 | EU524620 |
| Fundulus diaphanus | Canada: Ontario: Fleuve St-Laurent | morphological | ROM:Ich:BCF-0349-1 | BCF-0349-1 | EU524621 |
| Fundulus diaphanus | Canada: Ontario: Lac Opinicon | morphological | ROM:Ich:BCF-0221-3 | BCF-0221-3 | EU524622 |
| Fundulus diaphanus | Canada: Ontario: Lac Opinicon | morphological | ROM:Ich:BCF-0221-2 | BCF-0221-2 | EU524623 |
| Fundulus diaphanus | Canada: Quebec: Fleuve St-Laurent, lac St-Pierre | tissue | UOG:Bio:BCF-0129-3 | BCF-0129-3 | EU524624 |
| Fundulus diaphanus | Canada: Quebec: Fleuve St-Laurent, lac St-Pierre | tissue | UOG:Bio:BCF-0129-2 | BCF-0129-2 | EU524625 |
| Fundulus diaphanus | Canada: Quebec: Fleuve St-Laurent, lac St-Pierre | tissue | UOG:Bio:BCF-0129-1 | BCF-0129-1 | EU524626 |
| Fundulus heteroclitus | Canada: Nova Scotia: La Have river | tissue | UOG:Bio:BCF-0588-6 | BCF-0588-6 | EU524627 |
| Fundulus heteroclitus | Canada: Nova Scotia: La Have river | tissue | UOG:Bio:BCF-0588-5 | BCF-0588-5 | EU524628 |
| Fundulus heteroclitus | Canada: Nova Scotia: La Have river | tissue | UOG:Bio:BCF-0588-4 | BCF-0588-4 | EU524629 |
| Fundulus heteroclitus | Canada: Nova Scotia: La Have river | tissue | UOG:Bio:BCF-0588-2 | BCF-0588-2 | EU524630 |
| Fundulus notatus | Canada: Ontario: Sydenham River, Black creek | morphological | ROM:Ich:BCF-0758-7 | BCF-0758-7 | EU524059 |
| Fundulus notatus | Canada: Ontario: Sydenham River, Black creek | morphological | ROM:Ich:BCF-0758-6 | BCF-0758-6 | EU524060 |
| Fundulus notatus | Canada: Ontario: Sydenham River, Black creek | morphological | ROM:Ich:BCF-0758-5 | BCF-0758-5 | EU524061 |
| Fundulus notatus | Canada: Ontario: Sydenham River, Black creek | morphological | ROM:Ich:BCF-0758-4 | BCF-0758-4 | EU524062 |
| Fundulus notatus | Canada: Ontario: Sydenham River, Black creek | morphological | ROM:Ich:BCF-0758-3 | BCF-0758-3 | EU524063 |
| Fundulus notatus | Canada: Ontario: Sydenham River, Black creek | morphological | ROM:Ich:BCF-0758-2 | BCF-0758-2 | EU524064 |
| Fundulus notatus | Canada: Ontario: Sydenham River, Black creek | morphological | ROM:Ich:BCF-0758-1 | BCF-0758-1 | EU524065 |
| Microgadus tomcod | Canada: Quebec: Saint Laurent River | morphological | ROM:Ich:BCF-0883-1 | BCF-0883-1 | EU524129 |
| Microgadus tomcod | United States: Kansas: | tissue | UOG:Bio:BCF-0706-1 | BCF-0706-1 | EU524130 |
| Apeltes quadracus | Canada: Quebec: Trois-Pistol, Saint-Laurent | morphological | ROM:Ich:BCF-0139-1 | BCF-0139-1 | EU523919 |
| Apeltes quadracus | Canada: Quebec: Fleuve St-Laurent, Trois-Pistol | morphological | ROM:Ich:BCF-0139-5 | BCF-0139-5 | EU524443 |
| Apeltes quadracus | Canada: Quebec: Fleuve St-Laurent, Trois-Pistol | morphological | ROM:Ich:BCF-0139-4 | BCF-0139-4 | EU524444 |
| Apeltes quadracus | Canada: Quebec: Fleuve St-Laurent, Trois-Pistol | morphological | ROM:Ich:BCF-0139-3 | BCF-0139-3 | EU524445 |
| Apeltes quadracus | Canada: Quebec: Fleuve St-Laurent, Trois-Pistol | morphological | ROM:Ich:BCF-0139-2 | BCF-0139-2 | EU524446 |
| Culaea inconstans | Canada: Quebec: Riviere Cap-Rouge | morphological | ROM:Ich:BCF-0204-1 | BCF-0204-1 | EU524003 |
| Culaea inconstans | Canada: Ontario: Wanapitei River | morphological | ROM:Ich:BCF-0436-2 | BCF-0436-2 | EU524532 |
| Culaea inconstans | Canada: Ontario: Wanapitei River | morphological | ROM:Ich:BCF-0436-1 | BCF-0436-1 | EU524533 |
| Culaea inconstans | Canada: Quebec: Fleuve St-Laurent, riviere St-Jean | morphological | ROM:Ich:BCF-0296-3 | BCF-0296-3 | EU524534 |
| Culaea inconstans | Canada: Quebec: Fleuve St-Laurent, riviere Cap-Rouge | morphological | ROM:Ich:BCF-0204-3 | BCF-0204-3 | EU524535 |
| Culaea inconstans | Canada: Quebec: Fleuve St-Laurent, riviere Cap-Rouge | morphological | ROM:Ich:BCF-0204-2 | BCF-0204-2 | EU524536 |
| Culaea inconstans | Canada: Quebec: Fleuve St-Laurent, lac St-Louis | tissue | UOG:Bio:BCF-0133-3 | BCF-0133-3 | EU524537 |
| Culaea inconstans | Canada: Quebec: Fleuve St-Laurent, lac St-Louis | tissue | UOG:Bio:BCF-0133-1 | BCF-0133-1 | EU524538 |
| Gasterosteus aculeatus | Canada: Quebec: Riviere Trinite | morphological | ROM:Ich:BCF-0224-1 | BCF-0224-1 | EU524066 |
| Gasterosteus aculeatus | Canada: Quebec: Fleuve St-Laurent, riviere Cap-Rouge | morphological | ROM:Ich:BCF-0135-5 | BCF-0135-5 | EU524631 |
| Gasterosteus aculeatus | Canada: Quebec: Fleuve St-Laurent, riviere Cap-Rouge | morphological | ROM:Ich:BCF-0135-4 | BCF-0135-4 | EU524632 |
| Gasterosteus aculeatus | Canada: Quebec: Fleuve St-Laurent, riviere Cap-Rouge | morphological | ROM:Ich:BCF-0135-3 | BCF-0135-3 | EU524633 |
| Gasterosteus aculeatus | Canada: Quebec: Fleuve St-Laurent, riviere Cap-Rouge | morphological | ROM:Ich:BCF-0135-2 | BCF-0135-2 | EU524634 |
| Gasterosteus aculeatus | Canada: Quebec: Fleuve St-Laurent, riviere Cap-Rouge | morphological | ROM:Ich:BCF-0135-1 | BCF-0135-1 | EU524635 |
| Gasterosteus aculeatus | Canada: Quebec: Fleuve St-Laurent, riviere Malbaie | morphological | ROM:Ich:BCF-0134-4 | BCF-0134-4 | EU524636 |
| Gasterosteus aculeatus | Canada: Quebec: Fleuve St-Laurent, riviere Malbaie | morphological | ROM:Ich:BCF-0134-3 | BCF-0134-3 | EU524637 |
| Gasterosteus aculeatus | Canada: Quebec: Fleuve St-Laurent, riviere Malbaie | morphological | ROM:Ich:BCF-0134-2 | BCF-0134-2 | EU524638 |
| Gasterosteus aculeatus | Canada: Quebec: Fleuve St-Laurent, riviere Malbaie | morphological | ROM:Ich:BCF-0134-1 | BCF-0134-1 | EU524639 |
| Gasterosteus wheatlandi | Canada: Quebec: Ile Verte, Saint-Laurent | morphological | ROM:Ich:BCF-0136-1 | BCF-0136-1 | EU524067 |
| Gasterosteus wheatlandi | Canada: Quebec: Fleuve St-Laurent, Ile Verte | morphological | ROM:Ich:BCF-0136-5 | BCF-0136-5 | EU524640 |
| Gasterosteus wheatlandi | Canada: Quebec: Fleuve St-Laurent, Ile Verte | morphological | ROM:Ich:BCF-0136-3 | BCF-0136-3 | EU524641 |
| Gasterosteus wheatlandi | Canada: Quebec: Fleuve St-Laurent, Ile Verte | morphological | ROM:Ich:BCF-0136-2 | BCF-0136-2 | EU524642 |
| Pungitius pungitius | Canada: British Columbia: Baffin island | tissue | UOG:Bio:BCF-0677-3 | BCF-0677-3 | EU524319 |
| Pungitius pungitius | Canada: British Columbia: Baffin island | tissue | UOG:Bio:BCF-0677-2 | BCF-0677-2 | EU524320 |
| Pungitius pungitius | Canada: Quebec: Ile Verte, Saint-Laurent | morphological | ROM:Ich:BCF-0137-1 | BCF-0137-1 | EU524321 |
| Pungitius pungitius | Canada: Ontario: Lk. Superior-Whitefish Bay | morphological | ROM:Ich:BCF-0340-2 | BCF-0340-2 | EU525105 |
| Pungitius pungitius | Canada: Ontario: Lk. Huron-Meldrum Bay | morphological | ROM:Ich:BCF-0335-1 | BCF-0335-1 | EU525106 |
| Pungitius pungitius | Canada: Ontario: Lk. Superior-Whitefish Bay | morphological | ROM:Ich:BCF-0334-1 | BCF-0334-1 | EU525107 |
| Pungitius pungitius | Canada: Quebec: Fleuve St-Laurent, riviere Ste-Marguerite | morphological | ROM:Ich:BCF-0138-1 | BCF-0138-1 | EU525108 |
| Pungitius pungitius | Canada: Quebec: Fleuve St-Laurent, Ile Verte | morphological | ROM:Ich:BCF-0137-5 | BCF-0137-5 | EU525109 |
| Pungitius pungitius | Canada: Quebec: Fleuve St-Laurent, Ile Verte | morphological | ROM:Ich:BCF-0137-4 | BCF-0137-4 | EU525110 |
| Pungitius pungitius | Canada: Quebec: Fleuve St-Laurent, Ile Verte | morphological | ROM:Ich:BCF-0137-3 | BCF-0137-3 | EU525111 |
| Pungitius pungitius | Canada: Quebec: Fleuve St-Laurent, Ile Verte | morphological | ROM:Ich:BCF-0137-2 | BCF-0137-2 | EU525112 |
| Neogobius melanostomus | Canada: Ontario: Georgian bay | morphological | ROM:Ich:BCF-0775-2 | BCF-0775-2 | EU524154 |
| Neogobius melanostomus | Canada: Ontario: Saint Clair River, McLeod creek | morphological | ROM:Ich:BCF-0761-1 | BCF-0761-1 | EU524155 |
| Neogobius melanostomus | Canada: Quebec: Fleuve Saint-Laurent | morphological | ROM:Ich:BCF-0196-1 | BCF-0196-1 | EU524156 |
| Neogobius melanostomus | Canada: Ontario: Sixteen Mile Creek | tissue | UOG:Bio:BCF-0534-1 | BCF-0534-1 | EU524919 |
| Neogobius melanostomus | Canada: Quebec: Fleuve St-Laurent, | tissue | UOG:Bio:BCF-0196-2 | BCF-0196-2 | EU524920 |
| Proterorhinus marmoratus | Canada: Ontario: Rose Beach, Lac Erie | tissue | UOG:Bio:BCF-0815-5 | BCF-0815-5 | EU524305 |
| Proterorhinus marmoratus | Canada: Ontario: Saint Clair lake, Mitchell Bay | morphological | ROM:Ich:BCF-0766-1 | BCF-0766-1 | EU524306 |
| Proterorhinus marmoratus | Canada: Ontario: Saint Clair lake, Mitchell Bay | morphological | ROM:Ich:BCF-0765-2 | BCF-0765-2 | EU524307 |
| Proterorhinus marmoratus | Canada: Ontario: Rose Beach, Lac Erie | tissue | UOG:Bio:BCF-0815-3 | BCF-0815-3 | EU524308 |
| Proterorhinus marmoratus | Canada: Ontario: Rose Beach, Lac Erie | tissue | UOG:Bio:BCF-0815-2 | BCF-0815-2 | EU524309 |
| Proterorhinus marmoratus | Canada: Ontario: Rose Beach, Lac Erie | tissue | UOG:Bio:BCF-0815-1 | BCF-0815-1 | EU524310 |
| Hiodon alosoides | Canada: Manitoba: Lac Winnipeg | tissue | UOG:Bio:BCF-0321-4 | BCF-0321-4 | EU524646 |
| Hiodon alosoides | Canada: Manitoba: Lac Winnipeg | tissue | UOG:Bio:BCF-0321-2 | BCF-0321-2 | EU524647 |
| Hiodon alosoides | Canada: Quebec: Lac Lamotte | tissue | UOG:Bio:BCF-0022-4 | BCF-0022-4 | EU524648 |
| Hiodon alosoides | Canada: Quebec: Lac Lamotte | tissue | UOG:Bio:BCF-0022-3 | BCF-0022-3 | EU524649 |
| Hiodon alosoides | Canada: Quebec: Lac Lamotte | tissue | UOG:Bio:BCF-0022-2 | BCF-0022-2 | EU524650 |
| Hiodon alosoides | Canada: Quebec: Lac Lamotte | tissue | UOG:Bio:BCF-0022-1 | BCF-0022-1 | EU524651 |
| Hiodon tergisus | Canada: Quebec: Saint Lawrence River St-Nicolas | tissue | UOG:Bio:BCF-0019-1 | BCF-0019-1 | EU524068 |
| Hiodon tergisus | Canada: Ontario: Thames River | morphological | ROM:Ich:BCF-0381-2 | BCF-0381-2 | EU524652 |
| Hiodon tergisus | Canada: Ontario: Thames River | morphological | ROM:Ich:BCF-0381-1 | BCF-0381-1 | EU524653 |
| Hiodon tergisus | Canada: Manitoba: Lac Winnipeg | tissue | UOG:Bio:BCF-0323-3 | BCF-0323-3 | EU524654 |
| Hiodon tergisus | Canada: Quebec: Fleuve St-Laurent, lac St-Louis | tissue | UOG:Bio:BCF-0021-3 | BCF-0021-3 | EU524655 |
| Hiodon tergisus | Canada: Quebec: Fleuve St-Laurent, lac St-Louis | tissue | UOG:Bio:BCF-0021-2 | BCF-0021-2 | EU524656 |
| Hiodon tergisus | Canada: Quebec: Fleuve St-Laurent, lac St-Louis | tissue | UOG:Bio:BCF-0021-1 | BCF-0021-1 | EU524657 |
| Hiodon tergisus | Canada: Quebec: Fleuve St-Laurent, lac St-Pierre | morphological | ROM:Ich:BCF-0020-3 | BCF-0020-3 | EU524658 |
| Hiodon tergisus | Canada: Quebec: Fleuve St-Laurent, lac St-Pierre | morphological | ROM:Ich:BCF-0020-2 | BCF-0020-2 | EU524659 |
| Hiodon tergisus | Canada: Quebec: Fleuve St-Laurent, lac St-Pierre | morphological | ROM:Ich:BCF-0020-1 | BCF-0020-1 | EU524660 |
| Hiodon tergisus | Canada: Quebec: Fleuve St-Laurent, riviere St-Nicolas | tissue | UOG:Bio:BCF-0019-2 | BCF-0019-2 | EU524661 |
| Ameiurus melas | Canada: Ontario: Maitland River | tissue | UOG:Bio:BCF-0774-1 | BCF-0774-1 | EU523905 |
| Ameiurus melas | Canada: Ontario: Saint Clair River, Talford creek | morphological | ROM:Ich:BCF-0759-1 | BCF-0759-1 | EU523906 |
| Ameiurus melas | Canada: British Columbia: Osoyoos lake | morphological | ROM:Ich:BCF-0701-1 | BCF-0701-1 | EU523907 |
| Ameiurus melas | Canada: Ontario: Lake Ontario | morphological | ROM:Ich:BCF-0424-5 | BCF-0424-5 | EU524415 |
| Ameiurus melas | Canada: Ontario: Lake Ontario | morphological | ROM:Ich:BCF-0424-4 | BCF-0424-4 | EU524416 |
| Ameiurus melas | Canada: Ontario: Lake Ontario | morphological | ROM:Ich:BCF-0424-3 | BCF-0424-3 | EU524417 |
| Ameiurus melas | Canada: Ontario: Lake Ontario | morphological | ROM:Ich:BCF-0424-2 | BCF-0424-2 | EU524418 |
| Ameiurus melas | Canada: Ontario: Lake Ontario | morphological | ROM:Ich:BCF-0424-1 | BCF-0424-1 | EU524419 |
| Ameiurus natalis | Canada: Ontario: Sydenham River, Bear creek | morphological | ROM:Ich:BCF-0777-1 | BCF-0777-1 | EU523908 |
| Ameiurus natalis | Canada: Ontario: Tumblesons Pond | morphological | ROM:Ich:BCF-0487-6 | BCF-0487-6 | EU524420 |
| Ameiurus natalis | Canada: Ontario: Tumblesons Pond | morphological | ROM:Ich:BCF-0487-5 | BCF-0487-5 | EU524421 |
| Ameiurus natalis | Canada: Ontario: Tumblesons Pond | morphological | ROM:Ich:BCF-0487-4 | BCF-0487-4 | EU524422 |
| Ameiurus natalis | Canada: Ontario: Tumblesons Pond | morphological | ROM:Ich:BCF-0487-3 | BCF-0487-3 | EU524423 |
| Ameiurus natalis | Canada: Ontario: Tumblesons Pond | morphological | ROM:Ich:BCF-0487-2 | BCF-0487-2 | EU524424 |
| Ameiurus natalis | Canada: Ontario: Tumblesons Pond | morphological | ROM:Ich:BCF-0487-1 | BCF-0487-1 | EU524425 |
| Ameiurus nebulosus | Canada: Quebec: Marais St-Eugene | morphological | ROM:Ich:BCF-0117-1 | BCF-0117-1 | EU523909 |
| Ameiurus nebulosus | Canada: Quebec: Fleuve St-Laurent, riviere Richelieu | tissue | UOG:Bio:BCF-0121-3 | BCF-0121-3 | EU524426 |
| Ameiurus nebulosus | Canada: Quebec: Fleuve St-Laurent, riviere Richelieu | tissue | UOG:Bio:BCF-0121-2 | BCF-0121-2 | EU524427 |
| Ameiurus nebulosus | Canada: Quebec: Fleuve St-Laurent, riviere Richelieu | tissue | UOG:Bio:BCF-0121-1 | BCF-0121-1 | EU524428 |
| Ameiurus nebulosus | Canada: Quebec: Fleuve St-Laurent, lac St-Pierre | morphological | ROM:Ich:BCF-0118-3 | BCF-0118-3 | EU524429 |
| Ameiurus nebulosus | Canada: Quebec: Fleuve St-Laurent, lac St-Pierre | morphological | ROM:Ich:BCF-0118-2 | BCF-0118-2 | EU524430 |
| Ameiurus nebulosus | Canada: Quebec: Fleuve St-Laurent, lac St-Pierre | morphological | ROM:Ich:BCF-0118-1 | BCF-0118-1 | EU524431 |
| Ameiurus nebulosus | Canada: Quebec: Marais St-Eugene | morphological | ROM:Ich:BCF-0117-3 | BCF-0117-3 | EU524432 |
| Ameiurus nebulosus | Canada: Quebec: Marais St-Eugene | morphological | ROM:Ich:BCF-0117-2 | BCF-0117-2 | EU524433 |
| Ictalurus punctatus | Canada: Quebec: Baie Missisquoi | tissue | UOG:Bio:BCF-0113-1 | BCF-0113-1 | EU524106 |
| Ictalurus punctatus | Canada: Quebec: Fleuve St-Laurent, riviere St-Nicolas | morphological | ROM:Ich:BCF-0114-3 | BCF-0114-3 | EU524676 |
| Ictalurus punctatus | Canada: Quebec: Fleuve St-Laurent, riviere St-Nicolas | morphological | ROM:Ich:BCF-0114-2 | BCF-0114-2 | EU524677 |
| Ictalurus punctatus | Canada: Quebec: Fleuve St-Laurent, riviere St-Nicolas | morphological | ROM:Ich:BCF-0114-1 | BCF-0114-1 | EU524678 |
| Ictalurus punctatus | Canada: Ontario: Thames River | morphological | ROM:Ich:BCF-0501-2 | BCF-0501-2 | EU524679 |
| Ictalurus punctatus | Canada: Ontario: Thames River | morphological | ROM:Ich:BCF-0501-1 | BCF-0501-1 | EU524680 |
| Ictalurus punctatus | Canada: Ontario: Thames River | morphological | ROM:Ich:BCF-0394-3 | BCF-0394-3 | EU524681 |
| Ictalurus punctatus | Canada: Ontario: Thames River | morphological | ROM:Ich:BCF-0394-2 | BCF-0394-2 | EU524682 |
| Ictalurus punctatus | Canada: Ontario: Thames River | morphological | ROM:Ich:BCF-0394-1 | BCF-0394-1 | EU524683 |
| Ictalurus punctatus | Canada: Quebec: Fleuve St-Laurent, riviere Richelieu | tissue | UOG:Bio:BCF-0115-3 | BCF-0115-3 | EU524684 |
| Ictalurus punctatus | Canada: Quebec: Fleuve St-Laurent, riviere Richelieu | tissue | UOG:Bio:BCF-0115-2 | BCF-0115-2 | EU524685 |
| Ictalurus punctatus | Canada: Quebec: Fleuve St-Laurent, riviere Richelieu | tissue | UOG:Bio:BCF-0115-1 | BCF-0115-1 | EU524686 |
| Noturus flavus | Canada: Ontario: Humber River | morphological | ROM:Ich:BCF-0481-1 | BCF-0481-1 | EU525039 |
| Noturus flavus | Canada: Ontario: Fansher Creek | morphological | ROM:Ich:BCF-0418-2 | BCF-0418-2 | EU525040 |
| Noturus flavus | Canada: Ontario: Sydenham River | morphological | ROM:Ich:BCF-0418-1 | BCF-0418-1 | EU525041 |
| Noturus flavus | Canada: Ontario: Thames River | morphological | ROM:Ich:BCF-0374-1 | BCF-0374-1 | EU525042 |
| Noturus gyrinus | Canada: Quebec: Marais St-Eugene | morphological | ROM:Ich:BCF-0123-1 | BCF-0123-1 | EU524185 |
| Noturus gyrinus | Canada: Ontario: Long Point NWA | morphological | ROM:Ich:BCF-0372-3 | BCF-0372-3 | EU525043 |
| Noturus gyrinus | Canada: Ontario: Long Point NWA | morphological | ROM:Ich:BCF-0372-2 | BCF-0372-2 | EU525044 |
| Noturus gyrinus | Canada: Ontario: Long Point NWA | morphological | ROM:Ich:BCF-0372-1 | BCF-0372-1 | EU525045 |
| Noturus gyrinus | Canada: Quebec: Fleuve St-Laurent, lac St-Louis | morphological | ROM:Ich:BCF-0124-3 | BCF-0124-3 | EU525046 |
| Noturus gyrinus | Canada: Quebec: Fleuve St-Laurent, lac St-Louis | morphological | ROM:Ich:BCF-0124-2 | BCF-0124-2 | EU525047 |
| Noturus gyrinus | Canada: Quebec: Fleuve St-Laurent, lac St-Louis | morphological | ROM:Ich:BCF-0124-1 | BCF-0124-1 | EU525048 |
| Noturus gyrinus | Canada: Quebec: Marais St-Eugene | morphological | ROM:Ich:BCF-0123-2 | BCF-0123-2 | EU525049 |
| Noturus gyrinus | Canada: Ontario: Twenty Mile Creek | morphological | ROM:Ich:BCF-0464-1 | BCF-0464-1 | EU525050 |
| Noturus gyrinus | Canada: Ontario: Twenty Mile Creek | morphological | ROM:Ich:BCF-0453-2 | BCF-0453-2 | EU525051 |
| Noturus gyrinus | Canada: Ontario: Twenty Mile Creek | morphological | ROM:Ich:BCF-0453-1 | BCF-0453-1 | EU525052 |
| Noturus insignis | Canada: Ontario: Mississippi river | tissue | UOG:Bio:BCF-0732-8 | BCF-0732-8 | EU524186 |
| Noturus insignis | Canada: Ontario: Mississippi river | tissue | UOG:Bio:BCF-0732-6 | BCF-0732-6 | EU524187 |
| Noturus insignis | Canada: Ontario: Mississippi river | tissue | UOG:Bio:BCF-0732-4 | BCF-0732-4 | EU524188 |
| Noturus insignis | Canada: Ontario: Mississippi river | tissue | UOG:Bio:BCF-0732-3 | BCF-0732-3 | EU524189 |
| Noturus miurus | Canada: Ontario: Sydenham River | morphological | ROM:Ich:BCF-0419-1 | BCF-0419-1 | EU525053 |
| Noturus stigmosus | Canada: Ontario: Detroit River | morphological | ROM:Ich:BCF-0493-2 | BCF-0493-2 | EU525054 |
| Noturus stigmosus | Canada: Ontario: Detroit River | morphological | ROM:Ich:BCF-0493-1 | BCF-0493-1 | EU525055 |
| Pylodictis olivaris | Canada: Ontario: Lake St Clair | morphological | ROM:Ich:BCF-0547-2 | BCF-0547-2 | EU525113 |
| Pylodictis olivaris | Canada: Ontario: Lake St Clair | morphological | ROM:Ich:BCF-0547-1 | BCF-0547-1 | EU525114 |
| Lepisosteus oculatus | Canada: Ontario: Lake Erie | morphological | ROM:Ich:BCF-0447-1 | BCF-0447-1 | EU524699 |
| Lepisosteus osseus | Canada: Ontario: Lake Simcoe | morphological | ROM:Ich:BCF-0541-2 | BCF-0541-2 | EU524119 |
| Lepisosteus osseus | Canada: Quebec: Fleuve St-Laurent, Lac St-Pierre | morphological | ROM:Ich:BCF-0012-2 | BCF-0012-2 | EU524120 |
| Lepisosteus osseus | Canada: Quebec: Fleuve St-Laurent, Lac St-Pierre | morphological | ROM:Ich:BCF-0012-1 | BCF-0012-1 | EU524121 |
| Lepisosteus osseus | Canada: Quebec: Riviere Richelieu, Saint-Ours | tissue | UOG:Bio:BCF-0011-1 | BCF-0011-1 | EU524122 |
| Lota lota | Canada: Quebec: Saint-Nicolas, Fleuve Saint-Laurent | tissue | UOG:Bio:BCF-0272-1 | BCF-0272-1 | EU524125 |
| Lota lota | Canada: New Brunswick: Digdegaush lake | morphological | ROM:Ich:BCF-0575-3 | BCF-0575-3 | EU524746 |
| Lota lota | Canada: New Brunswick: Digdegaush lake | morphological | ROM:Ich:BCF-0575-2 | BCF-0575-2 | EU524747 |
| Lota lota | Canada: New Brunswick: Digdegaush lake | morphological | ROM:Ich:BCF-0575-1 | BCF-0575-1 | EU524748 |
| Lota lota | Canada: Quebec: Lac Duparquet | tissue | UOG:Bio:BCF-0564-4 | BCF-0564-4 | EU524749 |
| Lota lota | Canada: Quebec: Lac Duparquet | tissue | UOG:Bio:BCF-0564-3 | BCF-0564-3 | EU524750 |
| Lota lota | Canada: Quebec: Lac Duparquet | tissue | UOG:Bio:BCF-0564-2 | BCF-0564-2 | EU524751 |
| Lota lota | Canada: Quebec: Lac Duparquet | tissue | UOG:Bio:BCF-0564-1 | BCF-0564-1 | EU524752 |
| Lota lota | Canada: Ontario: Lake Erie | morphological | ROM:Ich:BCF-0515-4 | BCF-0515-4 | EU524753 |
| Lota lota | Canada: Ontario: Lake Erie | morphological | ROM:Ich:BCF-0515-3 | BCF-0515-3 | EU524754 |
| Lota lota | Canada: Ontario: Lake Erie | morphological | ROM:Ich:BCF-0515-2 | BCF-0515-2 | EU524755 |
| Lota lota | Canada: Ontario: Lake Erie | morphological | ROM:Ich:BCF-0515-1 | BCF-0515-1 | EU524756 |
| Lota lota | Canada: Quebec: Fleuve St-Laurent, lac St-Louis | morphological | ROM:Ich:BCF-0128-1 | BCF-0128-1 | EU524757 |
| Osmerus mordax | Canada: Ontario: Unknown Creek | morphological | ROM:Ich:BCF-0454-4 | BCF-0454-4 | EU524235 |
| Osmerus mordax | Canada: Ontario: Unknown Creek | morphological | ROM:Ich:BCF-0454-2 | BCF-0454-2 | EU524236 |
| Osmerus mordax | Canada: Ontario: Unknown Creek | morphological | ROM:Ich:BCF-0454-1 | BCF-0454-1 | EU524237 |
| Spirinchus thaleichthys | Canada: British Columbia: Fraser river | tissue | UOG:Bio:BCF-0657-3 | BCF-0657-3 | EU524384 |
| Spirinchus thaleichthys | Canada: British Columbia: Fraser river | tissue | UOG:Bio:BCF-0657-1 | BCF-0657-1 | EU524385 |
| Thaleichthys pacificus | Canada: British Columbia: Vancouver island | tissue | UOG:Bio:BCF-0655-4 | BCF-0655-4 | EU524386 |
| Thaleichthys pacificus | Canada: British Columbia: Vancouver island | tissue | UOG:Bio:BCF-0655-3 | BCF-0655-3 | EU524387 |
| Thaleichthys pacificus | Canada: British Columbia: Vancouver island | tissue | UOG:Bio:BCF-0655-2 | BCF-0655-2 | EU524388 |
| Thaleichthys pacificus | United States: Washington: Columbia river | tissue | UOG:Bio:BCF-0654-1 | BCF-0654-1 | EU524389 |
| Morone americana | Canada: Ontario: Lake Erie | morphological | ROM:Ich:BCF-0503-3 | BCF-0503-3 | EU524133 |
| Morone americana | Canada: Ontario: Lake Erie | morphological | ROM:Ich:BCF-0503-2 | BCF-0503-2 | EU524134 |
| Morone americana | Canada: Ontario: Lake Erie | morphological | ROM:Ich:BCF-0503-1 | BCF-0503-1 | EU524135 |
| Morone americana | Canada: Quebec: Fleuve St-Laurent, riviere Richelieu | tissue | UOG:Bio:BCF-0147-2 | BCF-0147-2 | EU524136 |
| Morone americana | Canada: Quebec: Fleuve St-Laurent, riviere Richelieu | tissue | UOG:Bio:BCF-0147-1 | BCF-0147-1 | EU524137 |
| Morone americana | Canada: Quebec: Fleuve St-Laurent, riviere St-Nicolas | tissue | UOG:Bio:BCF-0146-3 | BCF-0146-3 | EU524138 |
| Morone americana | Canada: Quebec: Saint Lawrence River St-Nicolas | tissue | UOG:Bio:BCF-0146-1 | BCF-0146-1 | EU524139 |
| Morone chrysops | Canada: Ontario: Lake Erie | morphological | ROM:Ich:BCF-0504-3 | BCF-0504-3 | EU524140 |
| Morone chrysops | Canada: Ontario: Lake Erie | morphological | ROM:Ich:BCF-0504-2 | BCF-0504-2 | EU524141 |
| Morone chrysops | Canada: Ontario: Lake Erie | morphological | ROM:Ich:BCF-0504-1 | BCF-0504-1 | EU524142 |
| Morone saxatilis | Canada: Quebec: Pisciculture de Baldwin | tissue | UOG:Bio:BCF-0149-7 | BCF-0149-7 | EU524143 |
| Morone saxatilis | Canada: Quebec: Pisciculture de Baldwin | tissue | UOG:Bio:BCF-0149-6 | BCF-0149-6 | EU524144 |
| Morone saxatilis | Canada: Quebec: Pisciculture de Baldwin | tissue | UOG:Bio:BCF-0149-5 | BCF-0149-5 | EU524145 |
| Ammocrypta pellucida | Canada: Ontario: Grand River | morphological | ROM:Ich:BCF-0391-6 | BCF-0391-6 | EU523911 |
| Ammocrypta pellucida | Canada: Ontario: Grand River | morphological | ROM:Ich:BCF-0391-5 | BCF-0391-5 | EU523912 |
| Ammocrypta pellucida | Canada: Ontario: Grand River | morphological | ROM:Ich:BCF-0391-4 | BCF-0391-4 | EU523913 |
| Ammocrypta pellucida | Canada: Ontario: Grand River | morphological | ROM:Ich:BCF-0391-2 | BCF-0391-2 | EU523914 |
| Ammocrypta pellucida | Canada: Ontario: Grand River | morphological | ROM:Ich:BCF-0391-1 | BCF-0391-1 | EU523915 |
| Ammocrypta pellucida | Canada: Quebec: Fleuve St-Laurent, lac St-Pierre | morphological | ROM:Ich:BCF-0182-2 | BCF-0182-2 | EU523916 |
| Ammocrypta pellucida | Canada: Quebec: Lac Saint-Pierre, Pointe Yamachiche | morphological | ROM:Ich:BCF-0182-1 | BCF-0182-1 | EU523917 |
| Etheostoma blennioides | Canada: Ontario: Sydenham River | morphological | ROM:Ich:BCF-0769-1 | BCF-0769-1 | EU524013 |
| Etheostoma blennioides | Canada: Ontario: Grand River | morphological | ROM:Ich:BCF-0392-6 | BCF-0392-6 | EU524014 |
| Etheostoma blennioides | Canada: Ontario: Grand River | morphological | ROM:Ich:BCF-0392-5 | BCF-0392-5 | EU524015 |
| Etheostoma blennioides | Canada: Ontario: Grand River | morphological | ROM:Ich:BCF-0392-4 | BCF-0392-4 | EU524016 |
| Etheostoma blennioides | Canada: Ontario: Grand River | morphological | ROM:Ich:BCF-0392-3 | BCF-0392-3 | EU524017 |
| Etheostoma blennioides | Canada: Ontario: Grand River | morphological | ROM:Ich:BCF-0392-2 | BCF-0392-2 | EU524018 |
| Etheostoma blennioides | Canada: Ontario: Grand River | morphological | ROM:Ich:BCF-0392-1 | BCF-0392-1 | EU524019 |
| Etheostoma caeruleum | Canada: Ontario: Maitland River | morphological | ROM:Ich:BCF-0771-10 | BCF-0771-10 | EU524020 |
| Etheostoma caeruleum | Canada: Ontario: Maitland River | morphological | ROM:Ich:BCF-0771-1 | BCF-0771-1 | EU524021 |
| Etheostoma caeruleum | Canada: Ontario: Sauble River, Georgian Bay | morphological | ROM:Ich:BCF-0757-3 | BCF-0757-3 | EU524022 |
| Etheostoma caeruleum | Canada: Ontario: Sauble River, Georgian Bay | morphological | ROM:Ich:BCF-0757-2 | BCF-0757-2 | EU524023 |
| Etheostoma exile | Canada: Ontario: Wanapitei River | morphological | ROM:Ich:BCF-0441-4 | BCF-0441-4 | EU524024 |
| Etheostoma exile | Canada: Ontario: Wanapitei River | morphological | ROM:Ich:BCF-0441-3 | BCF-0441-3 | EU524025 |
| Etheostoma exile | Canada: Ontario: Credit River | morphological | ROM:Ich:BCF-0441-2 | BCF-0441-2 | EU524026 |
| Etheostoma exile | Canada: Ontario: Credit River | morphological | ROM:Ich:BCF-0441-1 | BCF-0441-1 | EU524027 |
| Etheostoma exile | Canada: Quebec: Fleuve St-Laurent, lac St-Louis | morphological | ROM:Ich:BCF-0181-3 | BCF-0181-3 | EU524028 |
| Etheostoma exile | Canada: Quebec: Fleuve St-Laurent, lac St-Louis | morphological | ROM:Ich:BCF-0181-2 | BCF-0181-2 | EU524029 |
| Etheostoma exile | Canada: Quebec: Lac Saint-Louis (Fleuve Saint-Laurent) | morphological | ROM:Ich:BCF-0181-1 | BCF-0181-1 | EU524030 |
| Etheostoma flabellare | Canada: Ontario: Maitland River | morphological | ROM:Ich:BCF-0772-3 | BCF-0772-3 | EU524031 |
| Etheostoma flabellare | Canada: Ontario: Maitland River | morphological | ROM:Ich:BCF-0772-2 | BCF-0772-2 | EU524032 |
| Etheostoma flabellare | Canada: Ontario: Maitland River | morphological | ROM:Ich:BCF-0772-10 | BCF-0772-10 | EU524033 |
| Etheostoma flabellare | Canada: Ontario: Maitland River | morphological | ROM:Ich:BCF-0772-1 | BCF-0772-1 | EU524034 |
| Etheostoma flabellare | Canada: Quebec: Fleuve St-Laurent, riviere du Sud | morphological | ROM:Ich:BCF-0178-4 | BCF-0178-4 | EU524035 |
| Etheostoma flabellare | Canada: Quebec: Fleuve St-Laurent, riviere du Sud | morphological | ROM:Ich:BCF-0178-3 | BCF-0178-3 | EU524036 |
| Etheostoma flabellare | Canada: Quebec: Fleuve St-Laurent, riviere du Sud | morphological | ROM:Ich:BCF-0178-2 | BCF-0178-2 | EU524037 |
| Etheostoma flabellare | Canada: Quebec: Riviere du Sud | morphological | ROM:Ich:BCF-0178-1 | BCF-0178-1 | EU524038 |
| Etheostoma microperca | Canada: Ontario: Miller Lake, Georgian Bay | morphological | ROM:Ich:BCF-0764-7 | BCF-0764-7 | EU524039 |
| Etheostoma microperca | Canada: Ontario: Miller Lake, Georgian Bay | morphological | ROM:Ich:BCF-0764-6 | BCF-0764-6 | EU524040 |
| Etheostoma microperca | Canada: Ontario: Miller Lake, Georgian Bay | morphological | ROM:Ich:BCF-0764-4 | BCF-0764-4 | EU524041 |
| Etheostoma microperca | Canada: Ontario: Miller Lake, Georgian Bay | morphological | ROM:Ich:BCF-0764-3 | BCF-0764-3 | EU524042 |
| Etheostoma microperca | Canada: Ontario: Miller Lake, Georgian Bay | morphological | ROM:Ich:BCF-0764-2 | BCF-0764-2 | EU524043 |
| Etheostoma microperca | Canada: Ontario: Miller Lake, Georgian Bay | morphological | ROM:Ich:BCF-0764-1 | BCF-0764-1 | EU524044 |
| Etheostoma nigrum | Canada: Ontario: Thames River | morphological | ROM:Ich:BCF-0573-2 | BCF-0573-2 | EU524045 |
| Etheostoma nigrum | Canada: Ontario: Thames River | morphological | ROM:Ich:BCF-0573-1 | BCF-0573-1 | EU524046 |
| Etheostoma nigrum | Canada: Quebec: Fleuve St-Laurent, riviere St-Jean | morphological | ROM:Ich:BCF-0293-2 | BCF-0293-2 | EU524047 |
| Etheostoma nigrum | Canada: Quebec: Fleuve St-Laurent, riviere St-Jean | morphological | ROM:Ich:BCF-0293-1 | BCF-0293-1 | EU524048 |
| Etheostoma nigrum | Canada: Quebec: Fleuve St-Laurent, riviere du Sud | morphological | ROM:Ich:BCF-0242-3 | BCF-0242-3 | EU524049 |
| Etheostoma nigrum | Canada: Quebec: Fleuve St-Laurent, riviere du Sud | morphological | ROM:Ich:BCF-0242-2 | BCF-0242-2 | EU524050 |
| Etheostoma olmstedi | Canada: Quebec: Fleuve St-Laurent, lac St-Pierre | morphological | ROM:Ich:BCF-0245-3 | BCF-0245-3 | EU524051 |
| Etheostoma olmstedi | Canada: Quebec: Fleuve St-Laurent, lac St-Pierre | morphological | ROM:Ich:BCF-0245-2 | BCF-0245-2 | EU524052 |
| Etheostoma olmstedi | Canada: Quebec: Fleuve St-Laurent, lac St-Pierre | morphological | ROM:Ich:BCF-0245-1 | BCF-0245-1 | EU524053 |
| Etheostoma olmstedi | Canada: Quebec: Fleuve St-Laurent, lac St-Paul | morphological | ROM:Ich:BCF-0244-2 | BCF-0244-2 | EU524054 |
| Etheostoma olmstedi | Canada: Quebec: Fleuve St-Laurent, lac St-Paul | morphological | ROM:Ich:BCF-0244-1 | BCF-0244-1 | EU524055 |
| Etheostoma olmstedi | Canada: Quebec: Fleuve St-Laurent, lac St-Louis | morphological | ROM:Ich:BCF-0180-3 | BCF-0180-3 | EU524056 |
| Gymnocephalus cernuus | United States: : St. Louis River Estuary | tissue | UOG:Bio:FFC71 | FFC71 | EU524643 |
| Gymnocephalus cernuus | United States: : St. Louis River Estuary | tissue | UOG:Bio:FFC74 | FFC74 | EU524644 |
| Gymnocephalus cernuus | United States: : St. Louis River Estuary | tissue | UOG:Bio:FFC90 | FFC90 | EU524645 |
| Perca flavescens | Canada: British Columbia: Charlie lake, Fort Saint-John | tissue | UOG:Bio:BCF-0686-3 | BCF-0686-3 | EU524238 |
| Perca flavescens | Canada: New Brunswick: Gapetown | tissue | UOG:Bio:BCF-0578-2 | BCF-0578-2 | EU524239 |
| Perca flavescens | Canada: Ontario: Lac Opinicon | morphological | ROM:Ich:BCF-0214-3 | BCF-0214-3 | EU524240 |
| Perca flavescens | Canada: Ontario: Lac Opinicon | morphological | ROM:Ich:BCF-0214-2 | BCF-0214-2 | EU524241 |
| Perca flavescens | Canada: Ontario: Lac Opinicon | morphological | ROM:Ich:BCF-0214-1 | BCF-0214-1 | EU524242 |
| Perca flavescens | Canada: Quebec: Baie Missisquoi | morphological | ROM:Ich:BCF-0188-3 | BCF-0188-3 | EU524243 |
| Perca flavescens | Canada: Quebec: Baie Missisquoi | morphological | ROM:Ich:BCF-0188-2 | BCF-0188-2 | EU524244 |
| Perca flavescens | Canada: Quebec: Baie Missisquoi | morphological | ROM:Ich:BCF-0188-1 | BCF-0188-1 | EU524245 |
| Percina caprodes | Canada: Ontario: Wanapitei River | morphological | ROM:Ich:BCF-0446-12 | BCF-0446-12 | EU524246 |
| Percina caprodes | Canada: Ontario: Thames River | morphological | ROM:Ich:BCF-0373-5 | BCF-0373-5 | EU524247 |
| Percina caprodes | Canada: Ontario: Thames River | morphological | ROM:Ich:BCF-0373-4 | BCF-0373-4 | EU524248 |
| Percina caprodes | Canada: Quebec: Riviere Batiscan | morphological | ROM:Ich:BCF-0205-1 | BCF-0205-1 | EU524249 |
| Percina copelandi | Canada: Quebec: Fleuve St-Laurent, riviere Becancour | morphological | ROM:Ich:BCF-0241-3 | BCF-0241-3 | EU524250 |
| Percina copelandi | Canada: Quebec: Fleuve St-Laurent, riviere Becancour | morphological | ROM:Ich:BCF-0241-2 | BCF-0241-2 | EU524251 |
| Percina copelandi | Canada: Quebec: Riviere Becancour | morphological | ROM:Ich:BCF-0241-1 | BCF-0241-1 | EU524252 |
| Percina maculata | Canada: Ontario: Thames River | morphological | ROM:Ich:BCF-0411-1 | BCF-0411-1 | EU524253 |
| Percina maculata | Canada: Ontario: Talford creek, Saint Clair River | morphological | ROM:Ich:BCF-0768-1 | BCF-0768-1 | EU524254 |
| Percina maculata | Canada: Ontario: Sydenham River (East) | tissue | UOG:Bio:BCF-0509-2 | BCF-0509-2 | EU524255 |
| Percina maculata | Canada: Ontario: Sydenham River (East) | tissue | UOG:Bio:BCF-0509-1 | BCF-0509-1 | EU524256 |
| Percina maculata | Canada: Ontario: Thames River | morphological | ROM:Ich:BCF-0370-3 | BCF-0370-3 | EU524257 |
| Percina maculata | Canada: Ontario: Thames River | morphological | ROM:Ich:BCF-0370-2 | BCF-0370-2 | EU524258 |
| Percina maculata | Canada: Ontario: Thames River | morphological | ROM:Ich:BCF-0370-1 | BCF-0370-1 | EU524259 |
| Percina shumardi | Canada: Ontario: Thames River | morphological | ROM:Ich:BCF-0618-1 | BCF-0618-1 | EU524260 |
| Sander canadensis | Canada: Quebec: Fleuve St-Laurent, Lac St-Pierre | tissue | UOG:Bio:BCF-0318-4 | BCF-0318-4 | EU524368 |
| Sander canadensis | Canada: Quebec: Fleuve St-Laurent, Lac St-Pierre | tissue | UOG:Bio:BCF-0318-3 | BCF-0318-3 | EU524369 |
| Sander canadensis | Canada: Quebec: Fleuve St-Laurent, Lac St-Pierre | tissue | UOG:Bio:BCF-0318-2 | BCF-0318-2 | EU524370 |
| Sander canadensis | Canada: Quebec: Fleuve St-Laurent, riviere St-Nicolas | tissue | UOG:Bio:BCF-0190-4 | BCF-0190-4 | EU524371 |
| Sander canadensis | Canada: Quebec: Fleuve St-Laurent, riviere St-Nicolas | tissue | UOG:Bio:BCF-0190-3 | BCF-0190-3 | EU524372 |
| Sander canadensis | Canada: Quebec: Saint Lawrence River St-Nicolas | tissue | UOG:Bio:BCF-0190-1 | BCF-0190-1 | EU524373 |
| Sander vitreus | Canada: Ontario: Lake Erie | morphological | ROM:Ich:BCF-0428-4 | BCF-0428-4 | EU524374 |
| Sander vitreus | Canada: Ontario: Lake Erie | morphological | ROM:Ich:BCF-0428-2 | BCF-0428-2 | EU524375 |
| Sander vitreus | Canada: Ontario: Lake Erie | morphological | ROM:Ich:BCF-0428-1 | BCF-0428-1 | EU524376 |
| Sander vitreus | Canada: Quebec: Fleuve St-Laurent, lac St-Louis | morphological | ROM:Ich:BCF-0192-4 | BCF-0192-4 | EU524377 |
| Sander vitreus | Canada: Quebec: Fleuve St-Laurent, lac St-Louis | morphological | ROM:Ich:BCF-0192-3 | BCF-0192-3 | EU524378 |
| Sander vitreus | Canada: Quebec: Fleuve St-Laurent, lac St-Louis | morphological | ROM:Ich:BCF-0192-2 | BCF-0192-2 | EU524379 |
| Sander vitreus | Canada: Quebec: Lac Saint-Louis (Fleuve Saint-Laurent) | morphological | ROM:Ich:BCF-0192-1 | BCF-0192-1 | EU524380 |
| Percopsis omiscomaycus | Canada: Ontario: Sydenham River | morphological | ROM:Ich:BCF-0422-2 | BCF-0422-2 | EU524261 |
| Percopsis omiscomaycus | Canada: Ontario: Sydenham River | morphological | ROM:Ich:BCF-0422-1 | BCF-0422-1 | EU524262 |
| Percopsis omiscomaycus | Canada: Ontario: Thames River | morphological | ROM:Ich:BCF-0371-3 | BCF-0371-3 | EU524263 |
| Percopsis omiscomaycus | Canada: Ontario: Thames River | morphological | ROM:Ich:BCF-0371-2 | BCF-0371-2 | EU524264 |
| Percopsis omiscomaycus | Canada: Ontario: Thames River | morphological | ROM:Ich:BCF-0371-1 | BCF-0371-1 | EU524265 |
| Percopsis omiscomaycus | Canada: Manitoba: Lac Winnipeg | tissue | UOG:Bio:BCF-0327-2 | BCF-0327-2 | EU524266 |
| Percopsis omiscomaycus | Canada: Quebec: Fleuve St-Laurent, Lac St-Pierre | morphological | ROM:Ich:BCF-0125-3 | BCF-0125-3 | EU524267 |
| Percopsis omiscomaycus | Canada: Quebec: Fleuve St-Laurent, Lac St-Pierre | morphological | ROM:Ich:BCF-0125-2 | BCF-0125-2 | EU524268 |
| Percopsis omiscomaycus | Canada: Quebec: Fleuve Saint-Laurent, Lac St-Pierre | morphological | ROM:Ich:BCF-0125-1 | BCF-0125-1 | EU524269 |
| Ichthyomyzon castaneus | Canada: Ontario: Pere Marquette River, Michigan Lake | tissue | UOG:Bio:BCF-0896-3 | BCF-0896-3 | EU524087 |
| Ichthyomyzon castaneus | Canada: Ontario: Pere Marquette River, Michigan Lake | tissue | UOG:Bio:BCF-0896-2 | BCF-0896-2 | EU524088 |
| Ichthyomyzon castaneus | Canada: Ontario: Pere Marquette River, Michigan Lake | tissue | UOG:Bio:BCF-0896-1 | BCF-0896-1 | EU524089 |
| Ichthyomyzon fossor | Canada: Ontario: Nine Mile River, Huron Lake | tissue | UOG:Bio:BCF-0895-3 | BCF-0895-3 | EU524090 |
| Ichthyomyzon fossor | Canada: Ontario: Nine Mile River, Huron Lake | tissue | UOG:Bio:BCF-0895-1 | BCF-0895-1 | EU524091 |
| Ichthyomyzon fossor | Canada: Ontario: Hog Creek, Huron Lake | tissue | UOG:Bio:BCF-0894-2 | BCF-0894-2 | EU524092 |
| Ichthyomyzon fossor | Canada: Ontario: Hog Creek, Huron Lake | tissue | UOG:Bio:BCF-0894-1 | BCF-0894-1 | EU524093 |
| Ichthyomyzon fossor | Canada: Ontario: Coldwater Creek, Huron Lake | tissue | UOG:Bio:BCF-0893-1 | BCF-0893-1 | EU524094 |
| Ichthyomyzon fossor | Canada: Ontario: Coldwater Creek, Huron Lake | tissue | UOG:Bio:BCF-0893-3 | BCF-0893-3 | EU524095 |
| Ichthyomyzon fossor | Canada: Ontario: Coldwater Creek, Huron Lake | tissue | UOG:Bio:BCF-0893-2 | BCF-0893-2 | EU524096 |
| Ichthyomyzon unicuspis | Canada: Ontario: Lake Erie | morphological | ROM:Ich:BCF-0555-3 | BCF-0555-3 | EU524097 |
| Ichthyomyzon unicuspis | Canada: Ontario: Lake Erie | morphological | ROM:Ich:BCF-0555-2 | BCF-0555-2 | EU524098 |
| Ichthyomyzon unicuspis | Canada: Ontario: Lake Erie | morphological | ROM:Ich:BCF-0555-1 | BCF-0555-1 | EU524099 |
| Ichthyomyzon unicuspis | Canada: Quebec: Fleuve St-Laurent, riviere Richelieu | morphological | ROM:Ich:BCF-0005-4 | BCF-0005-4 | EU524100 |
| Ichthyomyzon unicuspis | Canada: Quebec: Fleuve St-Laurent, riviere Richelieu | morphological | ROM:Ich:BCF-0005-3 | BCF-0005-3 | EU524101 |
| Ichthyomyzon unicuspis | Canada: Quebec: Fleuve St-Laurent, ruisseau Hinchinbrook | morphological | ROM:Ich:BCF-0004-3 | BCF-0004-3 | EU524102 |
| Ichthyomyzon unicuspis | Canada: Quebec: Fleuve St-Laurent, ruisseau Hinchinbrook | morphological | ROM:Ich:BCF-0004-2 | BCF-0004-2 | EU524103 |
| Ichthyomyzon unicuspis | Canada: Quebec: Riviere Richelieu, Saint-Ours | morphological | ROM:Ich:BCF-0005-1 | BCF-0005-1 | EU524104 |
| Ichthyomyzon unicuspis | Canada: Quebec: Ruisseau Hinchinbrook | morphological | ROM:Ich:BCF-0004-1 | BCF-0004-1 | EU524105 |
| Lampetra appendix | Canada: Quebec: Fleuve St-Laurent, riviere Malbaie | morphological | ROM:Ich:BCF-0007-3 | BCF-0007-3 | EU524109 |
| Lampetra appendix | Canada: Quebec: Fleuve St-Laurent, riviere Malbaie | morphological | ROM:Ich:BCF-0007-2 | BCF-0007-2 | EU524110 |
| Lampetra appendix | Canada: Quebec: Fleuve St-Laurent, riviere Malbaie | morphological | ROM:Ich:BCF-0007-1 | BCF-0007-1 | EU524111 |
| Lampetra appendix | Canada: Quebec: Fleuve St-Laurent, ruisseau Hinchinbrook | morphological | ROM:Ich:BCF-0003-2 | BCF-0003-2 | EU524112 |
| Lampetra appendix | Canada: Quebec: Fleuve St-Laurent, ruisseau Hinchinbrook | morphological | ROM:Ich:BCF-0003-1 | BCF-0003-1 | EU524113 |
| Lampetra appendix | Canada: Quebec: Fleuve St-Laurent, riviere a la truite | morphological | ROM:Ich:BCF-0002-2 | BCF-0002-2 | EU524114 |
| Lampetra appendix | Canada: Quebec: Fleuve St-Laurent, riviere a la truite | morphological | ROM:Ich:BCF-0002-1 | BCF-0002-1 | EU524115 |
| Lampetra appendix | Canada: Quebec: Fleuve St-Laurent, riviere du Sud | morphological | ROM:Ich:BCF-0001-3 | BCF-0001-3 | EU524116 |
| Lampetra appendix | Canada: Quebec: Fleuve St-Laurent, riviere du Sud | morphological | ROM:Ich:BCF-0001-2 | BCF-0001-2 | EU524117 |
| Lampetra appendix | Canada: Quebec: Riviere du Sud | morphological | ROM:Ich:BCF-0001-1 | BCF-0001-1 | EU524118 |
| Petromyzon marinus | Canada: Quebec: Fleuve St-Laurent, riviere St-Nicolas | tissue | UOG:Bio:BCF-0008-1 | BCF-0008-1 | EU524270 |
| Petromyzon marinus | Canada: Quebec: Fleuve St-Laurent, riviere Ste-Marguerite | morphological | ROM:Ich:BCF-0006-3 | BCF-0006-3 | EU524271 |
| Petromyzon marinus | Canada: Quebec: Fleuve St-Laurent, riviere Ste-Marguerite | morphological | ROM:Ich:BCF-0006-2 | BCF-0006-2 | EU524272 |
| Petromyzon marinus | Canada: Quebec: Fleuve St-Laurent, riviere Ste-Marguerite | morphological | ROM:Ich:BCF-0006-1 | BCF-0006-1 | EU524273 |
| Platichthys flesus | Canada: Ontario: Lake Erie | morphological | ROM:Ich:BCF-0527-1 | BCF-0527-1 | EU524278 |
| Platichthys flesus | Canada: Ontario: Lake Erie | morphological | ROM:Ich:BCF-0522-1 | BCF-0522-1 | EU524279 |
| Coregonus artedi | Canada: Ontario: Lake Huron | tissue | UOG:Bio:BCF-0568-12 | BCF-0568-12 | EU523939 |
| Coregonus artedi | Canada: Ontario: Lake Huron | tissue | UOG:Bio:BCF-0568-11 | BCF-0568-11 | EU523940 |
| Coregonus artedi | Canada: Ontario: Lake Huron | tissue | UOG:Bio:BCF-0568-1 | BCF-0568-1 | EU523941 |
| Coregonus artedi | Canada: Manitoba: Lac Winnipeg | tissue | UOG:Bio:BCF-0320-4 | BCF-0320-4 | EU523942 |
| Coregonus artedi | Canada: Manitoba: Lac Winnipeg | tissue | UOG:Bio:BCF-0320-3 | BCF-0320-3 | EU523943 |
| Coregonus artedi | Canada: Manitoba: Lac Winnipeg | tissue | UOG:Bio:BCF-0320-2 | BCF-0320-2 | EU523944 |
| Coregonus artedi | Canada: Manitoba: Lac Winnipeg | tissue | UOG:Bio:BCF-0320-1 | BCF-0320-1 | EU523945 |
| Coregonus autumnalis | United States: Alaska: Kaktovik lagoon | tissue | UOG:Bio:BCF-0708-5 | BCF-0708-5 | EU523946 |
| Coregonus autumnalis | United States: Alaska: Kaktovik lagoon | tissue | UOG:Bio:BCF-0708-4 | BCF-0708-4 | EU523947 |
| Coregonus autumnalis | United States: Alaska: Kaktovik lagoon | tissue | UOG:Bio:BCF-0708-3 | BCF-0708-3 | EU523948 |
| Coregonus autumnalis | United States: Alaska: Kaktovik lagoon | tissue | UOG:Bio:BCF-0708-2 | BCF-0708-2 | EU523949 |
| Coregonus autumnalis | United States: Alaska: Kaktovik lagoon | tissue | UOG:Bio:BCF-0708-10 | BCF-0708-10 | EU523950 |
| Coregonus autumnalis | United States: Alaska: Kaktovik lagoon | tissue | UOG:Bio:BCF-0708-1 | BCF-0708-1 | EU523951 |
| Coregonus clupeaformis | Canada: British Columbia: Swan lake | tissue | UOG:Bio:BCF-0627-3 | BCF-0627-3 | EU523952 |
| Coregonus clupeaformis | Canada: British Columbia: Swan lake | tissue | UOG:Bio:BCF-0627-2 | BCF-0627-2 | EU523953 |
| Coregonus clupeaformis | Canada: British Columbia: Swan lake | tissue | UOG:Bio:BCF-0627-1 | BCF-0627-1 | EU523954 |
| Coregonus clupeaformis | Canada: Yukon Territory: Yukon river | tissue | UOG:Bio:BCF-0590-3 | BCF-0590-3 | EU523955 |
| Coregonus clupeaformis | Canada: Yukon Territory: Yukon river | tissue | UOG:Bio:BCF-0590-2 | BCF-0590-2 | EU523956 |
| Coregonus clupeaformis | Canada: Quebec: Fleuve St-Laurent, riviere St-Nicolas | tissue | UOG:Bio:BCF-0269-3 | BCF-0269-3 | EU523957 |
| Coregonus clupeaformis | Canada: Quebec: Fleuve St-Laurent, riviere St-Nicolas | tissue | UOG:Bio:BCF-0269-2 | BCF-0269-2 | EU523958 |
| Coregonus clupeaformis | Canada: Quebec: Fleuve St-Laurent, riviere St-Nicolas | tissue | UOG:Bio:BCF-0269-1 | BCF-0269-1 | EU523959 |
| Coregonus hoyi | Canada: Ontario: Lake Huron | tissue | UOG:Bio:BCF-0570-6 | BCF-0570-6 | EU523960 |
| Coregonus hoyi | Canada: Ontario: Lake Huron | tissue | UOG:Bio:BCF-0570-12 | BCF-0570-12 | EU523961 |
| Coregonus hoyi | Canada: Ontario: Lake Huron | tissue | UOG:Bio:BCF-0570-11 | BCF-0570-11 | EU523962 |
| Coregonus hoyi | Canada: Ontario: Lake Huron | tissue | UOG:Bio:BCF-0570-10 | BCF-0570-10 | EU523963 |
| Coregonus hoyi | Canada: Ontario: Lake Huron | tissue | UOG:Bio:BCF-0570-1 | BCF-0570-1 | EU523964 |
| Coregonus huntsmani | Canada: New Brunswick: |  | ACL12 | ACL12 | EU524489 |
| Coregonus kiyi | Canada: Ontario: Lake Superior | tissue | UOG:Bio:BCF-0613-2 | BCF-0613-2 | EU523965 |
| Coregonus laurettae | United States: Alaska: Yukon river | tissue | UOG:Bio:BCF-0709-10 | BCF-0709-10 | EU523966 |
| Coregonus laurettae | United States: Alaska: Yukon river | tissue | UOG:Bio:BCF-0709-1 | BCF-0709-1 | EU523967 |
| Coregonus laurettae | Canada: Yukon Territory: Tanana river | tissue | UOG:Bio:BCF-0632-3 | BCF-0632-3 | EU523968 |
| Coregonus laurettae | Canada: Yukon Territory: Tanana river | tissue | UOG:Bio:BCF-0632-2 | BCF-0632-2 | EU523969 |
| Coregonus laurettae | Canada: Yukon Territory: Tanana river | tissue | UOG:Bio:BCF-0632-1 | BCF-0632-1 | EU523970 |
| Coregonus laurettae | Canada: Yukon Territory: Yukon river | tissue | UOG:Bio:BCF-0594-3 | BCF-0594-3 | EU523971 |
| Coregonus laurettae | Canada: Yukon Territory: Yukon river | tissue | UOG:Bio:BCF-0594-1 | BCF-0594-1 | EU523972 |
| Coregonus nasus | United States: Alaska: Selawik river, Kotzebve sound | tissue | UOG:Bio:BCF-0710-10 | BCF-0710-10 | EU523973 |
| Coregonus nasus | United States: Alaska: Selawik river, Kotzebve sound | tissue | UOG:Bio:BCF-0710-1 | BCF-0710-1 | EU523974 |
| Coregonus nasus | United States: Alaska: Tanana river | tissue | UOG:Bio:BCF-0626-3 | BCF-0626-3 | EU523975 |
| Coregonus nasus | United States: Alaska: Tanana river | tissue | UOG:Bio:BCF-0626-2 | BCF-0626-2 | EU523976 |
| Coregonus nasus | United States: Alaska: Tanana river | tissue | UOG:Bio:BCF-0626-1 | BCF-0626-1 | EU523977 |
| Coregonus nasus | Canada: Yukon Territory: Yukon river | tissue | UOG:Bio:BCF-0591-3 | BCF-0591-3 | EU523978 |
| Coregonus nasus | Canada: Yukon Territory: Yukon river | tissue | UOG:Bio:BCF-0591-2 | BCF-0591-2 | EU523979 |
| Coregonus nigripinnis | Canada: Ontario: Lake Nipigon | tissue | UOG:Bio:BCF-0614-2 | BCF-0614-2 | EU523980 |
| Coregonus nigripinnis | Canada: Ontario: Lake Nipigon | tissue | UOG:Bio:BCF-0614-1 | BCF-0614-1 | EU523981 |
| Coregonus sardinella | Canada: Ontario: Lake Huron | tissue | UOG:Bio:BCF-0567-11 | BCF-0567-11 | EU523982 |
| Coregonus sardinella | Canada: Ontario: Lake Huron | tissue | UOG:Bio:BCF-0567-10 | BCF-0567-10 | EU523983 |
| Coregonus sardinella | Canada: British Columbia: Atlin lake | tissue | UOG:Bio:BCF-0631-2 | BCF-0631-2 | EU523984 |
| Coregonus sardinella | Canada: British Columbia: Atlin lake | tissue | UOG:Bio:BCF-0631-1 | BCF-0631-1 | EU523985 |
| Coregonus sardinella | Canada: Yukon Territory: 6 miles river | tissue | UOG:Bio:BCF-0630-2 | BCF-0630-2 | EU523986 |
| Coregonus sardinella | Canada: Yukon Territory: Yukon river | tissue | UOG:Bio:BCF-0593-1 | BCF-0593-1 | EU523987 |
| Coregonus zenithicus | Canada: Ontario: Lake Nipigon | tissue | UOG:Bio:BCF-0615-2 | BCF-0615-2 | EU523988 |
| Coregonus zenithicus | Canada: Ontario: Lake Nipigon | tissue | UOG:Bio:BCF-0615-1 | BCF-0615-1 | EU523989 |
| Coregonus zenithicus | Canada: Ontario: Lake Huron | tissue | UOG:Bio:BCF-0567-12 | BCF-0567-12 | EU523990 |
| Oncorhynchus clarki | Canada: British Columbia: Parc Ouest | tissue | UOG:Bio:BCF-0604-2 | BCF-0604-2 | EU524190 |
| Oncorhynchus clarki | Canada: British Columbia: Parc Ouest | tissue | UOG:Bio:BCF-0604-1 | BCF-0604-1 | EU524191 |
| Oncorhynchus clarki | Canada: British Columbia: Dewar creek | tissue | UOG:Bio:BCF-0624-2 | BCF-0624-2 | EU524192 |
| Oncorhynchus clarki | Canada: British Columbia: Dewar creek | tissue | UOG:Bio:BCF-0624-1 | BCF-0624-1 | EU524193 |
| Oncorhynchus clarki | Canada: British Columbia: Bull river | tissue | UOG:Bio:BCF-0623-2 | BCF-0623-2 | EU524194 |
| Oncorhynchus clarki | Canada: British Columbia: Bull river | tissue | UOG:Bio:BCF-0623-1 | BCF-0623-1 | EU524195 |
| Oncorhynchus clarki | Canada: British Columbia: Mayer Lake | tissue | UOG:Bio:BCF-0622-2 | BCF-0622-2 | EU524196 |
| Oncorhynchus clarki | Canada: British Columbia: Mayer Lake | tissue | UOG:Bio:BCF-0622-1 | BCF-0622-1 | EU524197 |
| Oncorhynchus clarki | Canada: British Columbia: Chonat lake, Quadra Island | tissue | UOG:Bio:BCF-0621-2 | BCF-0621-2 | EU524198 |
| Oncorhynchus clarki | Canada: British Columbia: Chonat lake, Quadra Island | tissue | UOG:Bio:BCF-0621-1 | BCF-0621-1 | EU524199 |
| Oncorhynchus clarki | Canada: British Columbia: Parc Ouest | tissue | UOG:Bio:BCF-0603-2 | BCF-0603-2 | EU524200 |
| Oncorhynchus clarki | Canada: British Columbia: Parc Ouest | tissue | UOG:Bio:BCF-0603-1 | BCF-0603-1 | EU524201 |
| Oncorhynchus gorbuscha | Canada: British Columbia: Indian River | tissue | UOG:Bio:BCF-0818-8 | BCF-0818-8 | EU524202 |
| Oncorhynchus gorbuscha | Canada: British Columbia: Indian River | tissue | UOG:Bio:BCF-0818-7 | BCF-0818-7 | EU524203 |
| Oncorhynchus gorbuscha | Canada: British Columbia: Indian River | tissue | UOG:Bio:BCF-0818-6 | BCF-0818-6 | EU524204 |
| Oncorhynchus gorbuscha | Canada: British Columbia: Indian River | tissue | UOG:Bio:BCF-0818-5 | BCF-0818-5 | EU524205 |
| Oncorhynchus gorbuscha | Canada: British Columbia: Indian River | tissue | UOG:Bio:BCF-0818-4 | BCF-0818-4 | EU524206 |
| Oncorhynchus gorbuscha | Canada: British Columbia: Indian River | tissue | UOG:Bio:BCF-0818-3 | BCF-0818-3 | EU524207 |
| Oncorhynchus gorbuscha | Canada: British Columbia: Indian River | tissue | UOG:Bio:BCF-0818-2 | BCF-0818-2 | EU524208 |
| Oncorhynchus gorbuscha | Canada: British Columbia: Indian River | tissue | UOG:Bio:BCF-0818-1 | BCF-0818-1 | EU524209 |
| Oncorhynchus keta | Canada: British Columbia: |  | ACL96 | ACL96 | EU525056 |
| Oncorhynchus keta | Canada: British Columbia: |  | ACL95 | ACL95 | EU525057 |
| Oncorhynchus kisutch | Canada: British Columbia: Big Qualicum | tissue | UOG:Bio:BCF-0819-8 | BCF-0819-8 | EU524210 |
| Oncorhynchus kisutch | Canada: British Columbia: Big Qualicum | tissue | UOG:Bio:BCF-0819-7 | BCF-0819-7 | EU524211 |
| Oncorhynchus kisutch | Canada: British Columbia: Big Qualicum | tissue | UOG:Bio:BCF-0819-6 | BCF-0819-6 | EU524212 |
| Oncorhynchus kisutch | Canada: British Columbia: Big Qualicum | tissue | UOG:Bio:BCF-0819-5 | BCF-0819-5 | EU524213 |
| Oncorhynchus kisutch | Canada: British Columbia: Big Qualicum | tissue | UOG:Bio:BCF-0819-4 | BCF-0819-4 | EU524214 |
| Oncorhynchus kisutch | Canada: British Columbia: Big Qualicum | tissue | UOG:Bio:BCF-0819-2 | BCF-0819-2 | EU524215 |
| Oncorhynchus kisutch | Canada: British Columbia: Big Qualicum | tissue | UOG:Bio:BCF-0819-1 | BCF-0819-1 | EU524216 |
| Oncorhynchus mykiss | Canada: Ontario: Welland river, city of welland | tissue | UOG:Bio:BCF-0725-1 | BCF-0725-1 | EU524217 |
| Oncorhynchus mykiss | Canada: British Columbia: Eutsuke lake | tissue | UOG:Bio:BCF-0635-3 | BCF-0635-3 | EU524218 |
| Oncorhynchus mykiss | Canada: British Columbia: Eutsuke lake | tissue | UOG:Bio:BCF-0635-2 | BCF-0635-2 | EU524219 |
| Oncorhynchus mykiss | Canada: British Columbia: Eutsuke lake | tissue | UOG:Bio:BCF-0635-1 | BCF-0635-1 | EU524220 |
| Oncorhynchus mykiss | Canada: Quebec: Fleuve St-Laurent, riviere St-Nicolas | morphological | ROM:Ich:BCF-0033-2 | BCF-0033-2 | EU524221 |
| Oncorhynchus mykiss | Canada: Quebec: Fleuve St-Laurent, riviere St-Nicolas | morphological | ROM:Ich:BCF-0033-1 | BCF-0033-1 | EU524222 |
| Oncorhynchus nerka | Canada: British Columbia: Babine lake | tissue | UOG:Bio:BCF-0648-5 | BCF-0648-5 | EU524223 |
| Oncorhynchus nerka | Canada: British Columbia: Babine lake | tissue | UOG:Bio:BCF-0648-4 | BCF-0648-4 | EU524224 |
| Oncorhynchus nerka | Canada: British Columbia: Babine lake | tissue | UOG:Bio:BCF-0648-3 | BCF-0648-3 | EU524225 |
| Oncorhynchus nerka | Canada: British Columbia: Babine lake | tissue | UOG:Bio:BCF-0648-1 | BCF-0648-1 | EU524226 |
| Oncorhynchus tshawytscha | Canada: British Columbia: Big Qualicum River | tissue | UOG:Bio:BCF-0813-3 | BCF-0813-3 | EU524227 |
| Oncorhynchus tshawytscha | Canada: British Columbia: Big Qualicum River | tissue | UOG:Bio:BCF-0813-2 | BCF-0813-2 | EU524228 |
| Oncorhynchus tshawytscha | Canada: British Columbia: Big Qualicum River | tissue | UOG:Bio:BCF-0813-1 | BCF-0813-1 | EU524229 |
| Oncorhynchus tshawytscha | Canada: British Columbia: Harrison River | tissue | UOG:Bio:BCF-0813-8 | BCF-0813-8 | EU524230 |
| Oncorhynchus tshawytscha | Canada: British Columbia: Harrison River | tissue | UOG:Bio:BCF-0813-7 | BCF-0813-7 | EU524231 |
| Oncorhynchus tshawytscha | Canada: British Columbia: Harrison River | tissue | UOG:Bio:BCF-0813-6 | BCF-0813-6 | EU524232 |
| Oncorhynchus tshawytscha | Canada: British Columbia: Harrison River | tissue | UOG:Bio:BCF-0813-5 | BCF-0813-5 | EU524233 |
| Oncorhynchus tshawytscha | Canada: British Columbia: Big Qualicum River | tissue | UOG:Bio:BCF-0813-4 | BCF-0813-4 | EU524234 |
| Prosopium coulterii | Canada: British Columbia: | tissue | ACL186 | ACL186 | EU525103 |
| Prosopium coulterii | Canada: British Columbia: | tissue | ACL34 | ACL34 | EU525104 |
| Prosopium cylindraceum | Canada: British Columbia: Atlin lake | tissue | UOG:Bio:BCF-0680-3 | BCF-0680-3 | EU524288 |
| Prosopium cylindraceum | Canada: British Columbia: Atlin lake | tissue | UOG:Bio:BCF-0680-2 | BCF-0680-2 | EU524289 |
| Prosopium cylindraceum | Canada: British Columbia: Atlin lake | tissue | UOG:Bio:BCF-0680-1 | BCF-0680-1 | EU524290 |
| Prosopium cylindraceum | Canada: British Columbia: MacDonald Lake | tissue | UOG:Bio:BCF-0679-3 | BCF-0679-3 | EU524291 |
| Prosopium cylindraceum | Canada: British Columbia: MacDonald Lake | tissue | UOG:Bio:BCF-0679-2 | BCF-0679-2 | EU524292 |
| Prosopium cylindraceum | Canada: British Columbia: MacDonald Lake | tissue | UOG:Bio:BCF-0679-1 | BCF-0679-1 | EU524293 |
| Prosopium cylindraceum | Canada: Yukon Territory: Yukon river | tissue | UOG:Bio:BCF-0644-2 | BCF-0644-2 | EU524294 |
| Prosopium cylindraceum | Canada: Yukon Territory: Yukon river | tissue | UOG:Bio:BCF-0644-1 | BCF-0644-1 | EU524295 |
| Prosopium cylindraceum | Canada: Yukon Territory: Yukon river | tissue | UOG:Bio:BCF-0645-2 | BCF-0645-2 | EU524296 |
| Prosopium williamsoni | Canada: British Columbia: Burnt river | tissue | UOG:Bio:BCF-0634-4 | BCF-0634-4 | EU524297 |
| Prosopium williamsoni | Canada: British Columbia: Burnt river | tissue | UOG:Bio:BCF-0634-3 | BCF-0634-3 | EU524298 |
| Prosopium williamsoni | Canada: British Columbia: Burnt river | tissue | UOG:Bio:BCF-0634-2 | BCF-0634-2 | EU524299 |
| Prosopium williamsoni | Canada: British Columbia: Burnt river | tissue | UOG:Bio:BCF-0634-1 | BCF-0634-1 | EU524300 |
| Prosopium williamsoni | Canada: British Columbia: Montana lake | tissue | UOG:Bio:BCF-0633-4 | BCF-0633-4 | EU524301 |
| Prosopium williamsoni | Canada: British Columbia: Montana lake | tissue | UOG:Bio:BCF-0633-3 | BCF-0633-3 | EU524302 |
| Prosopium williamsoni | Canada: British Columbia: Montana lake | tissue | UOG:Bio:BCF-0633-2 | BCF-0633-2 | EU524303 |
| Prosopium williamsoni | Canada: British Columbia: Montana lake | tissue | UOG:Bio:BCF-0633-1 | BCF-0633-1 | EU524304 |
| Prosopium williamsoni | Canada: British Columbia: Omenica river | tissue | UOG:Bio:BCF-0688-4 | BCF-0688-4 | EU522439 |
| Prosopium williamsoni | Canada: British Columbia: Omenica river | tissue | UOG:Bio:BCF-0688-3 | BCF-0688-3 | EU522440 |
| Prosopium williamsoni | Canada: British Columbia: Omenica river | tissue | UOG:Bio:BCF-0688-1 | BCF-0688-1 | EU522438 |
| Salmo salar | Canada: Quebec: Fleuve St-Laurent, riviere Ouasiemsca | tissue | UOG:Bio:BCF-0607-3 | BCF-0607-3 | EU524349 |
| Salmo salar | Canada: Quebec: Fleuve St-Laurent, riviere Ouasiemsca | tissue | UOG:Bio:BCF-0607-1 | BCF-0607-1 | EU524350 |
| Salmo salar | Canada: Quebec: Fleuve St-Laurent, riviere Metabetchouane | tissue | UOG:Bio:BCF-0606-4 | BCF-0606-4 | EU524351 |
| Salmo salar | Canada: Quebec: Fleuve St-Laurent, riviere Metabetchouane | tissue | UOG:Bio:BCF-0606-2 | BCF-0606-2 | EU524352 |
| Salmo salar | Canada: Quebec: Fleuve St-Laurent, riviere Metabetchouane | tissue | UOG:Bio:BCF-0606-1 | BCF-0606-1 | EU524353 |
| Salmo trutta | Canada: New Brunswick: Mc Quarrie Brook | tissue | UOG:Bio:BCF-0581-7 | BCF-0581-7 | EU524354 |
| Salmo trutta | Canada: New Brunswick: Mc Quarrie Brook | tissue | UOG:Bio:BCF-0581-6 | BCF-0581-6 | EU524355 |
| Salmo trutta | Canada: New Brunswick: Mc Quarrie Brook | morphological | ROM:Ich:BCF-0581-3 | BCF-0581-3 | EU524356 |
| Salvelinus alpinus | Canada: Quebec: Lac Paul, Parc National Gaspesie | tissue | UOG:Bio:BCF-0598-4 | BCF-0598-4 | EU524357 |
| Salvelinus alpinus | Canada: Quebec: Lac Paul, Parc National Gaspesie | tissue | UOG:Bio:BCF-0598-2 | BCF-0598-2 | EU524358 |
| Salvelinus alpinus | Canada: Quebec: Lac Paul, Parc National Gaspesie | tissue | UOG:Bio:BCF-0598-1 | BCF-0598-1 | EU524359 |
| Salvelinus alpinus | Canada: Quebec: Bald moutain pound | tissue | UOG:Bio:BCF-0597-4 | BCF-0597-4 | EU524360 |
| Salvelinus alpinus | Canada: Quebec: Bald moutain pound | tissue | UOG:Bio:BCF-0597-3 | BCF-0597-3 | EU524361 |
| Salvelinus alpinus | Canada: Quebec: Bald moutain pound | tissue | UOG:Bio:BCF-0597-2 | BCF-0597-2 | EU524362 |
| Salvelinus alpinus | Canada: Quebec: Bald moutain pound | tissue | UOG:Bio:BCF-0597-1 | BCF-0597-1 | EU524363 |
| Salvelinus confluentus | Canada: Quebec: Parc Ouest, Waterton lake | tissue | UOG:Bio:BCF-0612-3 | BCF-0612-3 | EU522398 |
| Salvelinus confluentus | Canada: Quebec: Parc Ouest, Waterton lake | tissue | UOG:Bio:BCF-0612-2 | BCF-0612-2 | EU524364 |
| Salvelinus confluentus | Canada: Quebec: Parc Ouest, Waterton lake | tissue | UOG:Bio:BCF-0612-1 | BCF-0612-1 | EU524365 |
| Salvelinus confluentus | United States: Washington: Yakima | tissue | UOG:Bio:BCF-0638-4 | BCF-0638-4 | EU522399 |
| Salvelinus confluentus | United States: Washington: Yakima | tissue | UOG:Bio:BCF-0638-3 | BCF-0638-3 | EU522400 |
| Salvelinus confluentus | United States: Washington: Yakima | tissue | UOG:Bio:BCF-0638-2 | BCF-0638-2 | EU522401 |
| Salvelinus confluentus | United States: Washington: Yakima | tissue | UOG:Bio:BCF-0638-1 | BCF-0638-1 | EU522402 |
| Salvelinus confluentus | Canada: Quebec: Parc Ouest, Waterton lake | tissue | UOG:Bio:BCF-0612-4 | BCF-0612-4 | EU522403 |
| Salvelinus fontinalis | Canada: Quebec: Fleuve St-Laurent, riviere Wapizagonkec | tissue | UOG:Bio:BCF-0595-3 | BCF-0595-3 | EU522409 |
| Salvelinus fontinalis | Canada: Quebec: Fleuve St-Laurent, riviere Wapizagonkec | tissue | UOG:Bio:BCF-0595-1 | BCF-0595-1 | EU522405 |
| Salvelinus fontinalis | Canada: Quebec: Fleuve St-Laurent, riviere Malbaie | morphological | ROM:Ich:BCF-0031-3 | BCF-0031-3 | EU522406 |
| Salvelinus fontinalis | Canada: Quebec: Fleuve St-Laurent, riviere Malbaie | morphological | ROM:Ich:BCF-0031-2 | BCF-0031-2 | EU522407 |
| Salvelinus fontinalis | Canada: Quebec: Fleuve St-Laurent, riviere Malbaie | morphological | ROM:Ich:BCF-0031-1 | BCF-0031-1 | EU522408 |
| Salvelinus fontinalis | Canada: Quebec: Fleuve St-Laurent, riviere Trinite | morphological | ROM:Ich:BCF-0030-3 | BCF-0030-3 | EU524366 |
| Salvelinus fontinalis | Canada: Quebec: Fleuve St-Laurent, riviere Trinite | morphological | ROM:Ich:BCF-0030-2 | BCF-0030-2 | EU524367 |
| Salvelinus fontinalis | Canada: Quebec: Fleuve St-Laurent, riviere Trinite | morphological | ROM:Ich:BCF-0030-1 | BCF-0030-1 | EU522404 |
| Salvelinus malma | Canada: British Columbia: Chignuk lake | tissue | UOG:Bio:BCF-0641-4 | BCF-0641-4 | EU522410 |
| Salvelinus malma | Canada: British Columbia: Chignuk lake | tissue | UOG:Bio:BCF-0641-3 | BCF-0641-3 | EU522411 |
| Salvelinus malma | Canada: British Columbia: Chignuk lake | tissue | UOG:Bio:BCF-0641-2 | BCF-0641-2 | EU522412 |
| Salvelinus malma | Canada: British Columbia: Chignuk lake | tissue | UOG:Bio:BCF-0641-1 | BCF-0641-1 | EU522413 |
| Salvelinus malma | Canada: British Columbia: Moutain CK | tissue | UOG:Bio:BCF-0640-4 | BCF-0640-4 | EU522414 |
| Salvelinus malma | Canada: British Columbia: Moutain CK | tissue | UOG:Bio:BCF-0640-3 | BCF-0640-3 | EU522415 |
| Salvelinus malma | Canada: British Columbia: Moutain CK | tissue | UOG:Bio:BCF-0640-2 | BCF-0640-2 | EU522416 |
| Salvelinus malma | Canada: British Columbia: Moutain CK | tissue | UOG:Bio:BCF-0640-1 | BCF-0640-1 | EU522417 |
| Salvelinus namaycush | Canada: British Columbia: Minnewanka lake | tissue | UOG:Bio:BCF-0642-4 | BCF-0642-4 | EU522418 |
| Salvelinus namaycush | Canada: British Columbia: Minnewanka lake | tissue | UOG:Bio:BCF-0642-3 | BCF-0642-3 | EU522419 |
| Salvelinus namaycush | Canada: British Columbia: Minnewanka lake | tissue | UOG:Bio:BCF-0642-2 | BCF-0642-2 | EU522420 |
| Salvelinus namaycush | Canada: British Columbia: Minnewanka lake | tissue | UOG:Bio:BCF-0642-1 | BCF-0642-1 | EU522421 |
| Salvelinus namaycush | Canada: Quebec: Lac Simon | tissue | UOG:Bio:BCF-0600-4 | BCF-0600-4 | EU522422 |
| Salvelinus namaycush | Canada: Quebec: Lac Simon | tissue | UOG:Bio:BCF-0600-3 | BCF-0600-3 | EU522423 |
| Salvelinus namaycush | Canada: Quebec: Lac Simon | tissue | UOG:Bio:BCF-0600-2 | BCF-0600-2 | EU522424 |
| Salvelinus namaycush | Canada: Quebec: Lac Simon | tissue | UOG:Bio:BCF-0600-1 | BCF-0600-1 | EU522425 |
| Stenodus leucichthys | Canada: Yukon Territory: Tanana river | tissue | UOG:Bio:BCF-0646-4 | BCF-0646-4 | EU522428 |
| Stenodus leucichthys | Canada: Yukon Territory: Tanana river | tissue | UOG:Bio:BCF-0646-3 | BCF-0646-3 | EU522429 |
| Stenodus leucichthys | Canada: Yukon Territory: Tanana river | tissue | UOG:Bio:BCF-0646-2 | BCF-0646-2 | EU522430 |
| Stenodus leucichthys | Canada: Yukon Territory: Tanana river | tissue | UOG:Bio:BCF-0646-1 | BCF-0646-1 | EU522431 |
| Stenodus leucichthys | Canada: Yukon Territory: Yukon river | tissue | UOG:Bio:BCF-0592-4 | BCF-0592-4 | EU522432 |
| Stenodus leucichthys | Canada: Yukon Territory: Yukon river | tissue | UOG:Bio:BCF-0592-3 | BCF-0592-3 | EU522433 |
| Stenodus leucichthys | Canada: Yukon Territory: Yukon river | tissue | UOG:Bio:BCF-0592-2 | BCF-0592-2 | EU522426 |
| Stenodus leucichthys | Canada: Yukon Territory: Yukon river | tissue | UOG:Bio:BCF-0592-1 | BCF-0592-1 | EU522427 |
| Thymallus arcticus | Canada: British Columbia: Teslin river | tissue | UOG:Bio:BCF-0687-4 | BCF-0687-4 | EU522434 |
| Thymallus arcticus | Canada: British Columbia: Teslin river | tissue | UOG:Bio:BCF-0687-3 | BCF-0687-3 | EU522435 |
| Thymallus arcticus | Canada: British Columbia: Teslin river | tissue | UOG:Bio:BCF-0687-2 | BCF-0687-2 | EU522436 |
| Thymallus arcticus | Canada: British Columbia: Teslin river | tissue | UOG:Bio:BCF-0687-1 | BCF-0687-1 | EU522437 |
| Aplodinotus grunniens | Canada: Ontario: Lake Erie | morphological | ROM:Ich:BCF-0513-3 | BCF-0513-3 | EU522443 |
| Aplodinotus grunniens | Canada: Ontario: Lake Erie | morphological | ROM:Ich:BCF-0513-2 | BCF-0513-2 | EU522444 |
| Aplodinotus grunniens | Canada: Ontario: Lake Erie | morphological | ROM:Ich:BCF-0513-1 | BCF-0513-1 | EU522445 |
| Aplodinotus grunniens | Canada: Manitoba: Lac Winnipeg | tissue | UOG:Bio:BCF-0326-3 | BCF-0326-3 | EU523920 |
| Aplodinotus grunniens | Canada: Manitoba: Lac Winnipeg | tissue | UOG:Bio:BCF-0326-2 | BCF-0326-2 | EU523921 |
| Aplodinotus grunniens | Canada: Manitoba: Lac Winnipeg | tissue | UOG:Bio:BCF-0326-1 | BCF-0326-1 | EU523922 |
| Aplodinotus grunniens | Canada: Quebec: Baie Missisquoi | tissue | UOG:Bio:BCF-0195-3 | BCF-0195-3 | EU522441 |
| Aplodinotus grunniens | Canada: Quebec: Baie Missisquoi | tissue | UOG:Bio:BCF-0195-2 | BCF-0195-2 | EU522442 |
| Aplodinotus grunniens | Canada: Quebec: Baie Missisquoi | tissue | UOG:Bio:BCF-0195-1 | BCF-0195-1 | EU523923 |
| Dallia pectoralis | United States: Alaska: Spring creek, Kenai pennin | morphological | ROM:Ich:BCF-0705-2 | BCF-0705-2 | EU524007 |
| Dallia pectoralis | United States: Alaska: Spring creek, Kenai pennin | morphological | ROM:Ich:BCF-0705-1 | BCF-0705-1 | EU524008 |
| Umbra limi | Canada: Ontario: Tea Creek | morphological | ROM:Ich:BCF-0433-3 | BCF-0433-3 | EU522446 |
| Umbra limi | Canada: Ontario: Tea Creek | morphological | ROM:Ich:BCF-0433-2 | BCF-0433-2 | EU522447 |
| Umbra limi | Canada: Ontario: Tea Creek | morphological | ROM:Ich:BCF-0433-1 | BCF-0433-1 | EU522448 |
| Umbra limi | Canada: Quebec: Fleuve St-Laurent, Lac St-Pierre | morphological | ROM:Ich:BCF-0036-3 | BCF-0036-3 | EU522449 |
| Umbra limi | Canada: Quebec: Fleuve St-Laurent, Lac St-Pierre | morphological | ROM:Ich:BCF-0036-2 | BCF-0036-2 | EU522450 |
| Umbra limi | Canada: Quebec: Fleuve St-Laurent, Lac St-Pierre | morphological | ROM:Ich:BCF-0036-1 | BCF-0036-1 | EU522451 |
| Umbra limi | Canada: Quebec: Fleuve St-Laurent, lac St-Paul | morphological | ROM:Ich:BCF-0034-3 | BCF-0034-3 | EU522452 |
| Umbra limi | Canada: Quebec: Fleuve St-Laurent, lac St-Paul | morphological | ROM:Ich:BCF-0034-2 | BCF-0034-2 | EU522453 |
| Umbra limi | Canada: Quebec: Lac Saint-Paul | morphological | ROM:Ich:BCF-0034-1 | BCF-0034-1 | EU524391 |
