# Supplementary material for: Identifying Canadian Freshwater Fishes through DNA Barcodes
Source: PLoS One. 2008 Jun 18;3(6):e2490. doi: 10.1371/journal.pone.0002490 (PMC3278308; doi:10.1371/journal.pone.0002490)
Supplement: Appendix S2 — Neighbour-joining tree of 1360 COI sequences from the 190 freshwater fish species sampled as obtained in BOLD, using K2P distances. (0.95 MB PDF) [file pone.0002490.s002.pdf]

BOLD TaxonID Tree

Project	:	Freshwater Fish of Canada (FFC)
Subprojects	:	Barcoding of Canadian freshwater fishes[BCF]
		Barcoding of Canadian freshwater fishes Part II[BCFB]
Date	:	5-February-2008
Data Type	:	Nucleotide
Distance Model	:	Kimura 2 Parameter
Codon Positions	:	1st, 2nd, 3rd
Labels	:	SampleID,
Colorization	:	

Sequence Count	:	1360
Species count	:	190
Genus count	:	85
Family count	:	28
Unidentified	:	0

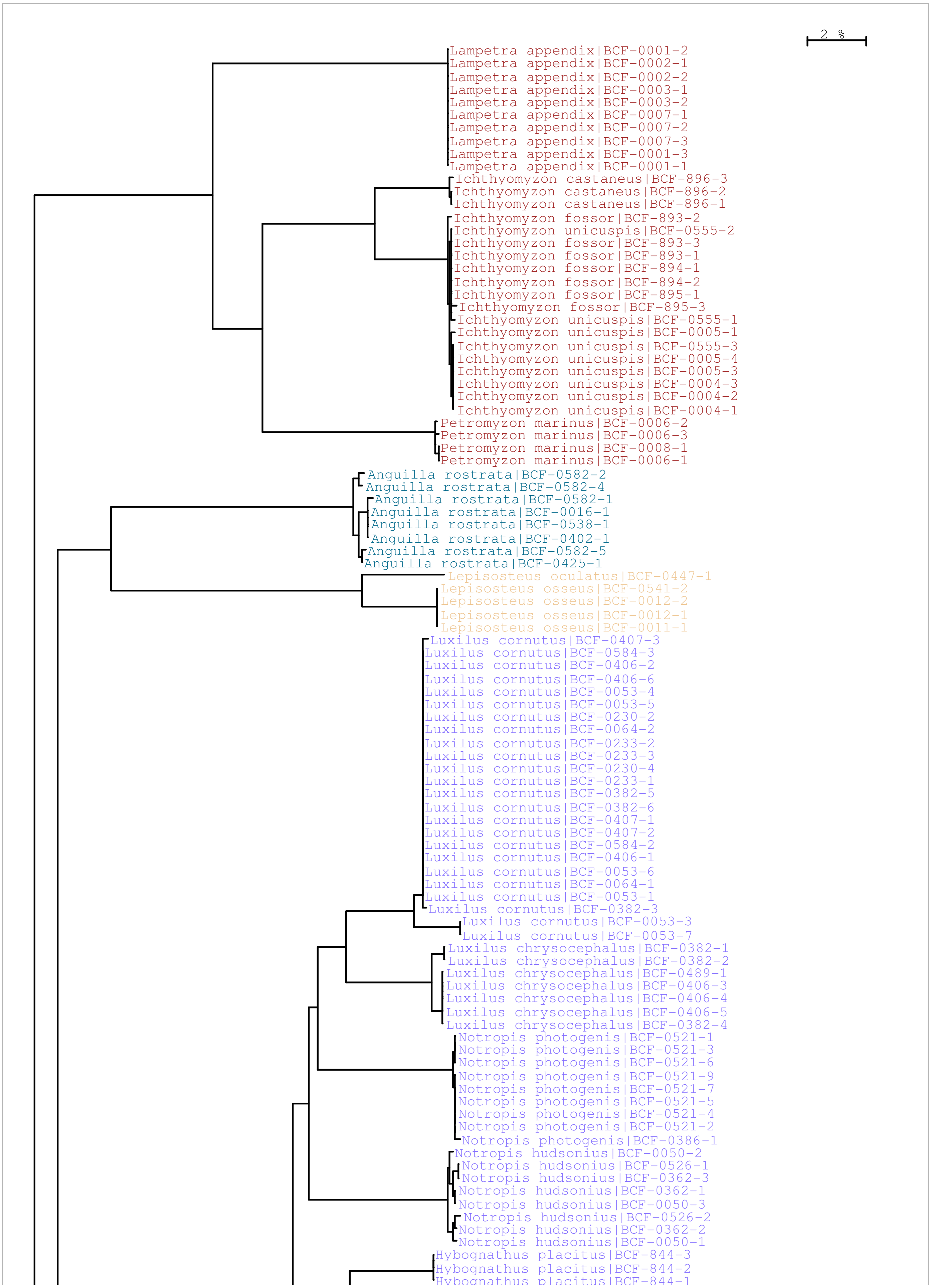
2 %
Lampetra appendix|BCF-0001-2
Lampetra appendix|BCF-0002-1
Lampetra appendix|BCF-0002-2
Lampetra appendix|BCF-0003-1
Lampetra appendix|BCF-0003-2
Lampetra appendix|BCF-0007-1
Lampetra appendix|BCF-0007-2
Lampetra appendix|BCF-0007-3
Lampetra appendix|BCF-0001-3
Lampetra appendix|BCF-0001-1
Ichthyomyzon castaneus|BCF-896-3
Ichthyomyzon castaneus|BCF-896-2
Ichthyomyzon castaneus|BCF-896-1
Ichthyomyzon fossor|BCF-893-2
Ichthyomyzon unicuspis|BCF-0555-2
Ichthyomyzon fossor|BCF-893-3
Ichthyomyzon fossor|BCF-893-1
Ichthyomyzon fossor|BCF-894-1
Ichthyomyzon fossor|BCF-894-2
Ichthyomyzon fossor|BCF-895-1
Ichthyomyzon fossor|BCF-895-3
Ichthyomyzon unicuspis|BCF-0555-1
Ichthyomyzon unicuspis|BCF-0005-1
Ichthyomyzon unicuspis|BCF-0555-3
Ichthyomyzon unicuspis|BCF-0005-4
Ichthyomyzon unicuspis|BCF-0005-3
Ichthyomyzon unicuspis|BCF-0004-3
Ichthyomyzon unicuspis|BCF-0004-2
Ichthyomyzon unicuspis|BCF-0004-1
Petromyzon marinus|BCF-0006-2
Petromyzon marinus|BCF-0006-3
Petromyzon marinus|BCF-0008-1
Petromyzon marinus|BCF-0006-1
Anguilla rostrata|BCF-0582-2
Anguilla rostrata|BCF-0582-4
Anguilla rostrata|BCF-0582-1
Anguilla rostrata|BCF-0016-1
Anguilla rostrata|BCF-0538-1
Anguilla rostrata|BCF-0402-1
Anguilla rostrata|BCF-0582-5
Anguilla rostrata|BCF-0425-1
Lepisosteus oculatus|BCF-0447-1
Lepisosteus osseus|BCF-0541-2
Lepisosteus osseus|BCF-0012-2
Lepisosteus osseus|BCF-0012-1
Lepisosteus osseus|BCF-0011-1
Luxilus cornutus|BCF-0407-3
Luxilus cornutus|BCF-0584-3
Luxilus cornutus|BCF-0406-2
Luxilus cornutus|BCF-0406-6
Luxilus cornutus|BCF-0053-4
Luxilus cornutus|BCF-0053-5
Luxilus cornutus|BCF-0230-2
Luxilus cornutus|BCF-0064-2
Luxilus cornutus|BCF-0233-2
Luxilus cornutus|BCF-0233-3
Luxilus cornutus|BCF-0230-4
Luxilus cornutus|BCF-0233-1
Luxilus cornutus|BCF-0382-5
Luxilus cornutus|BCF-0382-6
Luxilus cornutus|BCF-0407-1
Luxilus cornutus|BCF-0407-2
Luxilus cornutus|BCF-0584-2
Luxilus cornutus|BCF-0406-1
Luxilus cornutus|BCF-0053-6
Luxilus cornutus|BCF-0064-1
Luxilus cornutus|BCF-0053-1
Luxilus cornutus|BCF-0382-3
Luxilus cornutus|BCF-0053-3
Luxilus cornutus|BCF-0053-7
Luxilus chrysocephalus|BCF-0382-1
Luxilus chrysocephalus|BCF-0382-2
Luxilus chrysocephalus|BCF-0489-1
Luxilus chrysocephalus|BCF-0406-3
Luxilus chrysocephalus|BCF-0406-4
Luxilus chrysocephalus|BCF-0406-5
Luxilus chrysocephalus|BCF-0382-4
Notropis photogenis|BCF-0521-1
Notropis photogenis|BCF-0521-3
Notropis photogenis|BCF-0521-6
Notropis photogenis|BCF-0521-9
Notropis photogenis|BCF-0521-7
Notropis photogenis|BCF-0521-5
Notropis photogenis|BCF-0521-4
Notropis photogenis|BCF-0521-2
Notropis photogenis|BCF-0386-1
Notropis hudsonius|BCF-0050-2
Notropis hudsonius|BCF-0526-1
Notropis hudsonius|BCF-0362-3
Notropis hudsonius|BCF-0362-1
Notropis hudsonius|BCF-0050-3
Notropis hudsonius|BCF-0526-2
Notropis hudsonius|BCF-0362-2
Notropis hudsonius|BCF-0050-1
Hybognathus placitus|BCF-844-3
Hybognathus placitus|BCF-844-2
Hybognathus placitus|BCF-844-1

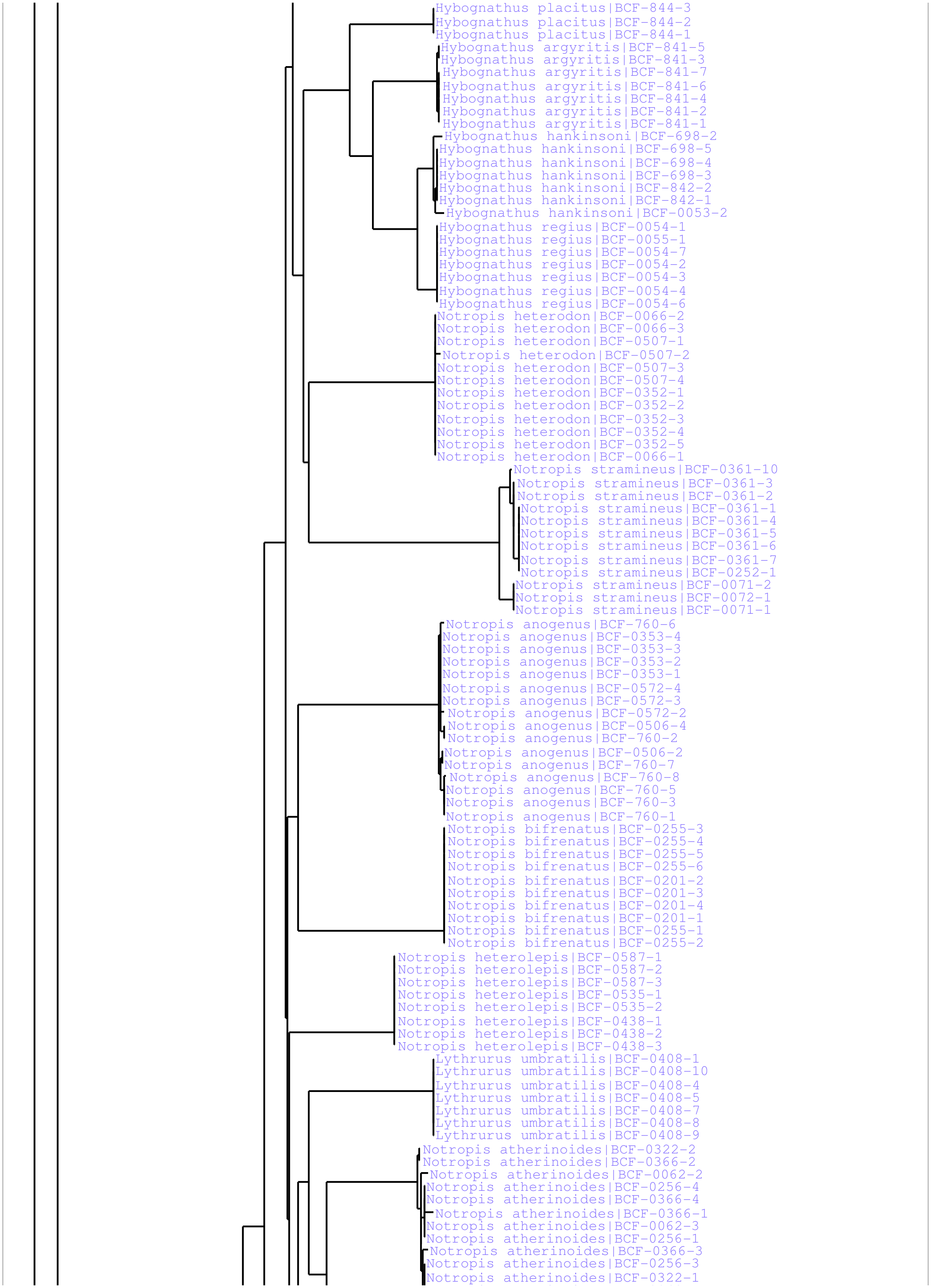
Hybognathus placitus|BCF-844-3
Hybognathus placitus|BCF-844-2
Hybognathus placitus|BCF-844-1
Hybognathus argyritis|BCF-841-5
Hybognathus argyritis|BCF-841-3
Hybognathus argyritis|BCF-841-7
Hybognathus argyritis|BCF-841-6
Hybognathus argyritis|BCF-841-4
Hybognathus argyritis|BCF-841-2
Hybognathus argyritis|BCF-841-1
Hybognathus hankinsoni|BCF-698-2
Hybognathus hankinsoni|BCF-698-5
Hybognathus hankinsoni|BCF-698-4
Hybognathus hankinsoni|BCF-698-3
Hybognathus hankinsoni|BCF-842-2
Hybognathus hankinsoni|BCF-842-1
Hybognathus hankinsoni|BCF-0053-2
Hybognathus regius|BCF-0054-1
Hybognathus regius|BCF-0055-1
Hybognathus regius|BCF-0054-7
Hybognathus regius|BCF-0054-2
Hybognathus regius|BCF-0054-3
Hybognathus regius|BCF-0054-4
Hybognathus regius|BCF-0054-6
Notropis heterodon|BCF-0066-2
Notropis heterodon|BCF-0066-3
Notropis heterodon|BCF-0507-1
Notropis heterodon|BCF-0507-2
Notropis heterodon|BCF-0507-3
Notropis heterodon|BCF-0507-4
Notropis heterodon|BCF-0352-1
Notropis heterodon|BCF-0352-2
Notropis heterodon|BCF-0352-3
Notropis heterodon|BCF-0352-4
Notropis heterodon|BCF-0352-5
Notropis heterodon|BCF-0066-1
Notropis stramineus|BCF-0361-10
Notropis stramineus|BCF-0361-3
Notropis stramineus|BCF-0361-2
Notropis stramineus|BCF-0361-1
Notropis stramineus|BCF-0361-4
Notropis stramineus|BCF-0361-5
Notropis stramineus|BCF-0361-6
Notropis stramineus|BCF-0361-7
Notropis stramineus|BCF-0252-1
Notropis stramineus|BCF-0071-2
Notropis stramineus|BCF-0072-1
Notropis stramineus|BCF-0071-1
Notropis anogenus|BCF-760-6
Notropis anogenus|BCF-0353-4
Notropis anogenus|BCF-0353-3
Notropis anogenus|BCF-0353-2
Notropis anogenus|BCF-0353-1
Notropis anogenus|BCF-0572-4
Notropis anogenus|BCF-0572-3
Notropis anogenus|BCF-0572-2
Notropis anogenus|BCF-0506-4
Notropis anogenus|BCF-760-2
Notropis anogenus|BCF-0506-2
Notropis anogenus|BCF-760-7
Notropis anogenus|BCF-760-8
Notropis anogenus|BCF-760-5
Notropis anogenus|BCF-760-3
Notropis anogenus|BCF-760-1
Notropis bifrenatus|BCF-0255-3
Notropis bifrenatus|BCF-0255-4
Notropis bifrenatus|BCF-0255-5
Notropis bifrenatus|BCF-0255-6
Notropis bifrenatus|BCF-0201-2
Notropis bifrenatus|BCF-0201-3
Notropis bifrenatus|BCF-0201-4
Notropis bifrenatus|BCF-0201-1
Notropis bifrenatus|BCF-0255-1
Notropis bifrenatus|BCF-0255-2
Notropis heterolepis|BCF-0587-1
Notropis heterolepis|BCF-0587-2
Notropis heterolepis|BCF-0587-3
Notropis heterolepis|BCF-0535-1
Notropis heterolepis|BCF-0535-2
Notropis heterolepis|BCF-0438-1
Notropis heterolepis|BCF-0438-2
Notropis heterolepis|BCF-0438-3
Lythrurus umbratilis|BCF-0408-1
Lythrurus umbratilis|BCF-0408-10
Lythrurus umbratilis|BCF-0408-4
Lythrurus umbratilis|BCF-0408-5
Lythrurus umbratilis|BCF-0408-7
Lythrurus umbratilis|BCF-0408-8
Lythrurus umbratilis|BCF-0408-9
Notropis atherinoides|BCF-0322-2
Notropis atherinoides|BCF-0366-2
Notropis atherinoides|BCF-0062-2
Notropis atherinoides|BCF-0256-4
Notropis atherinoides|BCF-0366-4
Notropis atherinoides|BCF-0366-1
Notropis atherinoides|BCF-0062-3
Notropis atherinoides|BCF-0256-1
Notropis atherinoides|BCF-0366-3
Notropis atherinoides|BCF-0256-3
Notropis atherinoides|BCF-0322-1

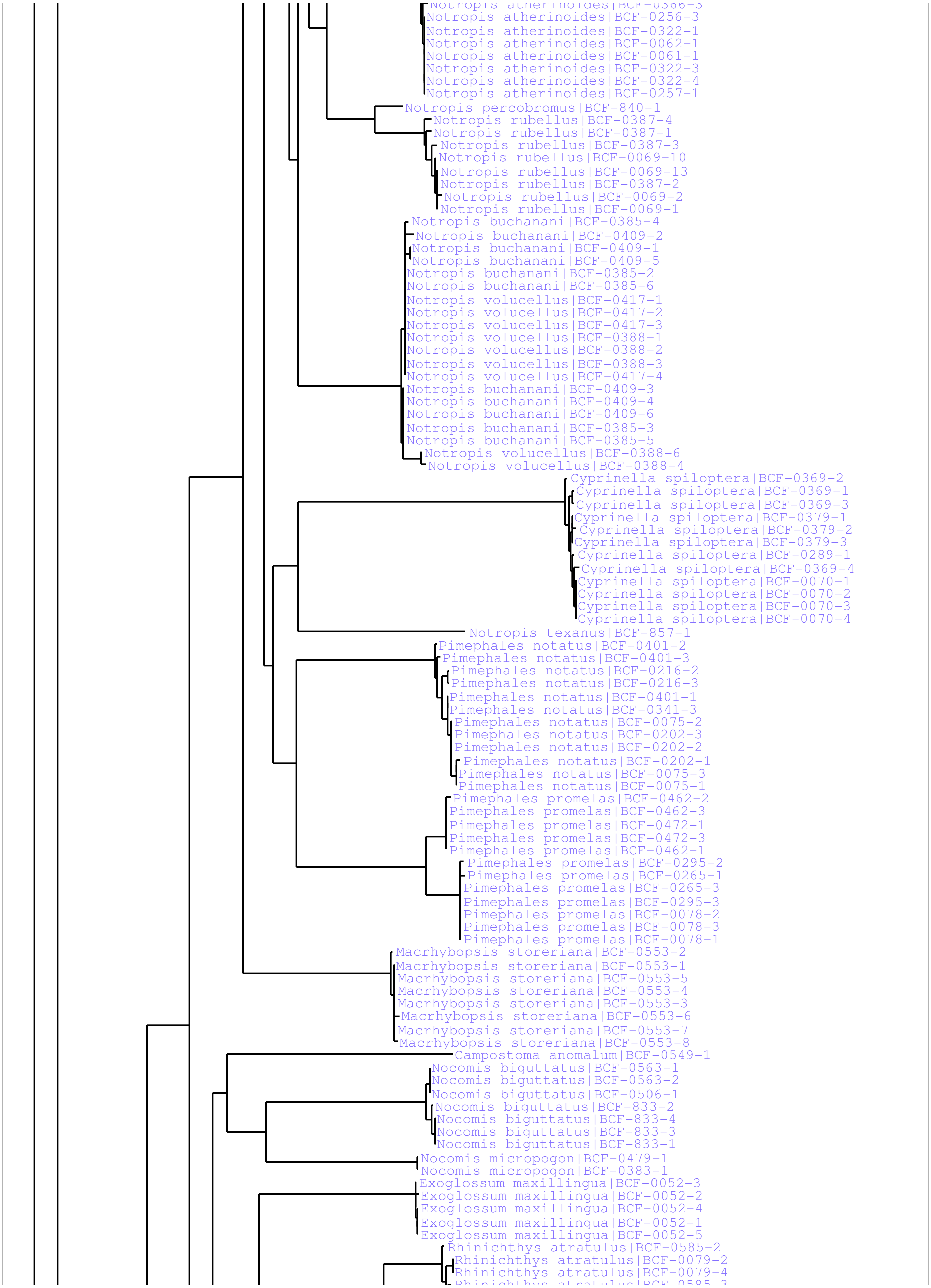
Notropis atherinoides|BCF-0256-3
Notropis atherinoides|BCF-0322-1
Notropis atherinoides|BCF-0062-1
Notropis atherinoides|BCF-0061-1
Notropis atherinoides|BCF-0322-3
Notropis atherinoides|BCF-0322-4
Notropis atherinoides|BCF-0257-1
Notropis percobromus|BCF-840-1
Notropis rubellus|BCF-0387-4
Notropis rubellus|BCF-0387-1
Notropis rubellus|BCF-0387-3
Notropis rubellus|BCF-0069-10
Notropis rubellus|BCF-0069-13
Notropis rubellus|BCF-0387-2
Notropis rubellus|BCF-0069-2
Notropis rubellus|BCF-0069-1
Notropis buchanani|BCF-0385-4
Notropis buchanani|BCF-0409-2
Notropis buchanani|BCF-0409-1
Notropis buchanani|BCF-0409-5
Notropis buchanani|BCF-0385-2
Notropis buchanani|BCF-0385-6
Notropis volucellus|BCF-0417-1
Notropis volucellus|BCF-0417-2
Notropis volucellus|BCF-0417-3
Notropis volucellus|BCF-0388-1
Notropis volucellus|BCF-0388-2
Notropis volucellus|BCF-0388-3
Notropis volucellus|BCF-0417-4
Notropis buchanani|BCF-0409-3
Notropis buchanani|BCF-0409-4
Notropis buchanani|BCF-0409-6
Notropis buchanani|BCF-0385-3
Notropis buchanani|BCF-0385-5
Notropis volucellus|BCF-0388-6
Notropis volucellus|BCF-0388-4
Cyprinella spiloptera|BCF-0369-2
Cyprinella spiloptera|BCF-0369-1
Cyprinella spiloptera|BCF-0369-3
Cyprinella spiloptera|BCF-0379-1
Cyprinella spiloptera|BCF-0379-2
Cyprinella spiloptera|BCF-0379-3
Cyprinella spiloptera|BCF-0289-1
Cyprinella spiloptera|BCF-0369-4
Cyprinella spiloptera|BCF-0070-1
Cyprinella spiloptera|BCF-0070-2
Cyprinella spiloptera|BCF-0070-3
Cyprinella spiloptera|BCF-0070-4
Notropis texanus|BCF-857-1
Pimephales notatus|BCF-0401-2
Pimephales notatus|BCF-0401-3
Pimephales notatus|BCF-0216-2
Pimephales notatus|BCF-0216-3
Pimephales notatus|BCF-0401-1
Pimephales notatus|BCF-0341-3
Pimephales notatus|BCF-0075-2
Pimephales notatus|BCF-0202-3
Pimephales notatus|BCF-0202-2
Pimephales notatus|BCF-0202-1
Pimephales notatus|BCF-0075-3
Pimephales notatus|BCF-0075-1
Pimephales promelas|BCF-0462-2
Pimephales promelas|BCF-0462-3
Pimephales promelas|BCF-0472-1
Pimephales promelas|BCF-0472-3
Pimephales promelas|BCF-0462-1
Pimephales promelas|BCF-0295-2
Pimephales promelas|BCF-0265-1
Pimephales promelas|BCF-0265-3
Pimephales promelas|BCF-0295-3
Pimephales promelas|BCF-0078-2
Pimephales promelas|BCF-0078-3
Pimephales promelas|BCF-0078-1
Macrhybopsis storeriana|BCF-0553-2
Macrhybopsis storeriana|BCF-0553-1
Macrhybopsis storeriana|BCF-0553-5
Macrhybopsis storeriana|BCF-0553-4
Macrhybopsis storeriana|BCF-0553-3
Macrhybopsis storeriana|BCF-0553-6
Macrhybopsis storeriana|BCF-0553-7
Macrhybopsis storeriana|BCF-0553-8
Campostoma anomalum|BCF-0549-1
Nocomis biguttatus|BCF-0563-1
Nocomis biguttatus|BCF-0563-2
Nocomis biguttatus|BCF-0506-1
Nocomis biguttatus|BCF-833-2
Nocomis biguttatus|BCF-833-4
Nocomis biguttatus|BCF-833-3
Nocomis biguttatus|BCF-833-1
Nocomis micropogon|BCF-0479-1
Nocomis micropogon|BCF-0383-1
Exoglossum maxillingua|BCF-0052-3
Exoglossum maxillingua|BCF-0052-2
Exoglossum maxillingua|BCF-0052-4
Exoglossum maxillingua|BCF-0052-1
Exoglossum maxillingua|BCF-0052-5
Rhinichthys atratulus|BCF-0585-2
Rhinichthys atratulus|BCF-0079-2
Rhinichthys atratulus|BCF-0079-4

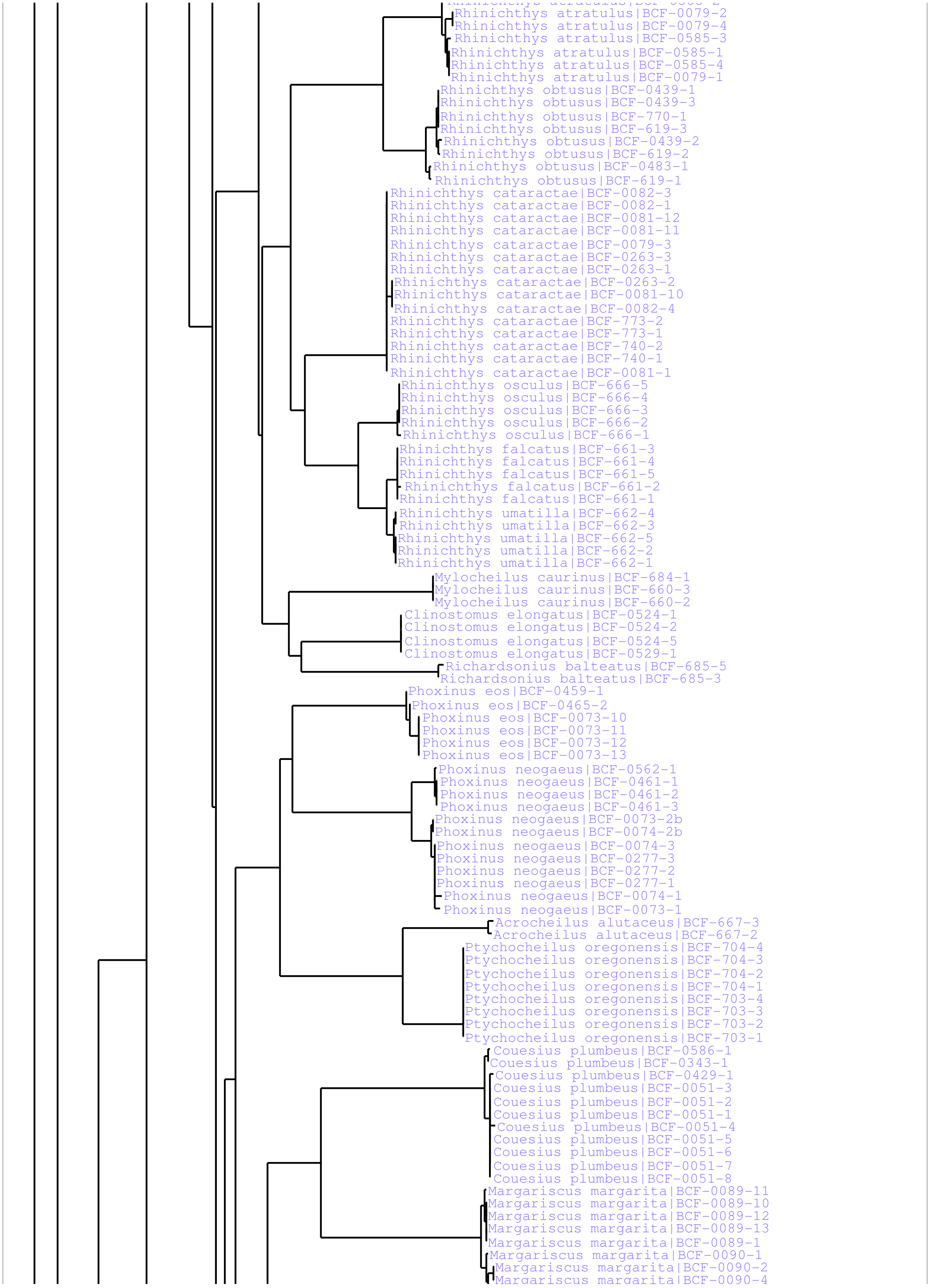
Rhinichthys atratulus|BCF-0079-2
Rhinichthys atratulus|BCF-0079-4
Rhinichthys atratulus|BCF-0585-3
Rhinichthys atratulus|BCF-0585-1
Rhinichthys atratulus|BCF-0585-4
Rhinichthys atratulus|BCF-0079-1
Rhinichthys obtusus|BCF-0439-1
Rhinichthys obtusus|BCF-0439-3
Rhinichthys obtusus|BCF-770-1
Rhinichthys obtusus|BCF-619-3
Rhinichthys obtusus|BCF-0439-2
Rhinichthys obtusus|BCF-619-2
Rhinichthys obtusus|BCF-0483-1
Rhinichthys obtusus|BCF-619-1
Rhinichthys cataractae|BCF-0082-3
Rhinichthys cataractae|BCF-0082-1
Rhinichthys cataractae|BCF-0081-12
Rhinichthys cataractae|BCF-0081-11
Rhinichthys cataractae|BCF-0079-3
Rhinichthys cataractae|BCF-0263-3
Rhinichthys cataractae|BCF-0263-1
Rhinichthys cataractae|BCF-0263-2
Rhinichthys cataractae|BCF-0081-10
Rhinichthys cataractae|BCF-0082-4
Rhinichthys cataractae|BCF-773-2
Rhinichthys cataractae|BCF-773-1
Rhinichthys cataractae|BCF-740-2
Rhinichthys cataractae|BCF-740-1
Rhinichthys cataractae|BCF-0081-1
Rhinichthys osculus|BCF-666-5
Rhinichthys osculus|BCF-666-4
Rhinichthys osculus|BCF-666-3
Rhinichthys osculus|BCF-666-2
Rhinichthys osculus|BCF-666-1
Rhinichthys falcatus|BCF-661-3
Rhinichthys falcatus|BCF-661-4
Rhinichthys falcatus|BCF-661-5
Rhinichthys falcatus|BCF-661-2
Rhinichthys falcatus|BCF-661-1
Rhinichthys umatilla|BCF-662-4
Rhinichthys umatilla|BCF-662-3
Rhinichthys umatilla|BCF-662-5
Rhinichthys umatilla|BCF-662-2
Rhinichthys umatilla|BCF-662-1
Mylocheilus caurinus|BCF-684-1
Mylocheilus caurinus|BCF-660-3
Mylocheilus caurinus|BCF-660-2
Clinostomus elongatus|BCF-0524-1
Clinostomus elongatus|BCF-0524-2
Clinostomus elongatus|BCF-0524-5
Clinostomus elongatus|BCF-0529-1
Richardsonius balteatus|BCF-685-5
Richardsonius balteatus|BCF-685-3
Phoxinus eos|BCF-0459-1
Phoxinus eos|BCF-0465-2
Phoxinus eos|BCF-0073-10
Phoxinus eos|BCF-0073-11
Phoxinus eos|BCF-0073-12
Phoxinus eos|BCF-0073-13
Phoxinus neogaeus|BCF-0562-1
Phoxinus neogaeus|BCF-0461-1
Phoxinus neogaeus|BCF-0461-2
Phoxinus neogaeus|BCF-0461-3
Phoxinus neogaeus|BCF-0073-2b
Phoxinus neogaeus|BCF-0074-2b
Phoxinus neogaeus|BCF-0074-3
Phoxinus neogaeus|BCF-0277-3
Phoxinus neogaeus|BCF-0277-2
Phoxinus neogaeus|BCF-0277-1
Phoxinus neogaeus|BCF-0074-1
Phoxinus neogaeus|BCF-0073-1
Acrocheilus alutaceus|BCF-667-3
Acrocheilus alutaceus|BCF-667-2
Ptychocheilus oregonensis|BCF-704-4
Ptychocheilus oregonensis|BCF-704-3
Ptychocheilus oregonensis|BCF-704-2
Ptychocheilus oregonensis|BCF-704-1
Ptychocheilus oregonensis|BCF-703-4
Ptychocheilus oregonensis|BCF-703-3
Ptychocheilus oregonensis|BCF-703-2
Ptychocheilus oregonensis|BCF-703-1
Couesius plumbeus|BCF-0586-1
Couesius plumbeus|BCF-0343-1
Couesius plumbeus|BCF-0429-1
Couesius plumbeus|BCF-0051-3
Couesius plumbeus|BCF-0051-2
Couesius plumbeus|BCF-0051-1
Couesius plumbeus|BCF-0051-4
Couesius plumbeus|BCF-0051-5
Couesius plumbeus|BCF-0051-6
Couesius plumbeus|BCF-0051-7
Couesius plumbeus|BCF-0051-8
Margariscus margarita|BCF-0089-11
Margariscus margarita|BCF-0089-10
Margariscus margarita|BCF-0089-12
Margariscus margarita|BCF-0089-13
Margariscus margarita|BCF-0089-1
Margariscus margarita|BCF-0090-1
Margariscus margarita|BCF-0090-2
Margariscus margarita|BCF-0090-4

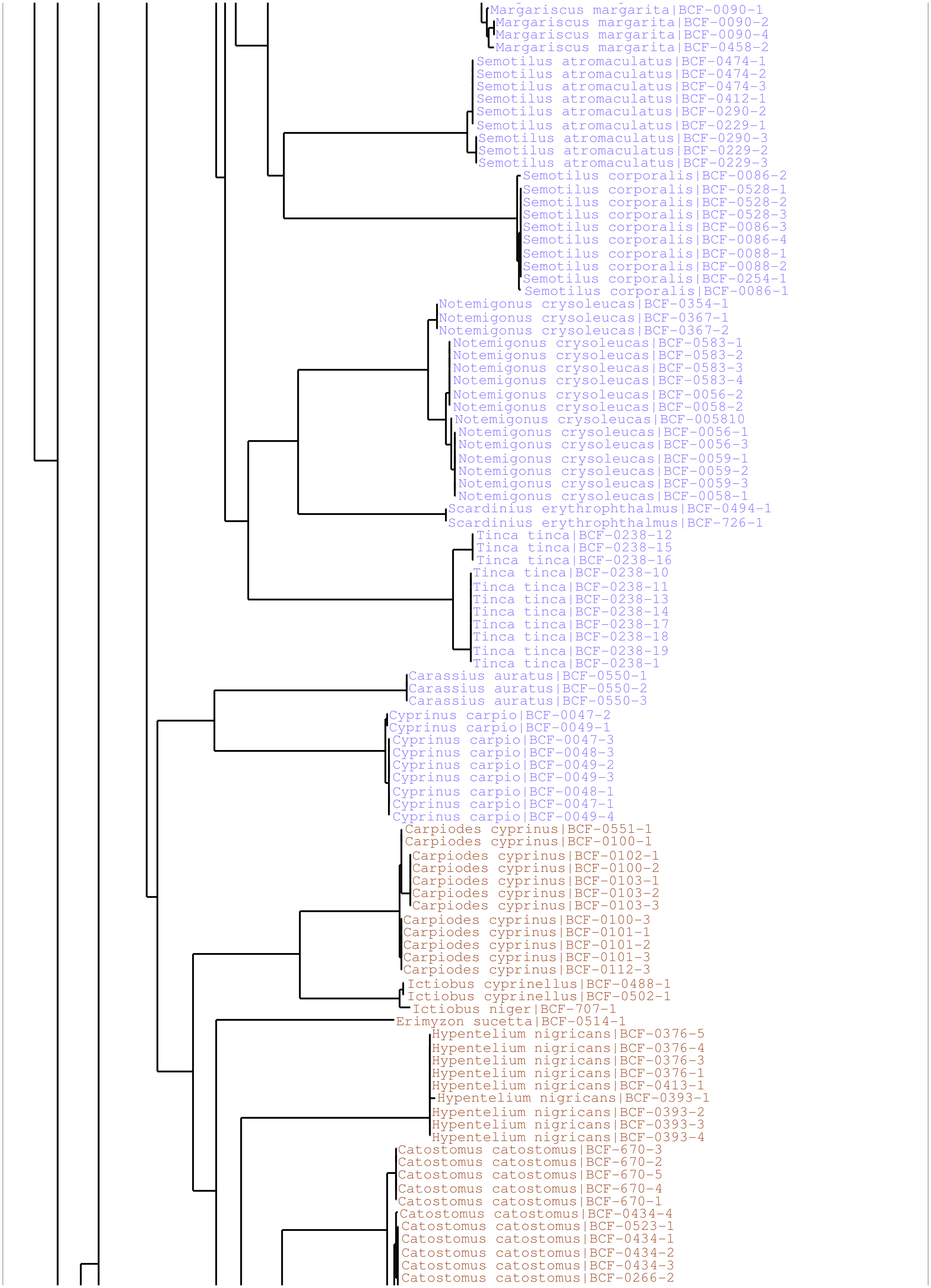
Margariscus margarita|BCF-0090-1
Margariscus margarita|BCF-0090-2
Margariscus margarita|BCF-0090-4
Margariscus margarita|BCF-0458-2
Semotilus atromaculatus|BCF-0474-1
Semotilus atromaculatus|BCF-0474-2
Semotilus atromaculatus|BCF-0474-3
Semotilus atromaculatus|BCF-0412-1
Semotilus atromaculatus|BCF-0290-2
Semotilus atromaculatus|BCF-0229-1
Semotilus atromaculatus|BCF-0290-3
Semotilus atromaculatus|BCF-0229-2
Semotilus atromaculatus|BCF-0229-3
Semotilus corporalis|BCF-0086-2
Semotilus corporalis|BCF-0528-1
Semotilus corporalis|BCF-0528-2
Semotilus corporalis|BCF-0528-3
Semotilus corporalis|BCF-0086-3
Semotilus corporalis|BCF-0086-4
Semotilus corporalis|BCF-0088-1
Semotilus corporalis|BCF-0088-2
Semotilus corporalis|BCF-0254-1
Semotilus corporalis|BCF-0086-1
Notemigonus crysoleucas|BCF-0354-1
Notemigonus crysoleucas|BCF-0367-1
Notemigonus crysoleucas|BCF-0367-2
Notemigonus crysoleucas|BCF-0583-1
Notemigonus crysoleucas|BCF-0583-2
Notemigonus crysoleucas|BCF-0583-3
Notemigonus crysoleucas|BCF-0583-4
Notemigonus crysoleucas|BCF-0056-2
Notemigonus crysoleucas|BCF-0058-2
Notemigonus crysoleucas|BCF-005810
Notemigonus crysoleucas|BCF-0056-1
Notemigonus crysoleucas|BCF-0056-3
Notemigonus crysoleucas|BCF-0059-1
Notemigonus crysoleucas|BCF-0059-2
Notemigonus crysoleucas|BCF-0059-3
Notemigonus crysoleucas|BCF-0058-1
Scardinius erythrophthalmus|BCF-0494-1
Scardinius erythrophthalmus|BCF-726-1
Tinca tinca|BCF-0238-12
Tinca tinca|BCF-0238-15
Tinca tinca|BCF-0238-16
Tinca tinca|BCF-0238-10
Tinca tinca|BCF-0238-11
Tinca tinca|BCF-0238-13
Tinca tinca|BCF-0238-14
Tinca tinca|BCF-0238-17
Tinca tinca|BCF-0238-18
Tinca tinca|BCF-0238-19
Tinca tinca|BCF-0238-1
Carassius auratus|BCF-0550-1
Carassius auratus|BCF-0550-2
Carassius auratus|BCF-0550-3
Cyprinus carpio|BCF-0047-2
Cyprinus carpio|BCF-0049-1
Cyprinus carpio|BCF-0047-3
Cyprinus carpio|BCF-0048-3
Cyprinus carpio|BCF-0049-2
Cyprinus carpio|BCF-0049-3
Cyprinus carpio|BCF-0048-1
Cyprinus carpio|BCF-0047-1
Cyprinus carpio|BCF-0049-4
Carpiodes cyprinus|BCF-0551-1
Carpiodes cyprinus|BCF-0100-1
Carpiodes cyprinus|BCF-0102-1
Carpiodes cyprinus|BCF-0100-2
Carpiodes cyprinus|BCF-0103-1
Carpiodes cyprinus|BCF-0103-2
Carpiodes cyprinus|BCF-0103-3
Carpiodes cyprinus|BCF-0100-3
Carpiodes cyprinus|BCF-0101-1
Carpiodes cyprinus|BCF-0101-2
Carpiodes cyprinus|BCF-0101-3
Carpiodes cyprinus|BCF-0112-3
Ictiobus cyprinellus|BCF-0488-1
Ictiobus cyprinellus|BCF-0502-1
Ictiobus niger|BCF-707-1
Erimyzon sucetta|BCF-0514-1
Hypentelium nigricans|BCF-0376-5
Hypentelium nigricans|BCF-0376-4
Hypentelium nigricans|BCF-0376-3
Hypentelium nigricans|BCF-0376-1
Hypentelium nigricans|BCF-0413-1
Hypentelium nigricans|BCF-0393-1
Hypentelium nigricans|BCF-0393-2
Hypentelium nigricans|BCF-0393-3
Hypentelium nigricans|BCF-0393-4
Catostomus catostomus|BCF-670-3
Catostomus catostomus|BCF-670-2
Catostomus catostomus|BCF-670-5
Catostomus catostomus|BCF-670-4
Catostomus catostomus|BCF-670-1
Catostomus catostomus|BCF-0434-4
Catostomus catostomus|BCF-0523-1
Catostomus catostomus|BCF-0434-1
Catostomus catostomus|BCF-0434-2
Catostomus catostomus|BCF-0434-3
Catostomus catostomus|BCF-0266-2

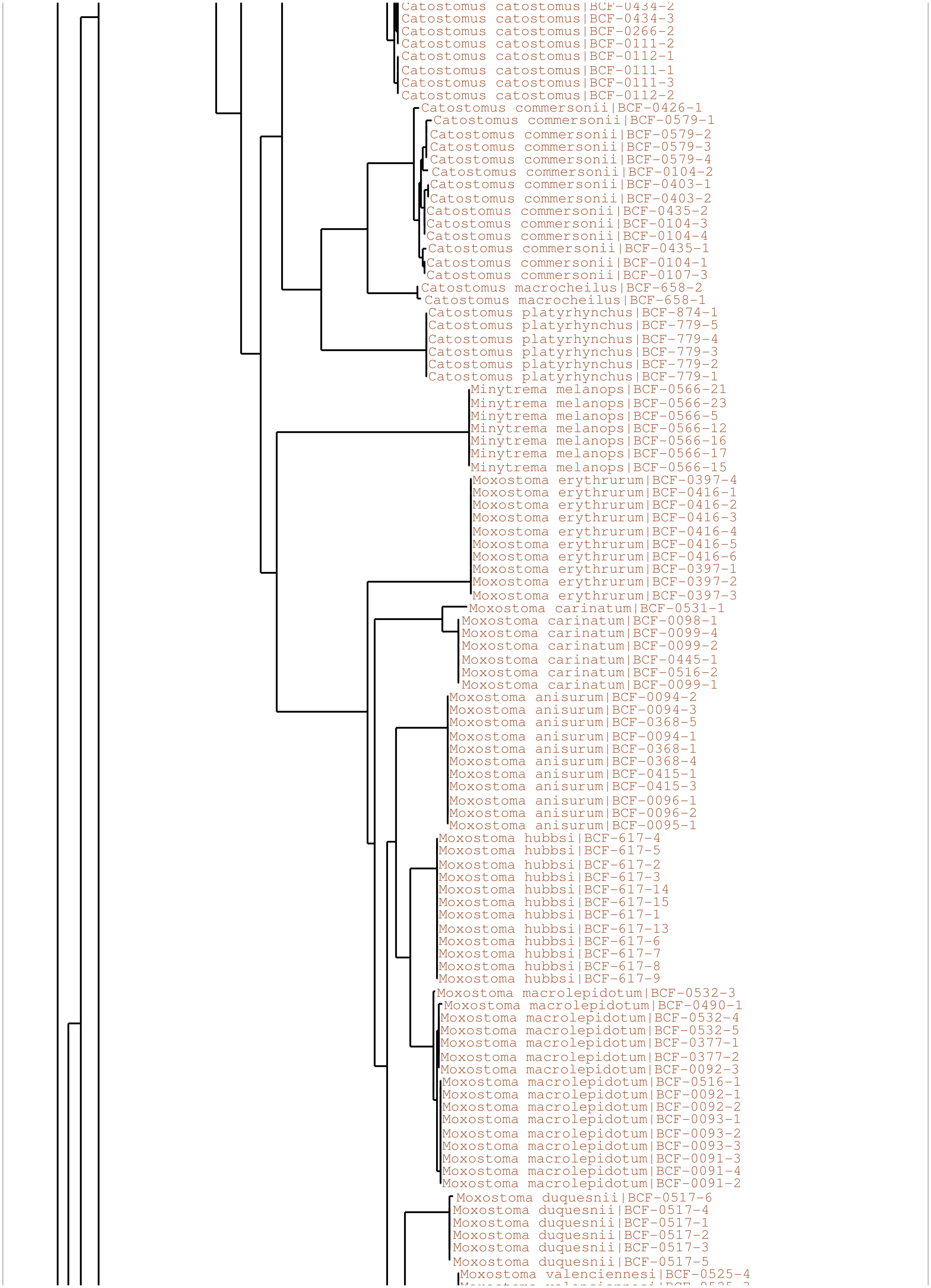
Catostomus catostomus|BCF-0434-2
Catostomus catostomus|BCF-0434-3
Catostomus catostomus|BCF-0266-2
Catostomus catostomus|BCF-0111-2
Catostomus catostomus|BCF-0112-1
Catostomus catostomus|BCF-0111-1
Catostomus catostomus|BCF-0111-3
Catostomus catostomus|BCF-0112-2
Catostomus commersonii|BCF-0426-1
Catostomus commersonii|BCF-0579-1
Catostomus commersonii|BCF-0579-2
Catostomus commersonii|BCF-0579-3
Catostomus commersonii|BCF-0579-4
Catostomus commersonii|BCF-0104-2
Catostomus commersonii|BCF-0403-1
Catostomus commersonii|BCF-0403-2
Catostomus commersonii|BCF-0435-2
Catostomus commersonii|BCF-0104-3
Catostomus commersonii|BCF-0104-4
Catostomus commersonii|BCF-0435-1
Catostomus commersonii|BCF-0104-1
Catostomus commersonii|BCF-0107-3
Catostomus macrocheilus|BCF-658-2
Catostomus macrocheilus|BCF-658-1
Catostomus platyrhynchus|BCF-874-1
Catostomus platyrhynchus|BCF-779-5
Catostomus platyrhynchus|BCF-779-4
Catostomus platyrhynchus|BCF-779-3
Catostomus platyrhynchus|BCF-779-2
Catostomus platyrhynchus|BCF-779-1
Minytrema melanops|BCF-0566-21
Minytrema melanops|BCF-0566-23
Minytrema melanops|BCF-0566-5
Minytrema melanops|BCF-0566-12
Minytrema melanops|BCF-0566-16
Minytrema melanops|BCF-0566-17
Minytrema melanops|BCF-0566-15
Moxostoma erythrurum|BCF-0397-4
Moxostoma erythrurum|BCF-0416-1
Moxostoma erythrurum|BCF-0416-2
Moxostoma erythrurum|BCF-0416-3
Moxostoma erythrurum|BCF-0416-4
Moxostoma erythrurum|BCF-0416-5
Moxostoma erythrurum|BCF-0416-6
Moxostoma erythrurum|BCF-0397-1
Moxostoma erythrurum|BCF-0397-2
Moxostoma erythrurum|BCF-0397-3
Moxostoma carinatum|BCF-0531-1
Moxostoma carinatum|BCF-0098-1
Moxostoma carinatum|BCF-0099-4
Moxostoma carinatum|BCF-0099-2
Moxostoma carinatum|BCF-0445-1
Moxostoma carinatum|BCF-0516-2
Moxostoma carinatum|BCF-0099-1
Moxostoma anisurum|BCF-0094-2
Moxostoma anisurum|BCF-0094-3
Moxostoma anisurum|BCF-0368-5
Moxostoma anisurum|BCF-0094-1
Moxostoma anisurum|BCF-0368-1
Moxostoma anisurum|BCF-0368-4
Moxostoma anisurum|BCF-0415-1
Moxostoma anisurum|BCF-0415-3
Moxostoma anisurum|BCF-0096-1
Moxostoma anisurum|BCF-0096-2
Moxostoma anisurum|BCF-0095-1
Moxostoma hubbsi|BCF-617-4
Moxostoma hubbsi|BCF-617-5
Moxostoma hubbsi|BCF-617-2
Moxostoma hubbsi|BCF-617-3
Moxostoma hubbsi|BCF-617-14
Moxostoma hubbsi|BCF-617-15
Moxostoma hubbsi|BCF-617-1
Moxostoma hubbsi|BCF-617-13
Moxostoma hubbsi|BCF-617-6
Moxostoma hubbsi|BCF-617-7
Moxostoma hubbsi|BCF-617-8
Moxostoma hubbsi|BCF-617-9
Moxostoma macrolepidotum|BCF-0532-3
Moxostoma macrolepidotum|BCF-0490-1
Moxostoma macrolepidotum|BCF-0532-4
Moxostoma macrolepidotum|BCF-0532-5
Moxostoma macrolepidotum|BCF-0377-1
Moxostoma macrolepidotum|BCF-0377-2
Moxostoma macrolepidotum|BCF-0092-3
Moxostoma macrolepidotum|BCF-0516-1
Moxostoma macrolepidotum|BCF-0092-1
Moxostoma macrolepidotum|BCF-0092-2
Moxostoma macrolepidotum|BCF-0093-1
Moxostoma macrolepidotum|BCF-0093-2
Moxostoma macrolepidotum|BCF-0093-3
Moxostoma macrolepidotum|BCF-0091-3
Moxostoma macrolepidotum|BCF-0091-4
Moxostoma macrolepidotum|BCF-0091-2
Moxostoma duquesnii|BCF-0517-6
Moxostoma duquesnii|BCF-0517-4
Moxostoma duquesnii|BCF-0517-1
Moxostoma duquesnii|BCF-0517-2
Moxostoma duquesnii|BCF-0517-3
Moxostoma duquesnii|BCF-0517-5
Moxostoma valenciennesi|BCF-0525-4

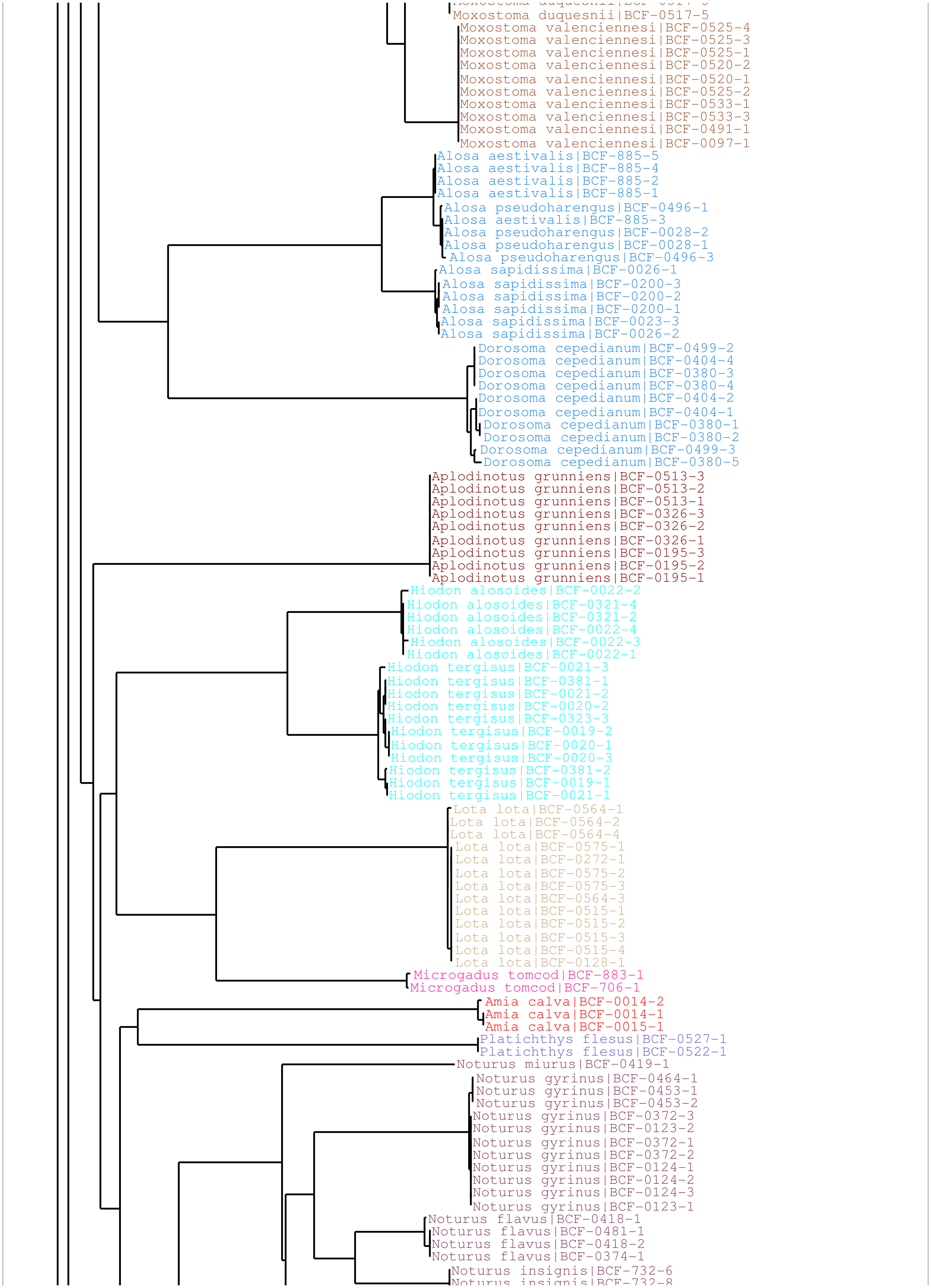
Moxostoma duquesnii|BCF-0517-5
Moxostoma valenciennesi|BCF-0525-4
Moxostoma valenciennesi|BCF-0525-3
Moxostoma valenciennesi|BCF-0525-1
Moxostoma valenciennesi|BCF-0520-2
Moxostoma valenciennesi|BCF-0520-1
Moxostoma valenciennesi|BCF-0525-2
Moxostoma valenciennesi|BCF-0533-1
Moxostoma valenciennesi|BCF-0533-3
Moxostoma valenciennesi|BCF-0491-1
Moxostoma valenciennesi|BCF-0097-1
Alosa aestivalis|BCF-885-5
Alosa aestivalis|BCF-885-4
Alosa aestivalis|BCF-885-2
Alosa aestivalis|BCF-885-1
Alosa pseudoharengus|BCF-0496-1
Alosa aestivalis|BCF-885-3
Alosa pseudoharengus|BCF-0028-2
Alosa pseudoharengus|BCF-0028-1
Alosa pseudoharengus|BCF-0496-3
Alosa sapidissima|BCF-0026-1
Alosa sapidissima|BCF-0200-3
Alosa sapidissima|BCF-0200-2
Alosa sapidissima|BCF-0200-1
Alosa sapidissima|BCF-0023-3
Alosa sapidissima|BCF-0026-2
Dorosoma cepedianum|BCF-0499-2
Dorosoma cepedianum|BCF-0404-4
Dorosoma cepedianum|BCF-0380-3
Dorosoma cepedianum|BCF-0380-4
Dorosoma cepedianum|BCF-0404-2
Dorosoma cepedianum|BCF-0404-1
Dorosoma cepedianum|BCF-0380-1
Dorosoma cepedianum|BCF-0380-2
Dorosoma cepedianum|BCF-0499-3
Dorosoma cepedianum|BCF-0380-5
Aplodinotus grunniens|BCF-0513-3
Aplodinotus grunniens|BCF-0513-2
Aplodinotus grunniens|BCF-0513-1
Aplodinotus grunniens|BCF-0326-3
Aplodinotus grunniens|BCF-0326-2
Aplodinotus grunniens|BCF-0326-1
Aplodinotus grunniens|BCF-0195-3
Aplodinotus grunniens|BCF-0195-2
Aplodinotus grunniens|BCF-0195-1
Hiodon alosoides|BCF-0022-2
Hiodon alosoides|BCF-0321-4
Hiodon alosoides|BCF-0321-2
Hiodon alosoides|BCF-0022-4
Hiodon alosoides|BCF-0022-3
Hiodon alosoides|BCF-0022-1
Hiodon tergisus|BCF-0021-3
Hiodon tergisus|BCF-0381-1
Hiodon tergisus|BCF-0021-2
Hiodon tergisus|BCF-0020-2
Hiodon tergisus|BCF-0323-3
Hiodon tergisus|BCF-0019-2
Hiodon tergisus|BCF-0020-1
Hiodon tergisus|BCF-0020-3
Hiodon tergisus|BCF-0381-2
Hiodon tergisus|BCF-0019-1
Hiodon tergisus|BCF-0021-1
Lota lota|BCF-0564-1
Lota lota|BCF-0564-2
Lota lota|BCF-0564-4
Lota lota|BCF-0575-1
Lota lota|BCF-0272-1
Lota lota|BCF-0575-2
Lota lota|BCF-0575-3
Lota lota|BCF-0564-3
Lota lota|BCF-0515-1
Lota lota|BCF-0515-2
Lota lota|BCF-0515-3
Lota lota|BCF-0515-4
Lota lota|BCF-0128-1
Microgadus tomcod|BCF-883-1
Microgadus tomcod|BCF-706-1
Amia calva|BCF-0014-2
Amia calva|BCF-0014-1
Amia calva|BCF-0015-1
Platichthys flesus|BCF-0527-1
Platichthys flesus|BCF-0522-1
Noturus miurus|BCF-0419-1
Noturus gyrinus|BCF-0464-1
Noturus gyrinus|BCF-0453-1
Noturus gyrinus|BCF-0453-2
Noturus gyrinus|BCF-0372-3
Noturus gyrinus|BCF-0123-2
Noturus gyrinus|BCF-0372-1
Noturus gyrinus|BCF-0372-2
Noturus gyrinus|BCF-0124-1
Noturus gyrinus|BCF-0124-2
Noturus gyrinus|BCF-0124-3
Noturus gyrinus|BCF-0123-1
Noturus flavus|BCF-0418-1
Noturus flavus|BCF-0481-1
Noturus flavus|BCF-0418-2
Noturus flavus|BCF-0374-1
Noturus insignis|BCF-732-6

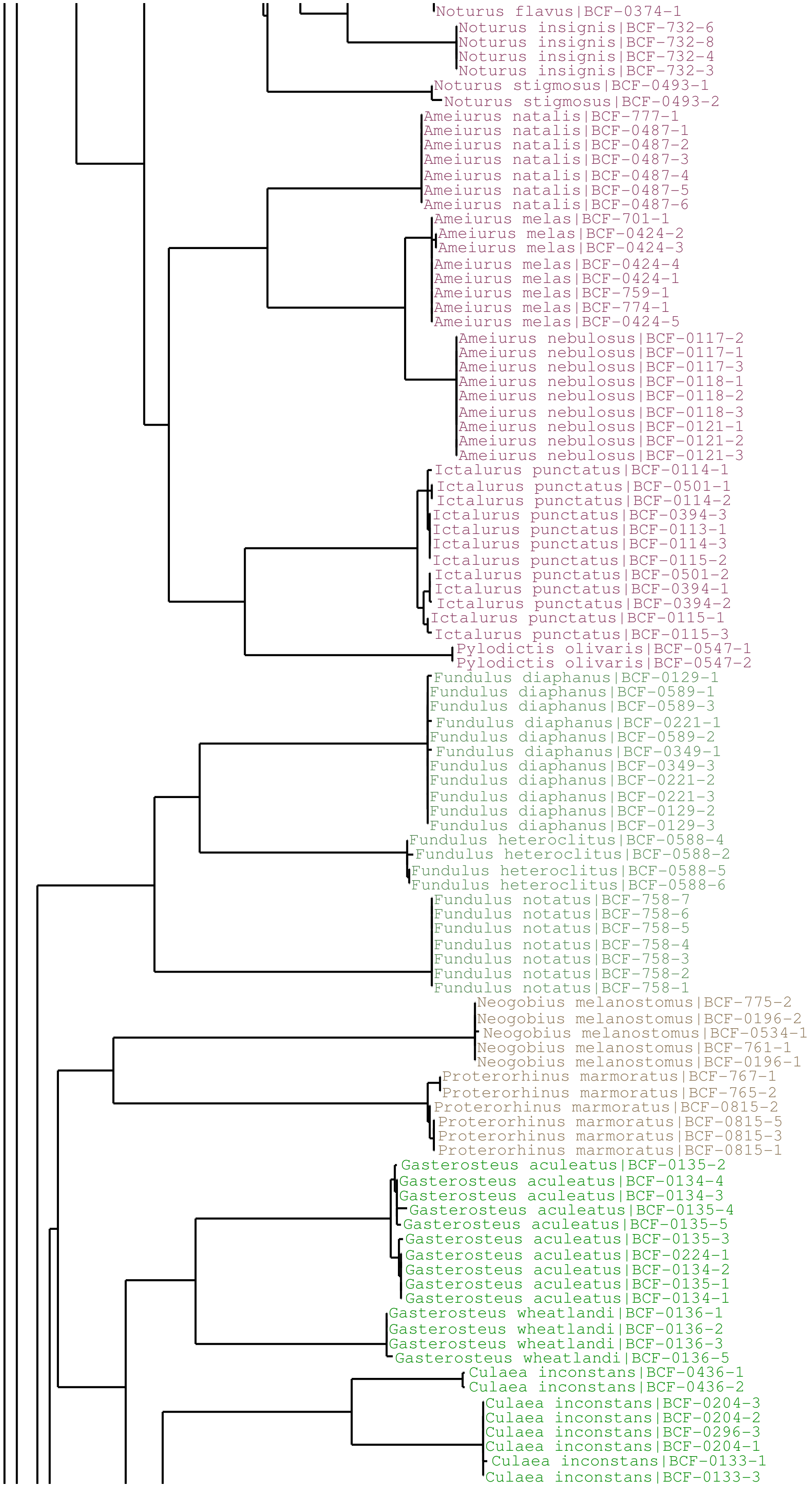
Noturus flavus|BCF-0374-1
Noturus insignis|BCF-732-6
Noturus insignis|BCF-732-8
Noturus insignis|BCF-732-4
Noturus insignis|BCF-732-3
Noturus stigmosus|BCF-0493-1
Noturus stigmosus|BCF-0493-2
Ameiurus natalis|BCF-777-1
Ameiurus natalis|BCF-0487-1
Ameiurus natalis|BCF-0487-2
Ameiurus natalis|BCF-0487-3
Ameiurus natalis|BCF-0487-4
Ameiurus natalis|BCF-0487-5
Ameiurus natalis|BCF-0487-6
Ameiurus melas|BCF-701-1
Ameiurus melas|BCF-0424-2
Ameiurus melas|BCF-0424-3
Ameiurus melas|BCF-0424-4
Ameiurus melas|BCF-0424-1
Ameiurus melas|BCF-759-1
Ameiurus melas|BCF-774-1
Ameiurus melas|BCF-0424-5
Ameiurus nebulosus|BCF-0117-2
Ameiurus nebulosus|BCF-0117-1
Ameiurus nebulosus|BCF-0117-3
Ameiurus nebulosus|BCF-0118-1
Ameiurus nebulosus|BCF-0118-2
Ameiurus nebulosus|BCF-0118-3
Ameiurus nebulosus|BCF-0121-1
Ameiurus nebulosus|BCF-0121-2
Ameiurus nebulosus|BCF-0121-3
Ictalurus punctatus|BCF-0114-1
Ictalurus punctatus|BCF-0501-1
Ictalurus punctatus|BCF-0114-2
Ictalurus punctatus|BCF-0394-3
Ictalurus punctatus|BCF-0113-1
Ictalurus punctatus|BCF-0114-3
Ictalurus punctatus|BCF-0115-2
Ictalurus punctatus|BCF-0501-2
Ictalurus punctatus|BCF-0394-1
Ictalurus punctatus|BCF-0394-2
Ictalurus punctatus|BCF-0115-1
Ictalurus punctatus|BCF-0115-3
Pylodictis olivaris|BCF-0547-1
Pylodictis olivaris|BCF-0547-2
Fundulus diaphanus|BCF-0129-1
Fundulus diaphanus|BCF-0589-1
Fundulus diaphanus|BCF-0589-3
Fundulus diaphanus|BCF-0221-1
Fundulus diaphanus|BCF-0589-2
Fundulus diaphanus|BCF-0349-1
Fundulus diaphanus|BCF-0349-3
Fundulus diaphanus|BCF-0221-2
Fundulus diaphanus|BCF-0221-3
Fundulus diaphanus|BCF-0129-2
Fundulus diaphanus|BCF-0129-3
Fundulus heteroclitus|BCF-0588-4
Fundulus heteroclitus|BCF-0588-2
Fundulus heteroclitus|BCF-0588-5
Fundulus heteroclitus|BCF-0588-6
Fundulus notatus|BCF-758-7
Fundulus notatus|BCF-758-6
Fundulus notatus|BCF-758-5
Fundulus notatus|BCF-758-4
Fundulus notatus|BCF-758-3
Fundulus notatus|BCF-758-2
Fundulus notatus|BCF-758-1
Neogobius melanostomus|BCF-775-2
Neogobius melanostomus|BCF-0196-2
Neogobius melanostomus|BCF-0534-1
Neogobius melanostomus|BCF-761-1
Neogobius melanostomus|BCF-0196-1
Proterorhinus marmoratus|BCF-767-1
Proterorhinus marmoratus|BCF-765-2
Proterorhinus marmoratus|BCF-0815-2
Proterorhinus marmoratus|BCF-0815-5
Proterorhinus marmoratus|BCF-0815-3
Proterorhinus marmoratus|BCF-0815-1
Gasterosteus aculeatus|BCF-0135-2
Gasterosteus aculeatus|BCF-0134-4
Gasterosteus aculeatus|BCF-0134-3
Gasterosteus aculeatus|BCF-0135-4
Gasterosteus aculeatus|BCF-0135-5
Gasterosteus aculeatus|BCF-0135-3
Gasterosteus aculeatus|BCF-0224-1
Gasterosteus aculeatus|BCF-0134-2
Gasterosteus aculeatus|BCF-0135-1
Gasterosteus aculeatus|BCF-0134-1
Gasterosteus wheatlandi|BCF-0136-1
Gasterosteus wheatlandi|BCF-0136-2
Gasterosteus wheatlandi|BCF-0136-3
Gasterosteus wheatlandi|BCF-0136-5
Culaea inconstans|BCF-0436-1
Culaea inconstans|BCF-0436-2
Culaea inconstans|BCF-0204-3
Culaea inconstans|BCF-0204-2
Culaea inconstans|BCF-0296-3
Culaea inconstans|BCF-0204-1
Culaea inconstans|BCF-0133-1
Culaea inconstans|BCF-0133-3

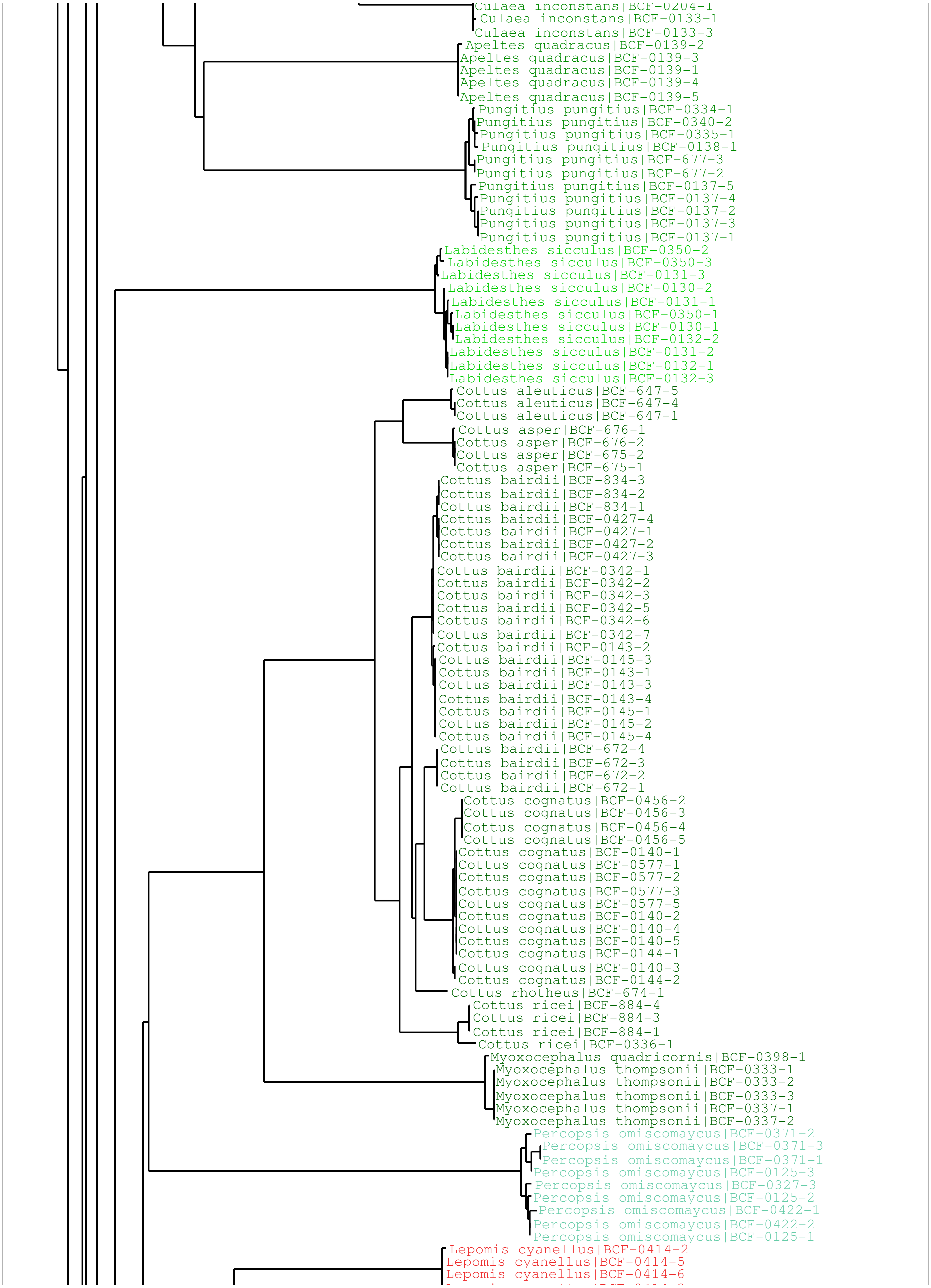
Culaea inconstans|BCF-0204-1
Culaea inconstans|BCF-0133-1
Culaea inconstans|BCF-0133-3
Apeltes quadracus|BCF-0139-2
Apeltes quadracus|BCF-0139-3
Apeltes quadracus|BCF-0139-1
Apeltes quadracus|BCF-0139-4
Apeltes quadracus|BCF-0139-5
Pungitius pungitius|BCF-0334-1
Pungitius pungitius|BCF-0340-2
Pungitius pungitius|BCF-0335-1
Pungitius pungitius|BCF-0138-1
Pungitius pungitius|BCF-677-3
Pungitius pungitius|BCF-677-2
Pungitius pungitius|BCF-0137-5
Pungitius pungitius|BCF-0137-4
Pungitius pungitius|BCF-0137-2
Pungitius pungitius|BCF-0137-3
Pungitius pungitius|BCF-0137-1
Labidesthes sicculus|BCF-0350-2
Labidesthes sicculus|BCF-0350-3
Labidesthes sicculus|BCF-0131-3
Labidesthes sicculus|BCF-0130-2
Labidesthes sicculus|BCF-0131-1
Labidesthes sicculus|BCF-0350-1
Labidesthes sicculus|BCF-0130-1
Labidesthes sicculus|BCF-0132-2
Labidesthes sicculus|BCF-0131-2
Labidesthes sicculus|BCF-0132-1
Labidesthes sicculus|BCF-0132-3
Cottus aleuticus|BCF-647-5
Cottus aleuticus|BCF-647-4
Cottus aleuticus|BCF-647-1
Cottus asper|BCF-676-1
Cottus asper|BCF-676-2
Cottus asper|BCF-675-2
Cottus asper|BCF-675-1
Cottus bairdii|BCF-834-3
Cottus bairdii|BCF-834-2
Cottus bairdii|BCF-834-1
Cottus bairdii|BCF-0427-4
Cottus bairdii|BCF-0427-1
Cottus bairdii|BCF-0427-2
Cottus bairdii|BCF-0427-3
Cottus bairdii|BCF-0342-1
Cottus bairdii|BCF-0342-2
Cottus bairdii|BCF-0342-3
Cottus bairdii|BCF-0342-5
Cottus bairdii|BCF-0342-6
Cottus bairdii|BCF-0342-7
Cottus bairdii|BCF-0143-2
Cottus bairdii|BCF-0145-3
Cottus bairdii|BCF-0143-1
Cottus bairdii|BCF-0143-3
Cottus bairdii|BCF-0143-4
Cottus bairdii|BCF-0145-1
Cottus bairdii|BCF-0145-2
Cottus bairdii|BCF-0145-4
Cottus bairdii|BCF-672-4
Cottus bairdii|BCF-672-3
Cottus bairdii|BCF-672-2
Cottus bairdii|BCF-672-1
Cottus cognatus|BCF-0456-2
Cottus cognatus|BCF-0456-3
Cottus cognatus|BCF-0456-4
Cottus cognatus|BCF-0456-5
Cottus cognatus|BCF-0140-1
Cottus cognatus|BCF-0577-1
Cottus cognatus|BCF-0577-2
Cottus cognatus|BCF-0577-3
Cottus cognatus|BCF-0577-5
Cottus cognatus|BCF-0140-2
Cottus cognatus|BCF-0140-4
Cottus cognatus|BCF-0140-5
Cottus cognatus|BCF-0144-1
Cottus cognatus|BCF-0140-3
Cottus cognatus|BCF-0144-2
Cottus rhotheus|BCF-674-1
Cottus ricei|BCF-884-4
Cottus ricei|BCF-884-3
Cottus ricei|BCF-884-1
Cottus ricei|BCF-0336-1
Myoxocephalus quadricornis|BCF-0398-1
Myoxocephalus thompsonii|BCF-0333-1
Myoxocephalus thompsonii|BCF-0333-2
Myoxocephalus thompsonii|BCF-0333-3
Myoxocephalus thompsonii|BCF-0337-1
Myoxocephalus thompsonii|BCF-0337-2
Percopsis omiscomaycus|BCF-0371-2
Percopsis omiscomaycus|BCF-0371-3
Percopsis omiscomaycus|BCF-0371-1
Percopsis omiscomaycus|BCF-0125-3
Percopsis omiscomaycus|BCF-0327-3
Percopsis omiscomaycus|BCF-0125-2
Percopsis omiscomaycus|BCF-0422-1
Percopsis omiscomaycus|BCF-0422-2
Percopsis omiscomaycus|BCF-0125-1
Lepomis cyanellus|BCF-0414-2
Lepomis cyanellus|BCF-0414-5
Lepomis cyanellus|BCF-0414-6

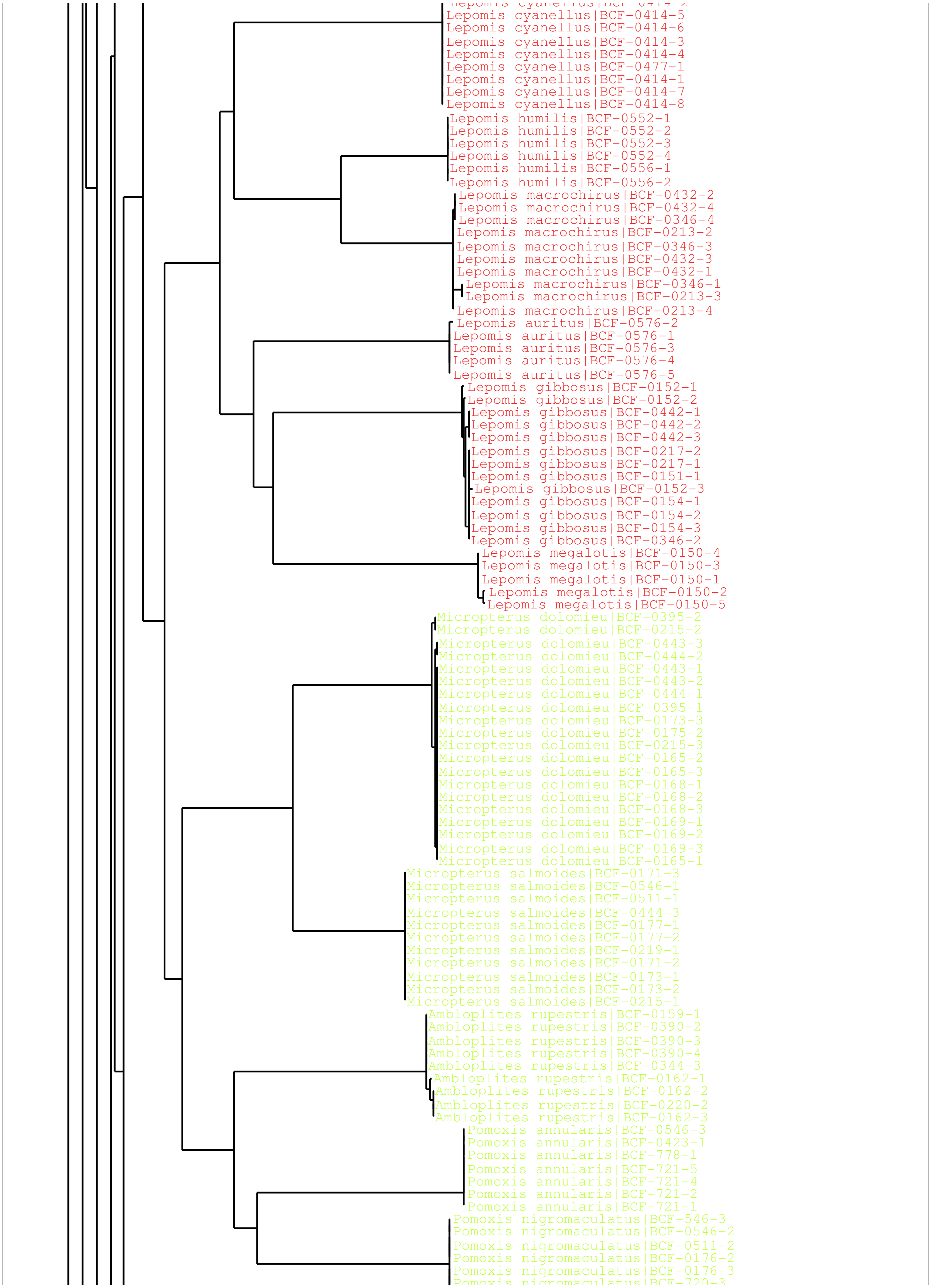
Lepomis cyanellus|BCF-0414-5
Lepomis cyanellus|BCF-0414-6
Lepomis cyanellus|BCF-0414-3
Lepomis cyanellus|BCF-0414-4
Lepomis cyanellus|BCF-0477-1
Lepomis cyanellus|BCF-0414-1
Lepomis cyanellus|BCF-0414-7
Lepomis cyanellus|BCF-0414-8
Lepomis humilis|BCF-0552-1
Lepomis humilis|BCF-0552-2
Lepomis humilis|BCF-0552-3
Lepomis humilis|BCF-0552-4
Lepomis humilis|BCF-0556-1
Lepomis humilis|BCF-0556-2
Lepomis macrochirus|BCF-0432-2
Lepomis macrochirus|BCF-0432-4
Lepomis macrochirus|BCF-0346-4
Lepomis macrochirus|BCF-0213-2
Lepomis macrochirus|BCF-0346-3
Lepomis macrochirus|BCF-0432-3
Lepomis macrochirus|BCF-0432-1
Lepomis macrochirus|BCF-0346-1
Lepomis macrochirus|BCF-0213-3
Lepomis macrochirus|BCF-0213-4
Lepomis auritus|BCF-0576-2
Lepomis auritus|BCF-0576-1
Lepomis auritus|BCF-0576-3
Lepomis auritus|BCF-0576-4
Lepomis auritus|BCF-0576-5
Lepomis gibbosus|BCF-0152-1
Lepomis gibbosus|BCF-0152-2
Lepomis gibbosus|BCF-0442-1
Lepomis gibbosus|BCF-0442-2
Lepomis gibbosus|BCF-0442-3
Lepomis gibbosus|BCF-0217-2
Lepomis gibbosus|BCF-0217-1
Lepomis gibbosus|BCF-0151-1
Lepomis gibbosus|BCF-0152-3
Lepomis gibbosus|BCF-0154-1
Lepomis gibbosus|BCF-0154-2
Lepomis gibbosus|BCF-0154-3
Lepomis gibbosus|BCF-0346-2
Lepomis megalotis|BCF-0150-4
Lepomis megalotis|BCF-0150-3
Lepomis megalotis|BCF-0150-1
Lepomis megalotis|BCF-0150-2
Lepomis megalotis|BCF-0150-5
Micropterus dolomieu|BCF-0395-2
Micropterus dolomieu|BCF-0215-2
Micropterus dolomieu|BCF-0443-3
Micropterus dolomieu|BCF-0444-2
Micropterus dolomieu|BCF-0443-1
Micropterus dolomieu|BCF-0443-2
Micropterus dolomieu|BCF-0444-1
Micropterus dolomieu|BCF-0395-1
Micropterus dolomieu|BCF-0173-3
Micropterus dolomieu|BCF-0175-2
Micropterus dolomieu|BCF-0215-3
Micropterus dolomieu|BCF-0165-2
Micropterus dolomieu|BCF-0165-3
Micropterus dolomieu|BCF-0168-1
Micropterus dolomieu|BCF-0168-2
Micropterus dolomieu|BCF-0168-3
Micropterus dolomieu|BCF-0169-1
Micropterus dolomieu|BCF-0169-2
Micropterus dolomieu|BCF-0169-3
Micropterus dolomieu|BCF-0165-1
Micropterus salmoides|BCF-0171-3
Micropterus salmoides|BCF-0546-1
Micropterus salmoides|BCF-0511-1
Micropterus salmoides|BCF-0444-3
Micropterus salmoides|BCF-0177-1
Micropterus salmoides|BCF-0177-2
Micropterus salmoides|BCF-0219-1
Micropterus salmoides|BCF-0171-2
Micropterus salmoides|BCF-0173-1
Micropterus salmoides|BCF-0173-2
Micropterus salmoides|BCF-0215-1
Ambloplites rupestris|BCF-0159-1
Ambloplites rupestris|BCF-0390-2
Ambloplites rupestris|BCF-0390-3
Ambloplites rupestris|BCF-0390-4
Ambloplites rupestris|BCF-0344-3
Ambloplites rupestris|BCF-0162-1
Ambloplites rupestris|BCF-0162-2
Ambloplites rupestris|BCF-0220-2
Ambloplites rupestris|BCF-0162-3
Pomoxis annularis|BCF-0546-3
Pomoxis annularis|BCF-0423-1
Pomoxis annularis|BCF-778-1
Pomoxis annularis|BCF-721-5
Pomoxis annularis|BCF-721-4
Pomoxis annularis|BCF-721-2
Pomoxis annularis|BCF-721-1
Pomoxis nigromaculatus|BCF-546-3
Pomoxis nigromaculatus|BCF-0546-2
Pomoxis nigromaculatus|BCF-0511-2
Pomoxis nigromaculatus|BCF-0176-2
Pomoxis nigromaculatus|BCF-0176-3
Pomoxis nigromaculatus|BCF-720-3

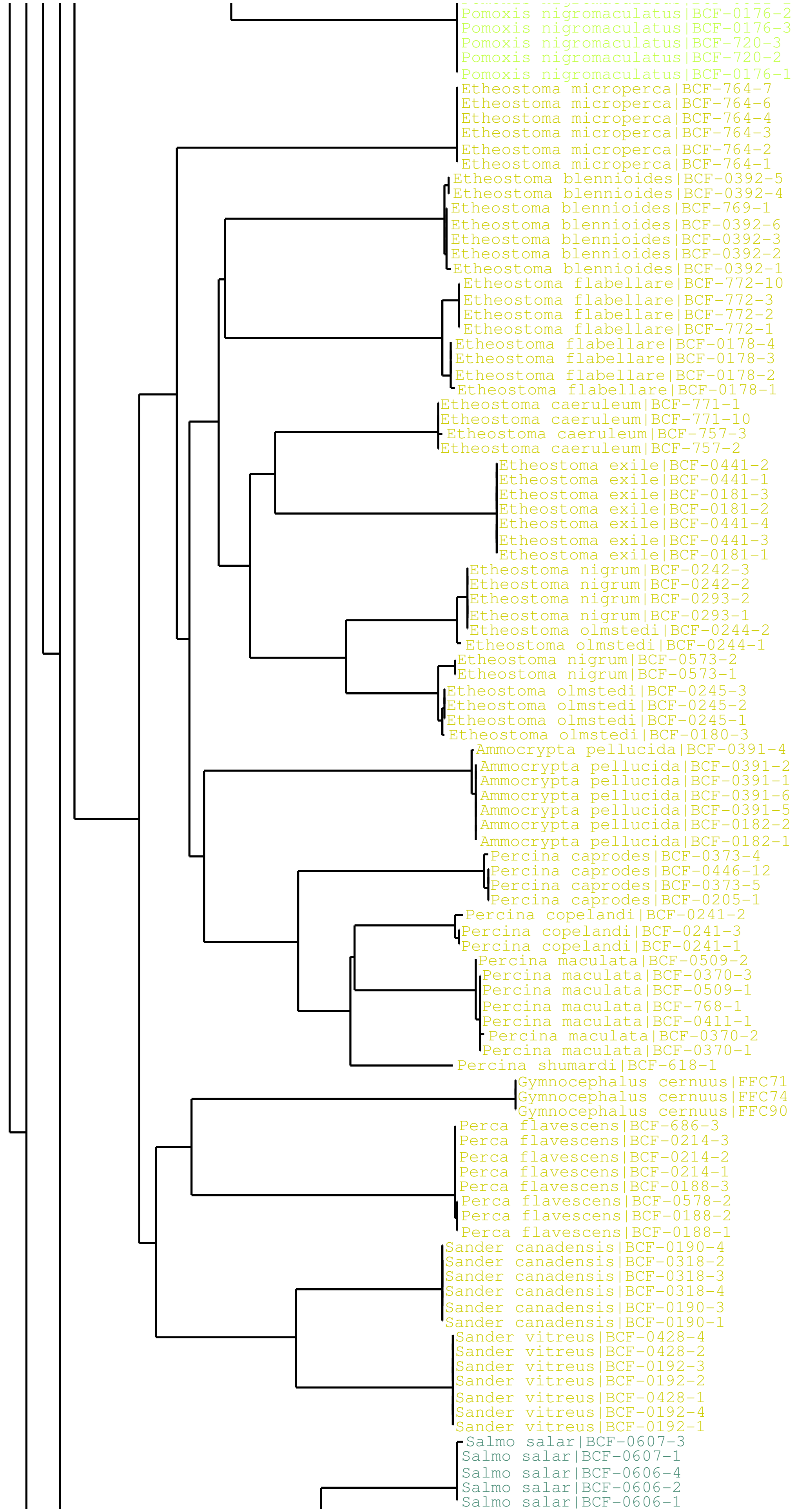
Pomoxis nigromaculatus|BCF-0176-2
Pomoxis nigromaculatus|BCF-0176-3
Pomoxis nigromaculatus|BCF-720-3
Pomoxis nigromaculatus|BCF-720-2
Pomoxis nigromaculatus|BCF-0176-1
Etheostoma microperca|BCF-764-7
Etheostoma microperca|BCF-764-6
Etheostoma microperca|BCF-764-4
Etheostoma microperca|BCF-764-3
Etheostoma microperca|BCF-764-2
Etheostoma microperca|BCF-764-1
Etheostoma blennioides|BCF-0392-5
Etheostoma blennioides|BCF-0392-4
Etheostoma blennioides|BCF-769-1
Etheostoma blennioides|BCF-0392-6
Etheostoma blennioides|BCF-0392-3
Etheostoma blennioides|BCF-0392-2
Etheostoma blennioides|BCF-0392-1
Etheostoma flabellare|BCF-772-10
Etheostoma flabellare|BCF-772-3
Etheostoma flabellare|BCF-772-2
Etheostoma flabellare|BCF-772-1
Etheostoma flabellare|BCF-0178-4
Etheostoma flabellare|BCF-0178-3
Etheostoma flabellare|BCF-0178-2
Etheostoma flabellare|BCF-0178-1
Etheostoma caeruleum|BCF-771-1
Etheostoma caeruleum|BCF-771-10
Etheostoma caeruleum|BCF-757-3
Etheostoma caeruleum|BCF-757-2
Etheostoma exile|BCF-0441-2
Etheostoma exile|BCF-0441-1
Etheostoma exile|BCF-0181-3
Etheostoma exile|BCF-0181-2
Etheostoma exile|BCF-0441-4
Etheostoma exile|BCF-0441-3
Etheostoma exile|BCF-0181-1
Etheostoma nigrum|BCF-0242-3
Etheostoma nigrum|BCF-0242-2
Etheostoma nigrum|BCF-0293-2
Etheostoma nigrum|BCF-0293-1
Etheostoma olmstedi|BCF-0244-2
Etheostoma olmstedi|BCF-0244-1
Etheostoma nigrum|BCF-0573-2
Etheostoma nigrum|BCF-0573-1
Etheostoma olmstedi|BCF-0245-3
Etheostoma olmstedi|BCF-0245-2
Etheostoma olmstedi|BCF-0245-1
Etheostoma olmstedi|BCF-0180-3
Ammocrypta pellucida|BCF-0391-4
Ammocrypta pellucida|BCF-0391-2
Ammocrypta pellucida|BCF-0391-1
Ammocrypta pellucida|BCF-0391-6
Ammocrypta pellucida|BCF-0391-5
Ammocrypta pellucida|BCF-0182-2
Ammocrypta pellucida|BCF-0182-1
Percina caprodes|BCF-0373-4
Percina caprodes|BCF-0446-12
Percina caprodes|BCF-0373-5
Percina caprodes|BCF-0205-1
Percina copelandi|BCF-0241-2
Percina copelandi|BCF-0241-3
Percina copelandi|BCF-0241-1
Percina maculata|BCF-0509-2
Percina maculata|BCF-0370-3
Percina maculata|BCF-0509-1
Percina maculata|BCF-768-1
Percina maculata|BCF-0411-1
Percina maculata|BCF-0370-2
Percina maculata|BCF-0370-1
Percina shumardi|BCF-618-1
Gymnocephalus cernuus|FFC71
Gymnocephalus cernuus|FFC74
Gymnocephalus cernuus|FFC90
Perca flavescens|BCF-686-3
Perca flavescens|BCF-0214-3
Perca flavescens|BCF-0214-2
Perca flavescens|BCF-0214-1
Perca flavescens|BCF-0188-3
Perca flavescens|BCF-0578-2
Perca flavescens|BCF-0188-2
Perca flavescens|BCF-0188-1
Sander canadensis|BCF-0190-4
Sander canadensis|BCF-0318-2
Sander canadensis|BCF-0318-3
Sander canadensis|BCF-0318-4
Sander canadensis|BCF-0190-3
Sander canadensis|BCF-0190-1
Sander vitreus|BCF-0428-4
Sander vitreus|BCF-0428-2
Sander vitreus|BCF-0192-3
Sander vitreus|BCF-0192-2
Sander vitreus|BCF-0428-1
Sander vitreus|BCF-0192-4
Sander vitreus|BCF-0192-1
Salmo salar|BCF-0607-3
Salmo salar|BCF-0607-1
Salmo salar|BCF-0606-4
Salmo salar|BCF-0606-2
Salmo salar|BCF-0606-1

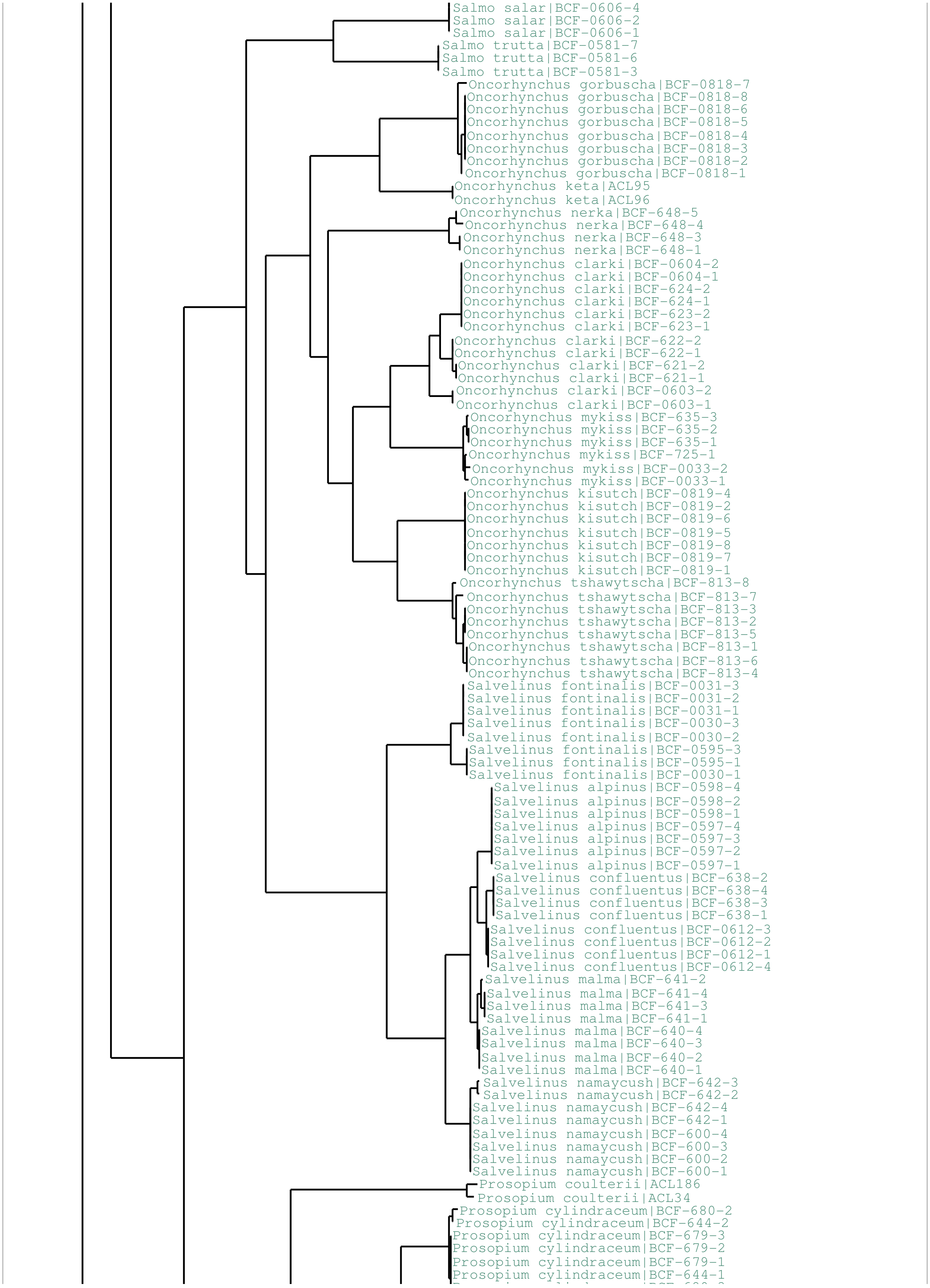
Salmo salar|BCF-0606-4
Salmo salar|BCF-0606-2
Salmo salar|BCF-0606-1
Salmo trutta|BCF-0581-7
Salmo trutta|BCF-0581-6
Salmo trutta|BCF-0581-3
Oncorhynchus gorbuscha|BCF-0818-7
Oncorhynchus gorbuscha|BCF-0818-8
Oncorhynchus gorbuscha|BCF-0818-6
Oncorhynchus gorbuscha|BCF-0818-5
Oncorhynchus gorbuscha|BCF-0818-4
Oncorhynchus gorbuscha|BCF-0818-3
Oncorhynchus gorbuscha|BCF-0818-2
Oncorhynchus gorbuscha|BCF-0818-1
Oncorhynchus keta|ACL95
Oncorhynchus keta|ACL96
Oncorhynchus nerka|BCF-648-5
Oncorhynchus nerka|BCF-648-4
Oncorhynchus nerka|BCF-648-3
Oncorhynchus nerka|BCF-648-1
Oncorhynchus clarki|BCF-0604-2
Oncorhynchus clarki|BCF-0604-1
Oncorhynchus clarki|BCF-624-2
Oncorhynchus clarki|BCF-624-1
Oncorhynchus clarki|BCF-623-2
Oncorhynchus clarki|BCF-623-1
Oncorhynchus clarki|BCF-622-2
Oncorhynchus clarki|BCF-622-1
Oncorhynchus clarki|BCF-621-2
Oncorhynchus clarki|BCF-621-1
Oncorhynchus clarki|BCF-0603-2
Oncorhynchus clarki|BCF-0603-1
Oncorhynchus mykiss|BCF-635-3
Oncorhynchus mykiss|BCF-635-2
Oncorhynchus mykiss|BCF-635-1
Oncorhynchus mykiss|BCF-725-1
Oncorhynchus mykiss|BCF-0033-2
Oncorhynchus mykiss|BCF-0033-1
Oncorhynchus kisutch|BCF-0819-4
Oncorhynchus kisutch|BCF-0819-2
Oncorhynchus kisutch|BCF-0819-6
Oncorhynchus kisutch|BCF-0819-5
Oncorhynchus kisutch|BCF-0819-8
Oncorhynchus kisutch|BCF-0819-7
Oncorhynchus kisutch|BCF-0819-1
Oncorhynchus tshawytscha|BCF-813-8
Oncorhynchus tshawytscha|BCF-813-7
Oncorhynchus tshawytscha|BCF-813-3
Oncorhynchus tshawytscha|BCF-813-2
Oncorhynchus tshawytscha|BCF-813-5
Oncorhynchus tshawytscha|BCF-813-1
Oncorhynchus tshawytscha|BCF-813-6
Oncorhynchus tshawytscha|BCF-813-4
Salvelinus fontinalis|BCF-0031-3
Salvelinus fontinalis|BCF-0031-2
Salvelinus fontinalis|BCF-0031-1
Salvelinus fontinalis|BCF-0030-3
Salvelinus fontinalis|BCF-0030-2
Salvelinus fontinalis|BCF-0595-3
Salvelinus fontinalis|BCF-0595-1
Salvelinus fontinalis|BCF-0030-1
Salvelinus alpinus|BCF-0598-4
Salvelinus alpinus|BCF-0598-2
Salvelinus alpinus|BCF-0598-1
Salvelinus alpinus|BCF-0597-4
Salvelinus alpinus|BCF-0597-3
Salvelinus alpinus|BCF-0597-2
Salvelinus alpinus|BCF-0597-1
Salvelinus confluentus|BCF-638-2
Salvelinus confluentus|BCF-638-4
Salvelinus confluentus|BCF-638-3
Salvelinus confluentus|BCF-638-1
Salvelinus confluentus|BCF-0612-3
Salvelinus confluentus|BCF-0612-2
Salvelinus confluentus|BCF-0612-1
Salvelinus confluentus|BCF-0612-4
Salvelinus malma|BCF-641-2
Salvelinus malma|BCF-641-4
Salvelinus malma|BCF-641-3
Salvelinus malma|BCF-641-1
Salvelinus malma|BCF-640-4
Salvelinus malma|BCF-640-3
Salvelinus malma|BCF-640-2
Salvelinus malma|BCF-640-1
Salvelinus namaycush|BCF-642-3
Salvelinus namaycush|BCF-642-2
Salvelinus namaycush|BCF-642-4
Salvelinus namaycush|BCF-642-1
Salvelinus namaycush|BCF-600-4
Salvelinus namaycush|BCF-600-3
Salvelinus namaycush|BCF-600-2
Salvelinus namaycush|BCF-600-1
Prosopium coulterii|ACL186
Prosopium coulterii|ACL34
Prosopium cylindraceum|BCF-680-2
Prosopium cylindraceum|BCF-644-2
Prosopium cylindraceum|BCF-679-3
Prosopium cylindraceum|BCF-679-2
Prosopium cylindraceum|BCF-679-1
Prosopium cylindraceum|BCF-644-1

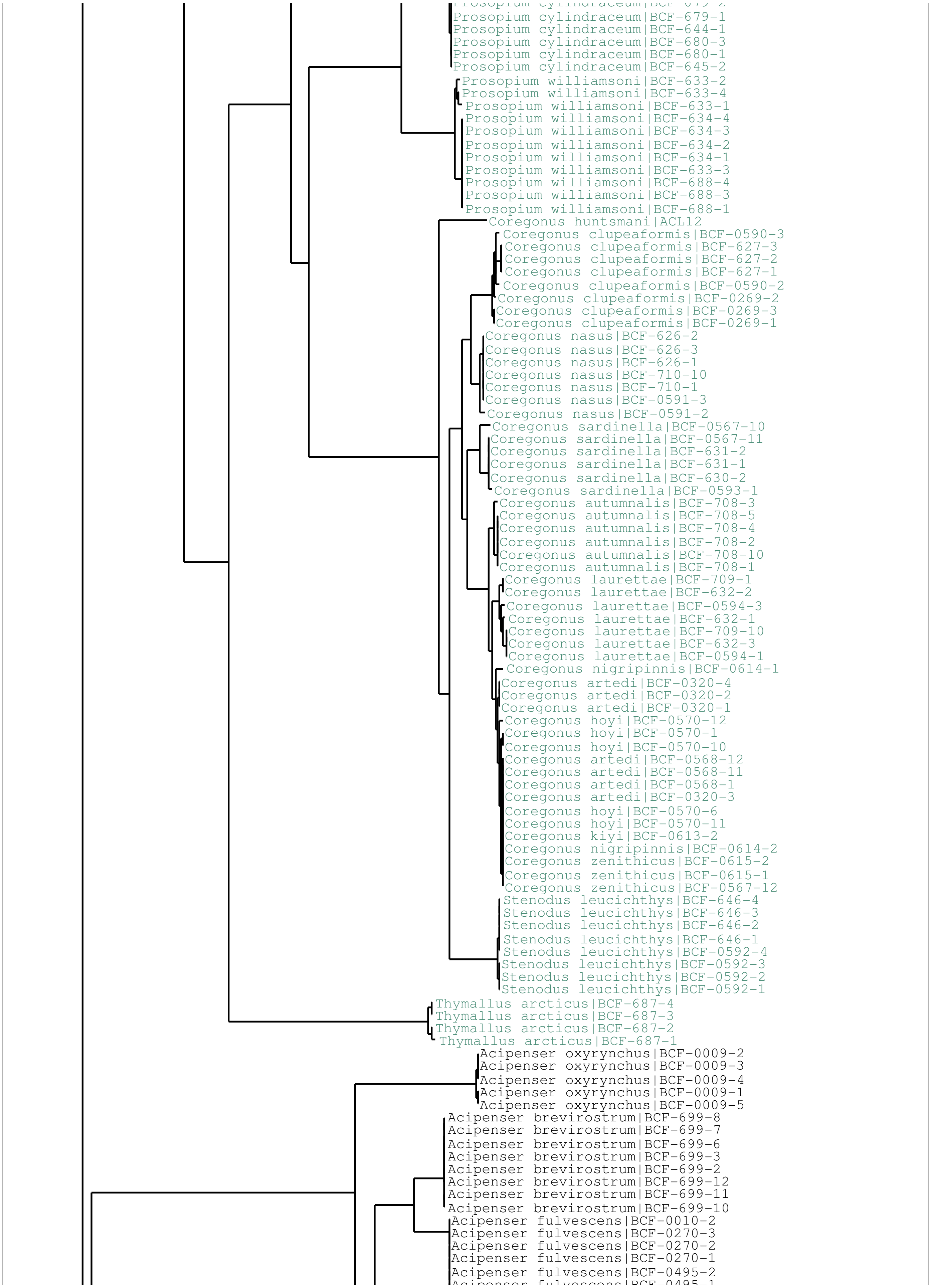

Prosopium cylindraceum|BCF-679-1
Prosopium cylindraceum|BCF-644-1
Prosopium cylindraceum|BCF-680-3
Prosopium cylindraceum|BCF-680-1
Prosopium cylindraceum|BCF-645-2
Prosopium williamsoni|BCF-633-2
Prosopium williamsoni|BCF-633-4
Prosopium williamsoni|BCF-633-1
Prosopium williamsoni|BCF-634-4
Prosopium williamsoni|BCF-634-3
Prosopium williamsoni|BCF-634-2
Prosopium williamsoni|BCF-634-1
Prosopium williamsoni|BCF-633-3
Prosopium williamsoni|BCF-688-4
Prosopium williamsoni|BCF-688-3
Prosopium williamsoni|BCF-688-1
Coregonus huntsmani|ACL12
Coregonus clupeaformis|BCF-0590-3
Coregonus clupeaformis|BCF-627-3
Coregonus clupeaformis|BCF-627-2
Coregonus clupeaformis|BCF-627-1
Coregonus clupeaformis|BCF-0590-2
Coregonus clupeaformis|BCF-0269-2
Coregonus clupeaformis|BCF-0269-3
Coregonus clupeaformis|BCF-0269-1
Coregonus nasus|BCF-626-2
Coregonus nasus|BCF-626-3
Coregonus nasus|BCF-626-1
Coregonus nasus|BCF-710-10
Coregonus nasus|BCF-710-1
Coregonus nasus|BCF-0591-3
Coregonus nasus|BCF-0591-2
Coregonus sardinella|BCF-0567-10
Coregonus sardinella|BCF-0567-11
Coregonus sardinella|BCF-631-2
Coregonus sardinella|BCF-631-1
Coregonus sardinella|BCF-630-2
Coregonus sardinella|BCF-0593-1
Coregonus autumnalis|BCF-708-3
Coregonus autumnalis|BCF-708-5
Coregonus autumnalis|BCF-708-4
Coregonus autumnalis|BCF-708-2
Coregonus autumnalis|BCF-708-10
Coregonus autumnalis|BCF-708-1
Coregonus laurettae|BCF-709-1
Coregonus laurettae|BCF-632-2
Coregonus laurettae|BCF-0594-3
Coregonus laurettae|BCF-632-1
Coregonus laurettae|BCF-709-10
Coregonus laurettae|BCF-632-3
Coregonus laurettae|BCF-0594-1
Coregonus nigripinnis|BCF-0614-1
Coregonus artedi|BCF-0320-4
Coregonus artedi|BCF-0320-2
Coregonus artedi|BCF-0320-1
Coregonus hoyi|BCF-0570-12
Coregonus hoyi|BCF-0570-1
Coregonus hoyi|BCF-0570-10
Coregonus artedi|BCF-0568-12
Coregonus artedi|BCF-0568-11
Coregonus artedi|BCF-0568-1
Coregonus artedi|BCF-0320-3
Coregonus hoyi|BCF-0570-6
Coregonus hoyi|BCF-0570-11
Coregonus kiyi|BCF-0613-2
Coregonus nigripinnis|BCF-0614-2
Coregonus zenithicus|BCF-0615-2
Coregonus zenithicus|BCF-0615-1
Coregonus zenithicus|BCF-0567-12
Stenodus leucichthys|BCF-646-4
Stenodus leucichthys|BCF-646-3
Stenodus leucichthys|BCF-646-2
Stenodus leucichthys|BCF-646-1
Stenodus leucichthys|BCF-0592-4
Stenodus leucichthys|BCF-0592-3
Stenodus leucichthys|BCF-0592-2
Stenodus leucichthys|BCF-0592-1
Thymallus arcticus|BCF-687-4
Thymallus arcticus|BCF-687-3
Thymallus arcticus|BCF-687-2
Thymallus arcticus|BCF-687-1
Acipenser oxyrynchus|BCF-0009-2
Acipenser oxyrynchus|BCF-0009-3
Acipenser oxyrynchus|BCF-0009-4
Acipenser oxyrynchus|BCF-0009-1
Acipenser oxyrynchus|BCF-0009-5
Acipenser brevirostrum|BCF-699-8
Acipenser brevirostrum|BCF-699-7
Acipenser brevirostrum|BCF-699-6
Acipenser brevirostrum|BCF-699-3
Acipenser brevirostrum|BCF-699-2
Acipenser brevirostrum|BCF-699-12
Acipenser brevirostrum|BCF-699-11
Acipenser brevirostrum|BCF-699-10
Acipenser fulvescens|BCF-0010-2
Acipenser fulvescens|BCF-0270-3
Acipenser fulvescens|BCF-0270-2
Acipenser fulvescens|BCF-0270-1
Acipenser fulvescens|BCF-0495-2

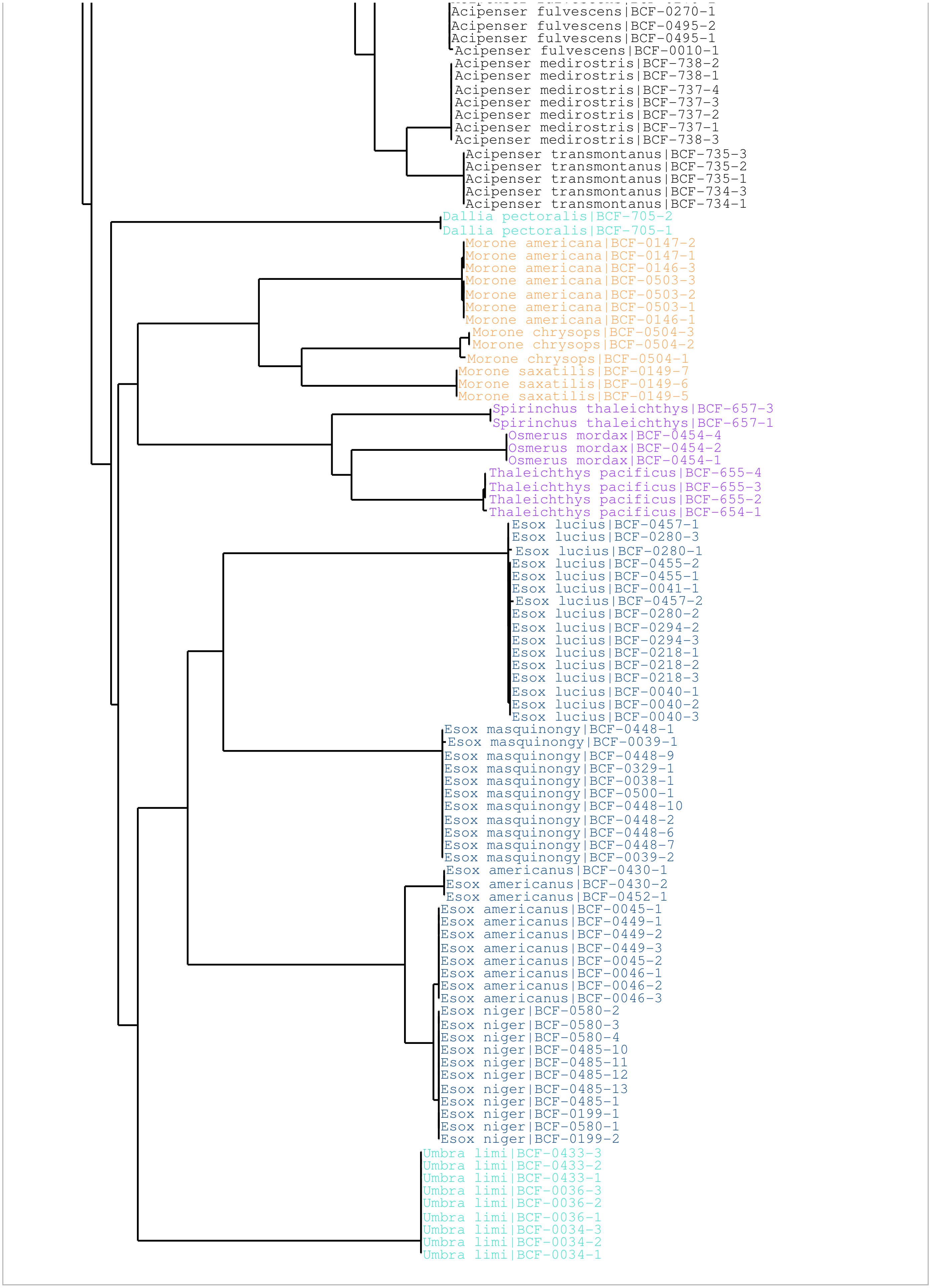
Acipenser fulvescens|BCF-0270-1
Acipenser fulvescens|BCF-0495-2
Acipenser fulvescens|BCF-0495-1
Acipenser fulvescens|BCF-0010-1
Acipenser medirostris|BCF-738-2
Acipenser medirostris|BCF-738-1
Acipenser medirostris|BCF-737-4
Acipenser medirostris|BCF-737-3
Acipenser medirostris|BCF-737-2
Acipenser medirostris|BCF-737-1
Acipenser medirostris|BCF-738-3
Acipenser transmontanus|BCF-735-3
Acipenser transmontanus|BCF-735-2
Acipenser transmontanus|BCF-735-1
Acipenser transmontanus|BCF-734-3
Acipenser transmontanus|BCF-734-1
Dallia pectoralis|BCF-705-2
Dallia pectoralis|BCF-705-1
Morone americana|BCF-0147-2
Morone americana|BCF-0147-1
Morone americana|BCF-0146-3
Morone americana|BCF-0503-3
Morone americana|BCF-0503-2
Morone americana|BCF-0503-1
Morone americana|BCF-0146-1
Morone chrysops|BCF-0504-3
Morone chrysops|BCF-0504-2
Morone chrysops|BCF-0504-1
Morone saxatilis|BCF-0149-7
Morone saxatilis|BCF-0149-6
Morone saxatilis|BCF-0149-5
Spirinchus thaleichthys|BCF-657-3
Spirinchus thaleichthys|BCF-657-1
Osmerus mordax|BCF-0454-4
Osmerus mordax|BCF-0454-2
Osmerus mordax|BCF-0454-1
Thaleichthys pacificus|BCF-655-4
Thaleichthys pacificus|BCF-655-3
Thaleichthys pacificus|BCF-655-2
Thaleichthys pacificus|BCF-654-1
Esox lucius|BCF-0457-1
Esox lucius|BCF-0280-3
Esox lucius|BCF-0280-1
Esox lucius|BCF-0455-2
Esox lucius|BCF-0455-1
Esox lucius|BCF-0041-1
Esox lucius|BCF-0457-2
Esox lucius|BCF-0280-2
Esox lucius|BCF-0294-2
Esox lucius|BCF-0294-3
Esox lucius|BCF-0218-1
Esox lucius|BCF-0218-2
Esox lucius|BCF-0218-3
Esox lucius|BCF-0040-1
Esox lucius|BCF-0040-2
Esox lucius|BCF-0040-3
Esox masquinongy|BCF-0448-1
Esox masquinongy|BCF-0039-1
Esox masquinongy|BCF-0448-9
Esox masquinongy|BCF-0329-1
Esox masquinongy|BCF-0038-1
Esox masquinongy|BCF-0500-1
Esox masquinongy|BCF-0448-10
Esox masquinongy|BCF-0448-2
Esox masquinongy|BCF-0448-6
Esox masquinongy|BCF-0448-7
Esox masquinongy|BCF-0039-2
Esox americanus|BCF-0430-1
Esox americanus|BCF-0430-2
Esox americanus|BCF-0452-1
Esox americanus|BCF-0045-1
Esox americanus|BCF-0449-1
Esox americanus|BCF-0449-2
Esox americanus|BCF-0449-3
Esox americanus|BCF-0045-2
Esox americanus|BCF-0046-1
Esox americanus|BCF-0046-2
Esox americanus|BCF-0046-3
Esox niger|BCF-0580-2
Esox niger|BCF-0580-3
Esox niger|BCF-0580-4
Esox niger|BCF-0485-10
Esox niger|BCF-0485-11
Esox niger|BCF-0485-12
Esox niger|BCF-0485-13
Esox niger|BCF-0485-1
Esox niger|BCF-0199-1
Esox niger|BCF-0580-1
Esox niger|BCF-0199-2
Umbra limi|BCF-0433-3
Umbra limi|BCF-0433-2
Umbra limi|BCF-0433-1
Umbra limi|BCF-0036-3
Umbra limi|BCF-0036-2
Umbra limi|BCF-0036-1
Umbra limi|BCF-0034-3
Umbra limi|BCF-0034-2
Umbra limi|BCF-0034-1
